# Diagnostic Performance and Workup Efficiency of Large Language Models in Secondary Hypertension: A Blinded Comparative Study

**DOI:** 10.3390/diagnostics16142165

**Published:** 2026-07-10

**Authors:** Asena Gökçay Canpolat, Özge Baş Aksu, Rıfat Emral, Uğur Canpolat

**Affiliations:** 1 Department of Endocrinology and Metabolism, Ankara University School of Medicine, Ankara 06230, Turkey; ozgebasaksu@gmail.com (Ö.B.A.); rifatemral@gmail.com (R.E.); 2 Department of Cardiology, Hacettepe University School of Medicine, Ankara 06230, Turkey; dru_canpolat@yahoo.com

**Keywords:** secondary hypertension, large language models, artificial intelligence, clinical decision support, diagnostic reasoning, chatbot comparison, hallucination, diagnostic workup efficiency

## Abstract

**Background/Objectives**: Secondary hypertension (SH) requires complex diagnostic reasoning and guideline-based management, posing substantial challenges for artificial intelligence–driven clinical decision-support systems. This study aimed to comparatively evaluate the performance of three large language models (LLMs) in diagnostic reasoning, clinical management, follow-up planning, and patient-oriented communication in SH. **Methods**: In this cross-sectional blinded study, three LLMs—GPT-5.2 (OpenAI), Claude Sonnet 4.6 (Anthropic), and Gemini 3.0 Pro (Google)—were evaluated using 10 expert-developed clinical case vignettes representing major etiologies of SH. Model outputs were anonymized and independently assessed by three senior clinicians (two endocrinologists and one cardiologist) using a 7-point Likert scale across five domains: (1) diagnostic accuracy and hallucination control, (2) quality and comprehensiveness, (3) reliability and clinical guidance, (4) efficiency of diagnostic workup, and (5) clinical usability. Group differences were analyzed using Kruskal–Wallis tests with Bonferroni-corrected pairwise comparisons. Inter-rater agreement was assessed using two-way mixed-effects intraclass correlation coefficients with absolute agreement. **Results**: A total of 90 blinded expert evaluations were analyzed. GPT-5.2 (6.0, Q1–Q3 5.40–6.05) and Gemini 3 Pro (5.2, Q1–Q3 4.55–6.20) (H = 40.055, *p* < 0.001). The results indicated a clear performance hierarchy, with Claude Sonnet 4.6 receiving the highest overall scores, followed by GPT-5.2 and Gemini 3 Pro. Pairwise analyses showed higher scores for Claude Sonnet 4.6 than the other models in most domains, while efficiency of diagnostic workup showed smaller between-model differences. GPT-5.2 generally showed intermediate performance, with higher ratings than Gemini 3 Pro in reliability and clinical usability. Performance differences were most pronounced in domains requiring complex clinical reasoning, whereas efficiency of diagnostic workup scores was relatively comparable among models. Claude Sonnet 4.6 ranked first in nine of the ten clinical vignettes. Inter-rater agreement analyses demonstrated consistent ranking patterns among evaluators. **Conclusions**: These exploratory findings suggest heterogeneous and model-dependent performance of LLMs in secondary hypertension–related clinical tasks. A clear clinician-rated performance hierarchy was observed, with differences most apparent in domains requiring complex clinical reasoning. However, given the pilot vignette-based design and limited sample size, these results should be interpreted as hypothesis-generating and require confirmation in larger, multicenter validation studies before routine clinical implementation can be considered.

## 1. Introduction

Secondary hypertension (SH) represents a heterogeneous group of disorders in which elevated blood pressure results from an identifiable and potentially reversible underlying cause. It accounts for approximately 5–10% of hypertension cases in the general population and up to 20–30% among patients with resistant hypertension, early-onset hypertension, or abrupt deterioration of previously stable blood pressure control [1,2]. Common etiologies include primary aldosteronism, renovascular disease, chronic kidney disease, obstructive sleep apnea, pheochromocytoma, Cushing’s syndrome, thyroid disorders, and medication- or substance-induced hypertension. Early recognition of these conditions is clinically important because targeted therapy may substantially improve blood pressure control, reduce long-term cardiovascular and renal morbidity, and, in selected cases, provide a definitive cure [3,4]. Beyond improving patient outcomes, accurate identification of SH may also decrease healthcare utilization associated with ineffective treatment escalation, unnecessary diagnostic procedures, and preventable complications related to prolonged uncontrolled hypertension.

Despite its clinical importance, the diagnostic evaluation of SH remains complex and is frequently underutilized in routine practice. Clinical manifestations are often nonspecific, and differentiating secondary forms from essential hypertension may be challenging, particularly in patients with mild-to-moderate blood pressure elevation. In addition, screening recommendations vary among international guidelines, creating uncertainty regarding patient selection and optimal testing strategies [2,5]. Biochemical evaluation is further complicated by pre-analytical variability, assay limitations, and the confounding effects of commonly prescribed antihypertensive medications on renin–angiotensin–aldosterone axis measurements. Imaging studies, although informative, may reveal incidental findings that do not necessarily indicate causality. Together, these diagnostic ambiguities increase the cognitive burden on clinicians and may contribute to delayed diagnosis, inappropriate testing, or missed opportunities for curative interventions.

Accordingly, SH represents a clinical domain in which structured reasoning, guideline familiarity, and integrative decision-making are essential. These challenges are particularly relevant in busy outpatient settings and non-specialized centers where access to endocrine hypertension expertise may be limited. Decision-support systems capable of synthesizing complex clinical information and suggesting evidence-based diagnostic pathways could therefore improve diagnostic consistency, reduce unnecessary investigations, and facilitate earlier referral of high-risk patients.

Recent advances in artificial intelligence have introduced large language models (LLMs) as emerging tools with potential applications in medical education, diagnostic reasoning, clinical decision support, and patient-oriented communication. Trained on large-scale text corpora, LLMs can generate context-aware responses that simulate human-like reasoning. In healthcare settings, these systems have demonstrated the ability to synthesize medical knowledge, generate differential diagnoses, interpret clinical scenarios, and assist in management planning. Systematic reviews and simulation-based evaluations suggest that LLMs may achieve near-expert performance in selected medical tasks, particularly when structured prompts and clearly defined clinical contexts are used [6,7,8,9,10].

Comparative evaluations of major LLM systems including GPT (OpenAI), Claude Sonnet (Anthropic), and Gemini (Google) have demonstrated substantial variability in diagnostic accuracy, reasoning transparency, response consistency, and safety profiles. Differences in training data composition, alignment strategies, reinforcement learning frameworks, and underlying model architectures may contribute to heterogeneous clinical performance [11,12,13,14]. Although some studies have reported high levels of diagnostic concordance with expert clinicians, others have highlighted concerns regarding hallucinated information, incomplete guideline adherence, overconfident recommendations, and instability across repeated queries. These limitations underscore the need for systematic benchmarking of LLMs in clinically realistic scenarios before their integration into clinical decision-support workflows.

Importantly, the potential value of LLMs extends beyond benchmarking performance alone. If shown to provide accurate and consistent clinical reasoning, these systems may eventually support physicians during complex diagnostic evaluations, assist with interpretation of guideline-based algorithms, improve standardization of care across institutions, and enhance patient education regarding diagnostic procedures and treatment strategies. In endocrine hypertension practice, where diagnostic pathways frequently involve multiple sequential tests and nuanced interpretation of biochemical and radiological findings, AI-assisted systems could theoretically reduce diagnostic delays and support more efficient resource utilization. Conversely, inaccurate or overconfident recommendations could directly influence clinical decision-making and potentially expose patients to unnecessary investigations or inappropriate treatments. Therefore, rigorous evaluation of the reliability, consistency, and practical clinical applicability of LLM-generated recommendations is essential before broader implementation in routine care can be considered.

Notably, the existing literature has primarily focused on general internal medicine cases, examination-style question banks, or single-step diagnostic tasks. Data remain limited regarding LLM performance in complex endocrine disorders requiring multistep reasoning, biochemical interpretation, and adherence to evidence-based diagnostic algorithms. SH provides an ideal evaluation framework because its assessment requires integration of clinical findings, laboratory interpretation, medication effects, imaging strategies, and longitudinal management planning. Furthermore, appropriate care depends on distinguishing among screening, confirmatory testing, subtype classification, and therapeutic decision-making processes that challenge both human clinicians and AI-based systems.

To our knowledge, this study represents one of the first blinded, clinician-led benchmarking investigations specifically evaluating LLM performance in secondary hypertension using standardized clinical prompting conditions and multidimensional expert assessment. While many previous studies have evaluated LLMs using examination-style questions, diagnostic classification tasks, or benchmark datasets, comparatively few have focused on clinician-rated assessment of complex endocrine reasoning, longitudinal management planning, and workflow-oriented clinical usability.

Therefore, the present study aimed to perform a blinded head-to-head comparison of three widely used LLMs across key domains of endocrine clinical reasoning related to SH. This study aims to provide a structured clinical evaluation of currently available LLMs in the diagnostic workup of secondary hypertension, a high-risk subspecialty domain where inappropriate recommendations may lead to delayed diagnosis, unnecessary investigations, or unsafe management. Specifically, we evaluated their performance in diagnostic reasoning, follow-up planning, therapeutic decision-making, patient-oriented communication, and efficiency of diagnostic workup. By assessing clinical accuracy, inter-model consistency, and practical usability, this study seeks to clarify the current capabilities and limitations of LLMs as supportive tools in real-world endocrine and hypertension practice, while also exploring their potential implications for diagnostic standardization, clinical decision-making, and patient-centered care.

Accordingly, the contribution of the present study should be interpreted not as the development of a novel artificial intelligence methodology, but as a structured clinical performance and safety assessment of widely accessible general-purpose LLMs in a complex endocrine-hypertension domain. Although these models were not specifically designed for secondary hypertension, this represents a clinically relevant question because such systems are already accessible to clinicians, trainees, and patients and may be used for complex medical reasoning without domain-specific optimization. Therefore, evaluating their current performance helps identify domain-specific strengths, limitations, and potential safety concerns before any broader clinical use can be considered. More importantly, the study contributes to knowledge by showing that the apparent general medical reasoning ability of LLMs cannot be assumed to transfer uniformly to complex subspecialty tasks, and that domain-specific validation remains necessary before clinical decision-support implementation.

## 2. Materials and Methods

### 2.1. Study Design and Ethical Considerations

This cross-sectional, blinded, head-to-head comparative study was designed to evaluate the clinical performance of three state-of-the-art LLMs in the diagnostic evaluation and management of SH. The study framework was structured to simulate real-world clinical reasoning tasks encountered in endocrine and hypertension practice. All assessments were performed using standardized hypothetical case vignettes rather than real patient data.

Because the study did not involve human participants, identifiable patient information, biological materials, or animal experimentation, formal ethical committee approval and informed consent were not required. This approach is consistent with previously published simulation-based evaluations of artificial intelligence systems in clinical medicine. The study adhered to principles of transparency, reproducibility, and methodological neutrality in AI benchmarking.

### 2.2. Model Selection and Anonymization

Three widely used and commercially available LLMs representing different architectural frameworks and training methodologies were selected for evaluation:

- GPT-5.2: OpenAI (San Francisco, CA, USA)
- Claude Sonnet 4.6: Anthropic (San Francisco, CA, USA)

Gemini 3 Pro: Google LLC (Mountain View, CA, USA)To reduce potential evaluator bias associated with brand recognition, model identities were anonymized prior to scoring. Each model was assigned a neutral elemental code name (Water, Earth, Air), and all outputs were reformatted to remove stylistic identifiers (Supplementary Table S1). Evaluators remained fully blinded to model identity throughout the assessment process. Although outputs were anonymized and reformatted to minimize stylistic identifiers, the possibility of partial de-anonymization cannot be fully excluded. Experienced clinicians familiar with contemporary LLMs may potentially recognize model-specific stylistic patterns, including formatting preferences, response organization, hedging language, or explanatory structure. Formal assessment of blinding success was not performed after evaluation; however, evaluators were not informed of model allocation, model order was randomized, and all outputs were standardized in appearance to reduce potential recognition bias.

Model selection was based on:

1. Widespread clinical and academic usage;
2. Advanced multimodal reasoning capabilities;
3. Public accessibility and reproducibility;
4. Representation of distinct AI training paradigms.

### 2.3. Case Vignette Development

Ten high-fidelity clinical case vignettes were developed collaboratively by a multidisciplinary panel consisting of two endocrinologists and one cardiologist with extensive expertise in hypertension and endocrine disorders. Cases were designed to reflect realistic diagnostic scenarios encountered in tertiary endocrine referral centers.

Each vignette was constructed to include:

- Demographic characteristics;
- Presenting symptoms and physical examination findings;
- Relevant laboratory results;
- Imaging findings where appropriate;
- Medication history;
- Key clinical decision points.

Vignettes collectively represented major etiological categories of SH:

1. Primary aldosteronism;
2. Pheochromocytoma/paraganglioma;
3. Atherosclerotic renal artery stenosis;
4. Fibromuscular dysplasia;
5. Primary hyperparathyroidism;
6. Obstructive sleep apnea;
7. Coarctation of the aorta;
8. Cushing’s syndrome;
9. Renal parenchymal disease;
10. Mixed or atypical presentations requiring complex differential diagnosis.

Case complexity was intentionally varied to test model performance across straightforward, intermediate, and diagnostically challenging scenarios requiring multistep reasoning. Characteristics of the clinical vignettes included in the study are included in Supplementary Table S1.

### 2.4. Prompt Standardization and Model Querying

To ensure methodological consistency, all LLMs were queried within the same time window (17 February 2026) using default model configurations without temperature adjustment or external tool augmentation. A standardized, high-fidelity English prompt was developed to minimize prompt-induced variability (Supplementary File S2). The prompt instructed each model to assume the role of a “board-certified specialist in endocrinology and hypertension” and to provide structured clinical reasoning. No iterative prompting, clarification requests, or response regeneration were permitted. Each vignette was submitted once to each model in an independent session to prevent memory contamination across cases.

To enhance reproducibility, identical prompts, vignette formatting, and query conditions were applied across all models (Supplementary File S2). The complete prompting template was fixed before initiation of the study and remained unchanged throughout all evaluations. All model outputs were generated during a predefined study period to minimize potential variability related to interim model updates or platform modifications. In addition, separate chat sessions were used for each vignette to reduce contextual carryover effects and maintain independence between cases.

Because LLM-generated responses may vary across repeated queries due to stochastic generation processes, response variability was considered during study design. However, the primary aim of the present study was to evaluate comparative performance under standardized real-world single-query conditions rather than response stability across multiple iterations. Therefore, repeated querying and consensus-response generation were intentionally not performed. This approach was selected to better simulate routine clinical usage scenarios in which clinicians typically interact with AI systems using a single prompt-response exchange. Nevertheless, the authors acknowledge that intra-model variability may influence reproducibility of outputs and represents an important area for future investigation.

Model responses were required to follow a predefined structured format consisting of five clinical reasoning domains:

1. Diagnosis and Differential Diagnosis;
2. Diagnostic Workup Strategy;
3. Acute and Long-Term Management Plan;
4. Follow-up and Monitoring Strategy;
5. Patient-Oriented Education and Counseling.

Outputs exceeding predefined length thresholds were truncated to ensure comparable evaluation conditions.

To facilitate independent replication and methodological transparency, the complete verbatim prompt template, including role instructions, formatting constraints, and structured output requirements, is provided in the Supplementary Materials (Supplementary File S2). The study design did not permit external document retrieval, internet browsing, or citation verification beyond each model’s native capabilities at the time of querying.

### 2.5. Evaluation Framework and Scoring System

#### 2.5.1. Evaluator Panel

Blinded evaluations were performed independently by three senior clinicians:

- Two board-certified endocrinologists;
- One board-certified cardiologist.

Clinical experience ranged from 10 to 30 years in tertiary referral centers. All evaluators routinely manage patients with complex hypertension and endocrine disorders. Before scoring, evaluators independently reviewed and discussed two pilot vignettes before formal scoring to harmonize interpretation of domain definitions and reduce inter-observer variability.

The evaluation framework was designed to prioritize clinically meaningful assessment of AI-generated recommendations rather than technical characterization of model architectures or computational performance.

#### 2.5.2. Scoring Domains

Each model response was assessed using a 7-point Likert scale (1 = poorest performance; 7 = excellent performance) across five predefined domains:

**1. Accuracy and Hallucination Control**

Assessed factual correctness, internal consistency, and concordance with widely accepted contemporary international guidelines (ESH/ESC hypertension guidelines and Endocrine Society recommendations).

**2. Quality and Comprehensiveness of Clinical Reasoning**

Evaluated logical structure, pathophysiological reasoning, completeness of differential diagnosis, and integration of clinical data.

**3. Reliability and Safety of Clinical Guidance**

Assessed whether recommendations were safe, clinically appropriate, and free of potentially harmful or misleading suggestions.

**4. Efficiency of Diagnostic Workup**

Evaluated clinician-perceived appropriateness and efficiency of the proposed diagnostic strategy, including prioritization of high-yield tests and avoidance of unnecessary investigations, rather than formal economic cost-effectiveness analysis.

**5. Clinical Usability and Practical Applicability**

Assessed clarity, organization, and suitability for real-world clinical implementation by practicing physicians.

Table 1 illustrated how each prompted task contributed to the multidimensional evaluation framework.

For the purposes of this study, hallucination was operationally defined as generation of clinically unsupported, fabricated, internally inconsistent, or non-guideline-concordant diagnostic or management recommendations lacking appropriate justification based on the provided vignette data. Hallucination assessment was performed qualitatively through expert clinical review rather than automated computational detection algorithms. Evaluators identified hallucinations based on inconsistency with established clinical guidelines, unsupported diagnostic assertions, fabricated investigations or treatments, internal contradictions, or clinically inappropriate recommendations.

#### 2.5.3. Global Preference Assessment

In addition to domain scoring, evaluators selected a “Global Favorite” model for each vignette. This forced-choice metric identified the model providing the most trustworthy and clinically useful overall decision support.

#### 2.5.4. Supplementary Checklist-Based Evaluation

To reduce reliance on subjective global scoring, an additional checklist-based evaluation was performed as a supplementary analysis. For each clinical vignette, the expert panel defined a set of expected guideline-relevant clinical elements. These elements included the correct leading diagnosis, important differential diagnoses, essential diagnostic workup steps, appropriate confirmatory testing, relevant imaging or subtype evaluation when applicable, medication-related considerations, management principles, follow-up recommendations, and potentially unsafe or unsupported recommendations. Each original model response was assessed for the presence or absence of these predefined elements. For supplementary binary analysis, expert scores were dichotomized as present/adequate performance. Scores ≥ 5 were classified as present/adequate, while scores < 5 were classified as absent/not adequate. This binary transformation was applied to both composite scores and individual domain scores. For each model, frequencies and percentages of present/adequate ratings were calculated across evaluator–vignette assessments. A stricter threshold of ≥6 was additionally used as a sensitivity analysis to identify outputs with strong clinical performance (Supplementary File S2).

### 2.6. Statistical Analysis

All statistical analyses were performed using IBM SPSS Statistics version 25 (IBM Corp., Armonk, NY, USA). Because the evaluation data comprised ordinal 7-point Likert-scale scores, the Kolmogorov–Smirnov test was used to assess normality, which indicated non-normal distribution patterns across multiple evaluation domains. Non-parametric methods were therefore applied consistently throughout the study, and all results are reported as median and the first-to-third quartile range (Q1–Q3). Differences in median domain scores among the three LLMs were evaluated using the Kruskal–Wallis H test. When significant omnibus differences were detected, pairwise post hoc comparisons were performed using the Mann–Whitney U test. To control for Type I error inflation due to multiple comparisons, Bonferroni correction was applied, establishing an adjusted significance threshold of: α = 0.05/3 = 0.017. A composite performance score was derived by calculating the mean of the five domain scores for each observation. Inter-model differences in composite scores were analyzed using the same non-parametric procedures. Agreement among evaluators was assessed using a two-way mixed-effects intraclass correlation coefficient (ICC) model with absolute agreement definition. ICC values were interpreted as:

▪ <0.50 → Poor reliability;
▪ 0.50–0.75 → Moderate reliability;
▪ 0.75–0.90 → Good reliability;
▪ >0.90 → Excellent reliability.

Case-level composite scores were summarized descriptively and visualized using heatmaps to illustrate performance patterns across diagnostic categories. Domain-level profiles were additionally displayed using radar and forest plots to facilitate multidimensional comparison among models. These visualizations were intended for descriptive interpretation only and were not used for inferential statistical testing.

No formal a priori power analysis was performed because this study was designed as an exploratory, simulation-based benchmarking analysis rather than a hypothesis-driven clinical trial. The sample size of 10 vignettes was selected pragmatically to represent major etiological categories of SH while maintaining feasibility for blinded expert evaluation. Accordingly, statistical findings should be interpreted as comparative and hypothesis-generating rather than definitive estimates of model performance.

Statistical significance thresholds were defined as: *p* < 0.05 for omnibus comparisons and *p* < 0.017 for Bonferroni-adjusted pairwise tests. All tests were two-tailed.

## 3. Results

A total of 90 blinded expert evaluations (three LLMs × 10 clinical vignettes × three independent evaluators) were analyzed across five predefined domains using a 7-point Likert scoring system. All responses were independently rated by three senior clinicians with extensive experience in tertiary endocrine and cardiovascular care, including two endocrinologists (A.G.C., R.E.) and one cardiologist (U.C.).

### 3.1. Overall Model Performance

Composite performance scores demonstrated statistically significant differences among models (Kruskal–Wallis H = 40.055, *p* < 0.001), indicating heterogeneous overall clinical performance.

Claude Sonnet 4.6 achieved the highest composite score (median 6.8, Q1–Q3 6.20–7.00), followed by GPT-5.2 (median 6.0, Q1–Q3 5.40–6.05) and Gemini 3 Pro (median 5.2, Q1–Q3 4.55–6.20).

Post hoc pairwise comparisons revealed:

- Claude Sonnet 4.6 significantly outperformed GPT-5.2 (U = 115, *p* < 0.001);
- Claude Sonnet 4.6 significantly outperformed Gemini 3 Pro (U = 77.5, *p* < 0.001);
- GPT-5.2 showed numerically higher scores than Gemini 3 Pro (U = 296.5, *p* = 0.023), though this difference did not remain significant after Bonferroni correction.

These findings establish a clear performance hierarchy:

**Claude Sonnet 4.6 > GPT-5.2 > Gemini 3 Pro**

Domain-specific analyses demonstrated statistically significant differences across all five evaluation domains (Table 2).

**Accuracy and Hallucination Control:** Significant inter-model differences were observed (H = 42.443, *p* < 0.001). Claude Sonnet 4.6 demonstrated superior factual accuracy and guideline concordance, significantly outperforming both comparators after Bonferroni adjustment.

Although GPT-5.2 scored higher than Gemini 3 Pro, this difference did not remain statistically significant after correction (*p* = 0.037).

**Quality and Comprehensiveness of Clinical Reasoning:** This domain demonstrated one of the largest effect sizes (H = 39.075, *p* < 0.001). Claude Sonnet 4.6 consistently provided more structured differential diagnoses, deeper pathophysiological integration, and more complete diagnostic strategies.

Pairwise differences between GPT-5.2 and Gemini 3 Pro did not remain significant after correction (*p* = 0.022).

**Reliability and Safety of Clinical Guidance:** Claude Sonnet 4.6 again ranked highest (H = 40.314, *p* < 0.001), reflecting safer therapeutic recommendations and better adherence to guideline-based management pathways.

Notably, GPT-5.2 significantly outperformed Gemini 3 Pro after Bonferroni correction (U = 284, *p* = 0.009), indicating comparatively greater reliability in treatment planning and risk avoidance.

**Efficiency of Diagnostic Workup:** This domain showed the smallest between-group effect size (H = 9.148, *p* = 0.010), suggesting relatively comparable performance across models.

After Bonferroni adjustment:

- Claude Sonnet 4.6 significantly outperformed Gemini 3 Pro (U = 277, *p* = 0.007);
- Claude Sonnet 4.6 and GPT-5.2 demonstrated statistically comparable performance (*p* = 0.032);
- GPT-5.2 and Gemini 3 Pro showed no significant difference.

Overall, all models demonstrated relatively stronger performance in test prioritization compared with other reasoning domains.

**Figure 1:**
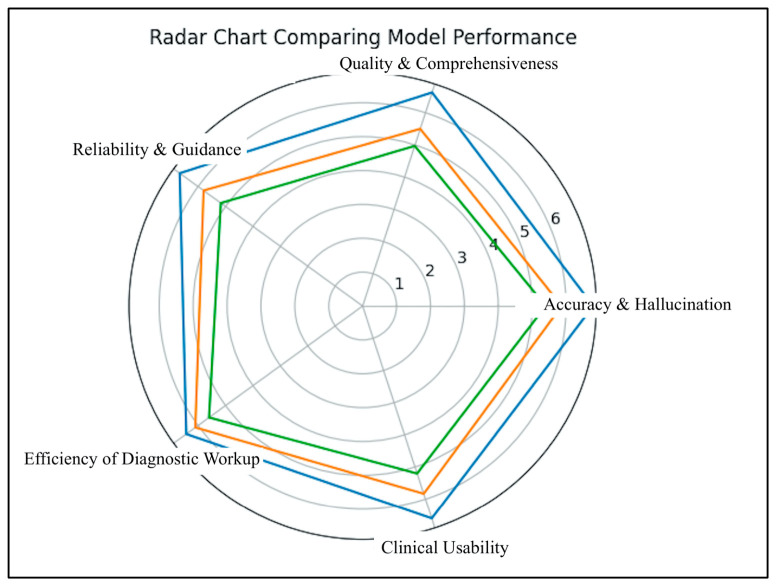
Radar chart illustrating multidimensional performance profiles. The blue line represents Claude Sonnet 4.6, the orange line represents GPT-5.2, and the green line represents Gemini 3 Pro. Claude Sonnet 4.6 demonstrates uniformly superior performance, GPT-5.2 shows intermediate performance, and Gemini 3 Pro exhibits greater variability across domains. Higher scores indicate better clinician-rated performance.

**Figure 2:**
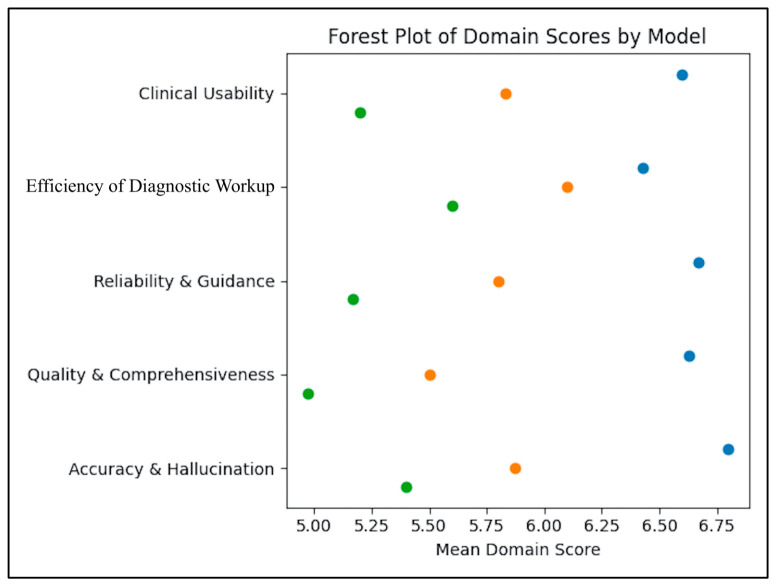
Forest plot showing mean domain scores across five clinical evaluation criteria. Blue dots represent Claude Sonnet 4.6, orange dots represent GPT-5.2, and green dots represent Gemini 3 Pro. Claude Sonnet 4.6 consistently achieved the highest scores across all domains, followed by GPT-5.2 and Gemini 3 Pro. Dots positioned farther to the right indicate higher mean clinician-rated performance.

Visual inspection of multidimensional performance profiles supported quantitative findings. Radar plot visualization demonstrated consistently superior domain-wide performance of Claude Sonnet 4.6, with more homogeneous scoring patterns compared with the greater variability observed for Gemini 3 Pro (Figure 1). Forest plot comparisons further illustrated the magnitude of inter-model differences across domains, highlighting the largest effect sizes in accuracy, comprehensiveness, reliability, and clinical usability, whereas efficiency of diagnostic workup showed smaller between-model separation (Figure 2).

**Clinical Usability and Practical Applicability:** Clinical usability differed markedly among models (H = 37.252, *p* < 0.001). Claude Sonnet 4.6 produced clearer, better organized, and more clinically actionable outputs.

All pairwise comparisons remained significant after Bonferroni correction:

- Claude Sonnet 4.6 vs. Gemini 3 Pro: U = 102, *p* < 0.001;
- Claude Sonnet 4.6 vs. GPT-5.2: U = 177, *p* < 0.001;
- GPT-5.2 vs. Gemini 3 Pro: U = 272, *p* = 0.005.

### 3.2. Inter-Rater Agreement

Inter-rater agreement for the composite score was moderate-to-good (ICC = 0.72, 95% CI 0.48–0.86; two-way mixed-effects, absolute agreement, average-measures; F(29,58) = 4.016, *p* < 0.001).

Although evaluator R.E. assigned slightly higher absolute scores (overall mean ≈6.2) compared with U.C. (≈5.7) and A.G.C. (≈5.9), all evaluators demonstrated identical model ranking patterns across all domains, confirming robust agreement in relative performance assessment. The scoring patterns were otherwise broadly consistent across the three evaluators. Importantly, all three raters maintained the same model ranking hierarchy (Claude Sonnet 4.6 > GPT-5.2 > Gemini 3 Pro) across all evaluation domains (Supplementary File S1).

This consistency supports the objectivity and reproducibility of the evaluation framework.

### 3.3. Case-Level Performance

Claude Sonnet 4.6 achieved the highest composite score in 9 of 10 clinical cases, demonstrating consistent superiority across diverse etiologies of secondary hypertension (Table 3).

The only exception was **Case 2 (Primary Hyperaldosteronism)**, where GPT-5.2 achieved the highest score. However, score dispersion among models was minimal, likely reflecting the structured and algorithm-driven nature of this diagnosis.

Largest performance gaps were observed in complex endocrine–metabolic cases:

**Cushing’s syndrome (Case 8): Δ = 2.46 (Claude vs. Gemini)**

**Diabetic nephropathy (Case 9): Δ = 2.20 (Claude vs. Gemini)**

These findings suggest increased vulnerability of some models when multistep hormonal interpretation and systemic metabolic reasoning are required.

In contrast, Claude Sonnet 4.6 demonstrated near-ceiling performance (≥6.87) in:

- Pheochromocytoma;
- Renal artery stenosis;
- White coat hypertension;
- Fibromuscular dysplasia.

This pattern indicates strong adaptability across vascular, endocrine, and mixed-etiology hypertension scenarios.

**Figure 3:**
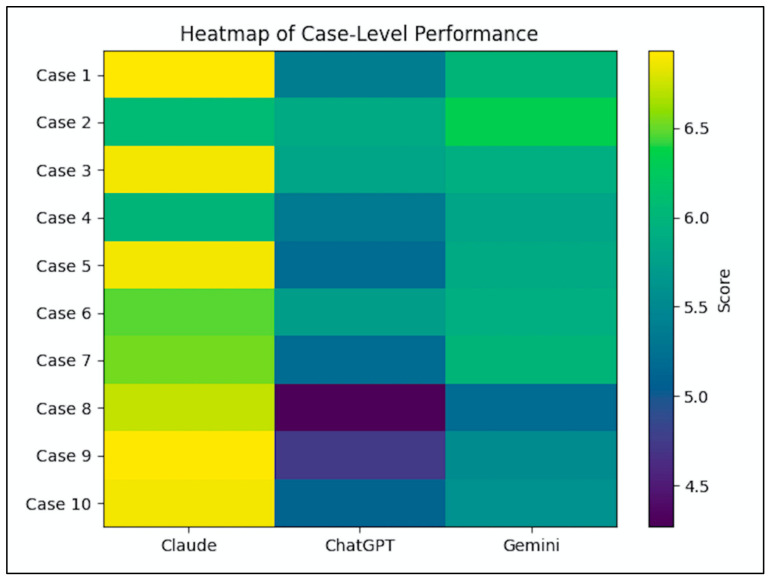
Heatmap depicting case-level composite scores across ten clinical scenarios. Warmer colors indicate higher performance. Claude Sonnet 4.6 ranked highest in nine of ten cases, with the largest inter-model performance gaps observed in complex endocrine disorders.

Descriptive analysis of vignette-specific composite scores demonstrated stable performance patterns across heterogeneous etiologies of secondary hypertension. Heatmap visualization revealed consistently high performance of Claude Sonnet 4.6 across nearly all clinical scenarios, whereas performance variability was more pronounced for GPT-5.2 and Gemini 3 Pro, particularly in complex endocrine-metabolic conditions (Figure 3). These graphical representations facilitated identification of scenario-specific strengths and weaknesses that were not fully captured by aggregated statistical comparisons.

A supplementary binary performance analysis was performed by dichotomizing the 7-point Likert scores using two predefined thresholds. First, scores ≥5 were accepted as present/adequate clinical performance, whereas scores <5 were accepted as absent/not adequate performance. Second, a stricter sensitivity threshold of ≥6 was used to define strong clinical performance. Using the ≥5 threshold, present/adequate performance based on composite scores was observed in 30 of 30 evaluations for Claude Sonnet 4.6 (100%), 30 of 30 evaluations for GPT-5.2 (100%), and 19 of 30 evaluations for Gemini 3 Pro (63.3%). When all five evaluation domains were analyzed together, present/adequate performance was observed in 149 of 150 domain-level scores for Claude Sonnet 4.6 (99.3%), 145 of 150 for GPT-5.2 (96.7%), and 107 of 150 for Gemini 3 Pro (71.3%). Using the stricter ≥6 threshold, strong clinical performance based on composite scores was observed in 28 of 30 evaluations for Claude Sonnet 4.6 (93.3%), 17 of 30 evaluations for GPT-5.2 (56.7%), and 11 of 30 evaluations for Gemini 3 Pro (36.7%). At the domain-score level, strong clinical performance was observed in 145 of 150 domain-level scores for Claude Sonnet 4.6 (96.7%), 108 of 150 for GPT-5.2 (72.0%), and 64 of 150 for Gemini 3 Pro (42.7%). Overall, both binary threshold analyses supported the primary Likert-scale findings. Claude Sonnet 4.6 showed the most consistent performance across composite and domain-level assessments. GPT-5.2 demonstrated generally adequate performance, although the proportion of strong-performance ratings was lower than that of Claude Sonnet 4.6. Gemini 3 Pro showed the lowest proportion of adequate and strong-performance ratings, indicating greater variability and lower clinical reliability across the evaluated scenarios (Supplementary File S2).

## 4. Discussion

In this blinded head-to-head comparative study, we evaluated three advanced commercially available large language models (LLMs) across clinically realistic secondary hypertension (SH) scenarios encompassing diagnostic reasoning, diagnostic workup, management planning, longitudinal follow-up, and patient education. Using expert-developed case vignettes and a structured, guideline-aligned evaluation framework, a clear performance gradient emerged. Claude Sonnet 4.6 received consistently higher expert ratings across most evaluation domains and ranked first in nine of ten clinical scenarios. GPT-5.2 showed intermediate performance, while Gemini 3 Pro exhibited greater variability across domains and clinical contexts. Interestingly, diagnostic workup efficiency scores were comparatively similar among models, suggesting convergence in broadly guideline-consistent diagnostic prioritization despite differences in overall reasoning depth and reliability. Collectively, these exploratory findings suggest clinically relevant inter-model variability and indicate that LLM performance in secondary hypertension remains strongly model-dependent.

Secondary hypertension represents a particularly demanding test environment for AI-assisted reasoning because accurate evaluation requires integration of multidisciplinary knowledge, probabilistic thinking, and guideline-based decision-making rather than simple factual recall [15]. Distinguishing primary from secondary etiologies, interpreting hormonal pathophysiology, accounting for medication interference, and sequencing confirmatory tests require nuanced multistep reasoning that challenges both trainees and experienced clinicians. Our findings suggest that advanced LLMs can approximate structured specialist reasoning when prompts clearly define professional roles and output structure, consistent with prior studies demonstrating improved diagnostic performance using structured prompting frameworks [9,16]. The superior performance of the highest-ranked model—particularly in guideline concordance—supports emerging evidence that newer LLM architectures may better internalize evidence-based clinical reasoning patterns [12,13,17].

The standardized prompt used in this study clearly framed the task as being performed by a board-certified specialist in Endocrinology and Hypertension and required a fixed five-domain response structure. Such role-based and structured prompting may encourage more guideline-oriented reasoning, improve organization, and reduce omission of clinically relevant steps. However, the impact of prompt engineering may not be uniform across model architectures, as proprietary models differ in alignment strategies, safety guardrails, and response-generation behavior. Therefore, the present results should be interpreted within the context of this standardized prompting strategy, and future studies should systematically compare alternative prompt designs, including neutral prompts, specialist-role prompts, and stepwise reasoning prompts.

Hallucinations remain a principal barrier to safe implementation of generative artificial intelligence in clinical medicine [18]. Performance differences in this study were most evident in hallucination control and reliability domains, where lower-performing models occasionally introduced unnecessary investigations, incomplete diagnostic hierarchies, or insufficient risk stratification strategies. This observation aligns with previous reports indicating that hallucinations are more likely to arise during complex, multistep clinical reasoning tasks rather than simple knowledge retrieval [19]. Importantly, even the highest-performing model identified in this study did not achieve uniformly optimal outputs across all cases, reinforcing the prevailing consensus that LLMs should currently function as clinical decision-support adjuncts under physician supervision rather than autonomous decision-makers [20,21,22]. From a real-world implementation perspective, this limitation is particularly important because inaccurate AI-generated recommendations may influence clinician judgment through automation bias, especially in high-volume clinical settings where time constraints may reduce opportunities for detailed verification. Accordingly, safe integration of LLMs into endocrine and hypertension practice will likely require layered safeguards, including clinician validation, transparent citation of evidence sources, and institutional oversight mechanisms.

A distinctive contribution of this study is the inclusion of diagnostic workup efficiency as an evaluation domain. Over-testing is a recognized challenge in SH evaluation, where indiscriminate imaging and excessive biochemical investigations increase healthcare costs and patient burden. The relatively modest differences observed between models suggest that economic reasoning remains inconsistently represented within LLM outputs, emphasizing the importance of evaluating artificial intelligence systems not only for diagnostic accuracy but also for health-system applicability and value-based care alignment. In practical clinical environments, AI systems that recommend extensive low-yield testing without adequate prioritization may paradoxically increase healthcare utilization and contribute to unnecessary referrals or diagnostic cascades. Therefore, future model development should incorporate stronger alignment with principles of cost-conscious and resource-aware clinical practice.

Across models, patient education responses were generally strong, consistent with previous findings that LLMs effectively translate complex medical concepts into accessible language [23]. This capability may represent one of the earliest safe clinical applications of LLMs, particularly for chronic endocrine conditions requiring sustained patient engagement and shared decision-making. Nevertheless, variability in nuance, contextual framing, and risk communication underscores the continued necessity of clinician oversight [22]. Importantly, patient-facing implementation also raises concerns regarding misinformation amplification, inappropriate reassurance, and unequal comprehension among individuals with varying levels of health literacy. Consequently, AI-generated educational materials should ideally be integrated into supervised clinical communication strategies rather than used as independent counseling tools.

The observed performance hierarchy indicates that LLM capability is rapidly evolving but remains model-dependent. Blinded anonymization minimized brand bias, demonstrating that perceived technological prominence does not necessarily predict clinical reasoning performance. To our knowledge, this is the first comparative chatbot evaluation specifically focused on secondary hypertension and assessed by experienced clinician-researchers using a structured, guideline-oriented framework. The supplementary binary analyses strengthen the interpretation of the primary findings by showing that the observed model hierarchy was not limited to median Likert-score comparisons. Rather, similar performance patterns were observed when scores were transformed into clinically interpretable adequate-performance and strong-performance categories. Together with case-level ranking and evaluator-level consistency analyses, these findings support the robustness of the observed performance gradient across models. Study strengths include expert-generated vignettes reflecting real clinical complexity, blinded model evaluation, structured scoring aligned with evidence-based practice, moderate-to-good inter-rater agreement, and incorporation of diagnostic workup efficiency as a novel clinical metric extending beyond accuracy-centered benchmarking. A key knowledge contribution of this study is the demonstration that general-purpose LLM performance is not uniformly transferable to secondary hypertension, a domain requiring multistep diagnostic reasoning, biochemical interpretation, medication-aware workup planning, and longitudinal management decisions. Thus, the clinical value of the study is not limited to ranking individual models, but lies in defining the current boundaries and limitations of accessible LLMs when applied to complex subspecialty reasoning.

An additional ethical and regulatory concern relates to the evaluation of proprietary commercial LLMs whose architectures, training datasets, alignment procedures, and update cycles are not fully transparent. Because these systems function as closed-box technologies, observed performance differences cannot be directly attributed to specific model design features, data sources, or safety mechanisms. This limits mechanistic interpretability, external reproducibility, and long-term comparability, particularly when commercial models are updated without public notification or version-level documentation. In clinical benchmarking, such opacity raises concerns regarding accountability, bias detection, data provenance, and regulatory oversight. Therefore, evaluations of proprietary LLMs should be interpreted as time-specific assessments of accessible commercial systems rather than definitive comparisons of underlying technologies. Future studies should report model versions, query dates, access conditions, and prompting parameters in detail, and regulatory frameworks should encourage greater transparency, post-deployment monitoring, auditability, and clear medico-legal responsibility before these tools are incorporated into clinical decision-support workflows. For this reason, the present findings should be viewed as a deployment-facing clinical evaluation of accessible commercial LLMs, rather than as a claim that these systems are specifically optimized for secondary hypertension or that their underlying technologies can be mechanistically compared.

The present study should also be interpreted within the paradigm of clinical rather than purely technical benchmarking. From a computer science perspective, evaluating proprietary, off-the-shelf LLMs may appear limited because their architectures, training data, alignment procedures, and update mechanisms are not externally controllable. However, from a clinical and translational perspective, this limitation is precisely what makes such evaluation necessary. General-purpose commercial LLMs are already accessible to clinicians, trainees, and patients and may be used for complex medical reasoning regardless of whether they were specifically fine-tuned for secondary hypertension or endocrine decision-making. Therefore, assessing their diagnostic accuracy, hallucination control, guideline concordance, workup efficiency, and clinical usability in a high-risk subspecialty domain represents an essential safety step before these tools can be responsibly considered in clinical workflows. In this context, application-specific evaluation generates clinically meaningful knowledge by defining the current boundaries of model performance, identifying scenario-specific vulnerabilities, and clarifying the level of physician oversight required for real-world use. Because proprietary LLMs function as closed-box systems, clinicians cannot infer that broad medical knowledge or general reasoning ability will safely transfer to multistep endocrine-hypertension scenarios involving biochemical interpretation, medication effects, confirmatory testing, and longitudinal management. Domain-specific, clinician-led validation of accessible systems therefore complements, rather than replaces, technical AI benchmarking and provides deployment-relevant evidence for safe and accountable clinical implementation.

Several limitations should be acknowledged. Case vignette designs cannot fully replicate real-world clinical uncertainty, comorbidity interactions, or longitudinal patient trajectories. Model outputs were assessed at a single time point despite rapid iterative updates in LLM architecture and training pipelines, and prompting strategies may influence performance outcomes [24]. The evaluation was limited to English-language prompts, which may restrict generalizability across healthcare systems and linguistic settings. The study design evaluated isolated prompt-response interactions and did not assess integration with electronic health records, laboratory information systems, or multidisciplinary clinical workflows that characterize routine endocrine practice. The practical performance of LLMs in real clinical environments may therefore differ substantially from simulation-based benchmarking conditions. Another important limitation is the relatively small number of clinical vignettes. Although 10 cases were designed to cover major etiologies of SH and to vary in diagnostic complexity, they represent only a limited sample of possible clinical presentations. Therefore, results may be sensitive to individual case design choices, and generalizability to broader real-world SH populations remains constrained. Future studies should include larger vignette sets, multicenter case development, and preferably prospective clinical data to improve external validity. Although all model outputs were anonymized using code names before expert evaluation, formal post-evaluation assessment of blinding integrity was not performed. Future benchmarking studies could incorporate a ‘guess-the-model’ survey after scoring to quantify whether evaluators could infer model identity and to assess the potential influence of perceived model recognition on ratings. In addition, although anonymization procedures were applied, formal post-evaluation assessment of blinding integrity was not conducted. Therefore, the possibility that evaluators may have inferred model identity based on stylistic characteristics cannot be completely excluded. Another important consideration relates to evaluator expertise. The present study intentionally employed senior endocrinologists and cardiologists because the primary objective was to assess clinical relevance, guideline concordance, diagnostic safety, and practical usability from the perspective of physicians managing SH in real-world practice. However, expertise in endocrine or cardiovascular medicine does not necessarily imply formal specialization in machine learning evaluation methodology, computational benchmarking, or AI validation science. Consequently, domains such as hallucination assessment, reliability evaluation, and benchmarking interpretation were evaluated primarily through their clinical manifestations and potential implications for patient care rather than through technical model-performance metrics commonly used in computer science research. Future interdisciplinary investigations incorporating experts in AI evaluation, biomedical informatics, computational benchmarking, and medical AI governance may provide additional methodological depth and facilitate integration of both clinical and technical evaluation frameworks.

Another important limitation relates to the stochastic and non-deterministic nature of LLM outputs. Each vignette in the present study was submitted once under standardized prompting conditions without repeated sampling, response regeneration, or iterative prompting. Consequently, the evaluated outputs may not fully represent the complete response distribution or stability characteristics of each model. Although this approach was intentionally selected to simulate pragmatic single-interaction clinical usage, response variability across repeated runs may influence reproducibility and comparative rankings. Because the original queries were performed in February 2026, a delayed post hoc reproducibility analysis during the revision stage was not considered methodologically appropriate. Proprietary LLMs may undergo model updates, platform changes, or undocumented system modifications over time; therefore, repeated queries several months later could reflect model drift rather than true within-model stochastic variability.

Future research should evaluate LLM performance in prospective clinical workflows, incorporate repeated-query methodologies, variance estimation, real patient data streams, and sensitivity testing across inference configurations to better characterize model consistency. Domain-specific fine-tuning using endocrine-focused datasets and guideline-informed reinforcement learning strategies may further improve safety and reduce hallucination frequency [25,26,27]. Development of benchmarking frameworks tailored to complex subspecialty conditions such as secondary hypertension will be essential as AI systems transition from experimental tools toward clinically integrated decision-support platforms. In addition, future investigations should explore clinician–AI interaction dynamics, including how physicians interpret, trust, accept, or override AI-generated recommendations during real-time clinical decision-making. Regulatory governance, medico-legal accountability, data privacy protection, and transparency of model reasoning processes will also become increasingly important as these systems move closer to implementation within healthcare infrastructures.

### Clinical Implications

The findings of this study suggest that contemporary large language models LLMs are approaching a level of performance that may support selected aspects of clinical decision-making in complex endocrine disorders such as secondary hypertension. Although none of the evaluated models demonstrated fully autonomous clinical reliability, higher-performing systems showed substantial capability in structured diagnostic reasoning, guideline-consistent investigation planning, and synthesis of management strategies. These characteristics indicate that LLMs may serve as supportive cognitive aids for clinicians, particularly in settings where access to subspecialty expertise is limited. Future domain-specific optimization of medical LLMs could incorporate reinforcement learning from expert clinician feedback in which guideline concordance, diagnostic workup efficiency, avoidance of unnecessary testing, and cost-conscious decision-making are included in detail as reward parameters. Such training approaches may help improve the practical clinical utility of LLMs beyond diagnostic accuracy alone.

One potential near-term application lies in decision-support augmentation. In secondary hypertension, appropriate evaluation requires careful sequencing of biochemical tests, medication adjustments, and imaging modalities. LLMs capable of summarizing guideline-based pathways may assist clinicians in verifying diagnostic algorithms, reducing omissions, and improving adherence to evidence-based workflows. This support may be particularly valuable for general practitioners, trainees, and physicians practicing in resource-constrained environments. However, successful real-world integration will depend on maintaining clear boundaries between supportive recommendation systems and autonomous clinical decision-making. AI-generated suggestions should remain transparent, reviewable, and easily challengeable by clinicians to avoid overreliance and preserve clinical accountability.

A second practical implication involves clinical documentation and information synthesis. The ability of LLMs to integrate laboratory data, imaging findings, and clinical histories into structured summaries may reduce cognitive burden and improve efficiency in multidisciplinary care. In complex cases requiring coordination among endocrinologists, cardiologists, nephrologists, and radiologists, structured AI-assisted summaries could facilitate communication and reduce diagnostic delays. Integration with electronic health record systems may further enhance this functionality, although interoperability, data security, and standardization challenges remain important barriers to implementation.

Third, the consistently strong performance observed in patient-oriented education highlights an area of relatively low clinical risk and high potential utility. Secondary hypertension often involves chronic disease monitoring and complex hormonal evaluations that patients may find difficult to understand. LLMs can translate technical medical information into accessible language, potentially improving patient engagement, treatment adherence, and shared decision-making processes. However, clinician oversight remains essential to ensure contextual accuracy and appropriate risk communication. Importantly, patient-facing systems should also be carefully designed to avoid dissemination of misleading or non-personalized recommendations that may conflict with individualized treatment plans.

The incorporation of diagnostic workup efficiency considerations in model evaluation further underscores the potential role of LLMs in promoting value-based care. By prioritizing high-yield diagnostic strategies and discouraging unnecessary testing, AI-assisted tools may contribute to more efficient resource utilization. Nevertheless, the modest differences observed among models suggest that economic reasoning remains insufficiently developed and should be strengthened through targeted training on health-system stewardship principles.

Importantly, these findings do not support replacement of clinician judgment. Instead, LLMs should currently be conceptualized as adjunctive tools that complement, rather than substitute, physician expertise. Human oversight remains indispensable for contextual interpretation, ethical decision-making, and individualized patient care. The most realistic near-term implementation model may involve hybrid clinician–AI workflows in which LLMs assist with information synthesis, differential diagnosis generation, and patient communication while final clinical responsibility remains entirely physician-directed.

So, the novelty of this study lies not in benchmarking per se, but in the structured clinical assessment of currently accessible LLMs in a high-risk, subspecialty diagnostic setting where inappropriate recommendations may lead to delayed diagnosis, unnecessary investigations, or unsafe management.

As LLM architectures continue to evolve, integration into electronic health systems, guideline databases, and specialty-specific knowledge repositories may further enhance their reliability. Establishing regulatory standards, validation frameworks, and medico-legal guidance will be essential before widespread clinical implementation. Equally important will be the development of continuous post-deployment monitoring systems capable of detecting unsafe outputs, performance drift, and unintended biases across diverse patient populations and healthcare settings.

## 5. Conclusions

In this pilot blinded comparative benchmarking study, large language models demonstrated heterogeneous and model-dependent performance profiles in the evaluation and management of secondary hypertension. The exploratory results indicated a clear clinician-rated performance hierarchy, with Claude Sonnet 4.6 showing the most consistent and highest overall scores, GPT-5.2 demonstrating intermediate capability, and Gemini 3 Pro exhibiting greater variability, particularly in complex endocrine–metabolic scenarios. Differences appeared most pronounced in domains requiring nuanced clinical reasoning, whereas diagnostic workup efficiency was relatively comparable across models. Although these findings support the potential role of LLMs as clinical decision-support adjuncts, they should be interpreted as hypothesis-generating rather than definitive evidence of model superiority. Larger vignette sets, repeated-query designs, multicenter expert evaluation, and prospective clinical validation are needed before routine clinical implementation.

**Key Points**

- This blinded clinician-led study compared three LLMs across 10 secondary hypertension vignettes.
- LLM performance was heterogeneous and model-dependent, with a clear clinician-rated hierarchy.
- Claude Sonnet 4.6 showed the most consistent performance, followed by GPT-5.2 and Gemini 3 Pro.
- Diagnostic workup efficiency was relatively comparable, suggesting shared ability to prioritize guideline-based testing.
- Hallucination risk and multistep reasoning variability support clinician oversight and larger validation studies.

**Take-Home Messages**

- LLMs may support, but not replace, specialist reasoning in secondary hypertension.
- Structured prompting can improve clinically organized, guideline-oriented AI responses.
- Safety concerns persist in complex endocrine–metabolic scenarios requiring multistep interpretation.
- Patient education and documentation support may represent near-term clinical applications.
- Larger multicenter, repeated-query, prospective studies are needed before routine clinical implementation.

## Figures and Tables

**Table 1:** Illustration of how each prompted task contributed to the multidimensional evaluation framework.

Prompted Response Component	Primary Evaluation Domains Influenced
Diagnosis & Differential Diagnosis	Accuracy, Comprehensiveness, Reliability
Diagnostic Workup Strategy	Accuracy, Efficiency, Reliability
Management Plan	Reliability, Usability, Accuracy
Follow-up Strategy	Reliability, Usability
Patient-Oriented Counseling	Usability, Comprehensiveness

**Table 2:** Criterion-Based Performance.

Criterion	Claude Sonnet 4.6	GPT-5.2	Gemini 3 Pro	H	*p* ^a^
Accuracy & Hallucination Control	**7.0 (7.0–7.0)**	6.0 (5.75–6.0)	5.5 (5.0–6.0)	42.443	<0.001
Quality & Comprehensiveness	**7.0 (6.0–7.0)**	6.0 (5.0–6.0)	5.0 (4.0–6.0)	39.075	<0.001
Reliability & Clinical Guidance	**7.0 (6.0–7.0)**	6.0 (5.0–6.0)	5.0 (4.0–6.0)	40.314	<0.001
Efficiency of Diagnostic Workup	**7.0 (6.0–7.0)**	6.0 (6.0–7.0)	6.0 (4.0–7.0)	9.148	0.010
Clinical Usability	**7.0 (6.0–7.0)**	6.0 (5.0–6.0)	5.0 (4.0–6.0)	37.252	<0.001
Composite score	**6.8 (6.20–7.00)**	6.0 (5.40–6.05)	5.2 (4.55–6.20)	40.055	<0.001

Bold values indicate the highest-scoring model for each criterion. ᵃ Kruskal–Wallis H test.

**Table 3:** Mean of raters’ composite scores per model across the 10 clinical vignettes.

Case	Diagnosis	Claude Sonnet 4.6	GPT-5.2	Gemini 3 Pro
1	Pheochromocytoma	**6.93**	6.00	5.40
2	Primary Hyperaldosteronism	6.07	**6.33**	5.87
3	Renal Artery Stenosis (Atherosclerotic)	**6.87**	5.93	5.80
4	Primary Hyperparathyroidism	**6.00**	5.80	5.33
5	White Coat/Early Essential Hypertension	**6.87**	5.87	5.20
6	Obstructive Sleep Apnea	**6.47**	5.93	5.73
7	Coarctation of the Aorta	**6.53**	6.00	5.20
8	Cushing Syndrome	**6.73**	5.20	4.27
9	Diabetic Nephropathy	**6.93**	5.53	4.73
10	Fibromuscular Dysplasia (Renal Artery)	**6.87**	5.60	5.13
	Overall Mean	**6.63**	5.82	5.27

Bold values indicate the highest-scoring model for each case.

## Data Availability

The anonymized dataset generated and analyzed during the current study is provided as Supplementary File S1. The file includes the blinded evaluator scores used for statistical analyses. No real patient data or identifiable personal information are included.

## Supplementary Materials

The following supporting information can be downloaded at: https://www.mdpi.com/article/10.3390/diagnostics16142165/s1, Supplementary Table S1. LLM code names and model correspondence used in the blinded evaluation. Supplementary File S1. The anonymized dataset generated and analyzed during the current study. Supplementary File S2. Standardized prompt template used for all LLM queries, characteristics of the clinical vi-gnettes included in the study, the details of the model access and reproducibility, and supplementary checklist analysis items.
