# Supplementary material for: Diagnostic Performance and Workup Efficiency of Large Language Models in Secondary Hypertension: A Blinded Comparative Study
Source: Diagnostics (Basel). 2026 Jul 10;16(14):2165. doi: 10.3390/diagnostics16142165 (PMC13409298; doi:10.3390/diagnostics16142165)
Supplement: Supplementary file 1 [file diagnostics-16-02165-s001.zip › Supplementary file S2/1. Direction.pdf]

CASE-1	Earth	Air	Water
Accuracy and Hallucination Control (1–7)			
Quality and Comprehensiveness of Clinical Reasoning (1–7)			
Reliability and Safety of Clinical Guidance (1–7)			
Efficiency of Diagnostic Workup (1–7)			
Clinical Usability and Practical Applicability (1–7)			
Mark Your Favorite Response			

CASE-2	Earth	Air	Water
Accuracy and Hallucination Control (1–7)			
Quality and Comprehensiveness of Clinical Reasoning (1–7)			
Reliability and Safety of Clinical Guidance (1–7)			
Efficiency of Diagnostic Workup (1–7)			
Clinical Usability and Practical Applicability (1–7)			
Mark Your Favorite Response			

CASE-3	Earth	Air	Water
Accuracy and Hallucination Control (1–7)			
Quality and Comprehensiveness of Clinical Reasoning (1–7)			
Reliability and Safety of Clinical Guidance (1–7)			
Efficiency of Diagnostic Workup (1–7)			
Clinical Usability and Practical Applicability (1–7)			
Mark Your Favorite Response			

CASE-4	Earth	Air	Water
Accuracy and Hallucination Control (1–7)			
Quality and Comprehensiveness of Clinical Reasoning (1–7)			
Reliability and Safety of Clinical Guidance (1–7)			
Efficiency of Diagnostic Workup (1–7)			
Clinical Usability and Practical Applicability (1–7)			
Mark Your Favorite Response			

CASE-5	Earth	Air	Water
Accuracy and Hallucination Control (1–7)			
Quality and Comprehensiveness of Clinical Reasoning (1–7)			
Reliability and Safety of Clinical Guidance (1–7)			
Efficiency of Diagnostic Workup (1–7)			
Clinical Usability and Practical Applicability (1–7)			
Mark Your Favorite Response			

CASE-6	Earth	Air	Water
Accuracy and Hallucination Control (1–7)			
Quality and Comprehensiveness of Clinical Reasoning (1–7)			
Reliability and Safety of Clinical Guidance (1–7)			
Efficiency of Diagnostic Workup (1–7)			
Clinical Usability and Practical Applicability (1–7)			
Mark Your Favorite Response			

CASE-7	Earth	Air	Water
Accuracy and Hallucination Control (1–7)			
Quality and Comprehensiveness of Clinical Reasoning (1–7)			
Reliability and Safety of Clinical Guidance (1–7)			
Efficiency of Diagnostic Workup (1–7)			
Clinical Usability and Practical Applicability (1–7)			
Mark Your Favorite Response			

CASE-8	Earth	Air	Water
Accuracy and Hallucination Control (1–7)			
Quality and Comprehensiveness of Clinical Reasoning (1–7)			
Reliability and Safety of Clinical Guidance (1–7)			
Efficiency of Diagnostic Workup (1–7)			
Clinical Usability and Practical Applicability (1–7)			
Mark Your Favorite Response			

CASE-9	Earth	Air	Water
Accuracy and Hallucination Control (1–7)			
Quality and Comprehensiveness of Clinical Reasoning (1–7)			
Reliability and Safety of Clinical Guidance (1–7)			
Efficiency of Diagnostic Workup (1–7)			
Clinical Usability and Practical Applicability (1–7)			
Mark Your Favorite Response			

CASE-10	Earth	Air	Water
Accuracy and Hallucination Control (1–7)			
Quality and Comprehensiveness of Clinical Reasoning (1–7)			
Reliability and Safety of Clinical Guidance (1–7)			
Efficiency of Diagnostic Workup (1–7)			
Clinical Usability and Practical Applicability (1–7)			
Mark Your Favorite Response			
