# Supplementary material for: Diagnostic Performance and Workup Efficiency of Large Language Models in Secondary Hypertension: A Blinded Comparative Study
Source: Diagnostics (Basel). 2026 Jul 10;16(14):2165. doi: 10.3390/diagnostics16142165 (PMC13409298; doi:10.3390/diagnostics16142165)
Supplement: Supplementary file 1 [file diagnostics-16-02165-s001.zip › Supplementary file S2/2. Evaluation Criteria.pdf]

Evaluation Criteria

1. Hallucination Tendency and Accuracy of Information (7-point Likert Scale)

Please evaluate the accuracy of the response in light of current literature and clinical guidelines:

- 7 – Completely Accurate: The response is entirely accurate and contains no misinformation or fabricated data.
- 4 – Partially Accurate: Accurate and inaccurate information are present at similar levels.
- 1 – Completely Incorrect: The response is entirely inaccurate and inconsistent with medical facts.

2. Response Quality and Comprehensiveness (7-point Likert Scale)

Please evaluate the depth and comprehensiveness of the response:

- 7 – Very High Quality: Excellent; covers all critical points in the management of secondary hypertension.
- 4 – Moderate Quality: Satisfactory but should be improved; some critical points are missing.
- 1 – Very Low Quality: Completely insufficient and lacks clinical reasoning.

3. Reliability and Clinical Guidance (7-point Likert Scale)

Please evaluate how appropriately the response guides the physician/patient through the diagnostic process:

- 7 – Fully Reliable: Guides the physician completely accurately and safely.
- 4 – Neutral: Neither appropriately nor inappropriately guides the physician.
- 1 – Completely Misleading: Entirely misleading and potentially clinically harmful.

4. Unnecessary Test Cost and Efficiency (7-point Likert Scale)

Please evaluate the cost-effectiveness of the investigations suggested by the model:

- 7 – Highly Efficient: Recommends only necessary tests without creating unnecessary costs.
- 4 – Moderately Efficient: Suggests some unnecessary tests but maintains the main diagnostic pathway.
- 1 – Highly Costly/Wasteful: Recommends many expensive tests without clinical indication.

5. Clinical Usability (7-point Likert Scale)

Please evaluate the practical applicability of the recommendations:

- 7 – Completely Usable: Recommendations are fully practical and useful.
- 1 – Completely Unusable: Recommendations are entirely impractical in real-life settings.
