# Supplementary material for: Diagnostic Performance and Workup Efficiency of Large Language Models in Secondary Hypertension: A Blinded Comparative Study
Source: Diagnostics (Basel). 2026 Jul 10;16(14):2165. doi: 10.3390/diagnostics16142165 (PMC13409298; doi:10.3390/diagnostics16142165)
Supplement: Supplementary file 1 [file diagnostics-16-02165-s001.zip › Supplementary file S2/3. Clinical Case Vignettes.pdf]

PROMT

Act as a board-certified specialist in Endocrinology and Hypertension. Your goal is to evaluate the following clinical case vignette according to the latest international guidelines (e.g., ESH/ESC Hypertension Guidelines, Endocrine Society Clinical Practice Guidelines).

Please provide your response strictly using the following 5-point structured format:

Diagnosis & Differential Diagnosis: Provide the most likely diagnosis and list relevant differential diagnoses with brief pathophysiological justifications.

Diagnostic Workup: Outline the most appropriate initial screening test and the 'gold standard' confirmatory test (if applicable). Prioritize cost-effectiveness and avoid ordering unnecessary or high-cost diagnostic tools unless clinically indicated.

Management Plan: State the primary treatment strategies, including medical, surgical, or lifestyle interventions in order of priority.

Long-term Follow-up: Provide a specific protocol for long-term monitoring, including frequency and necessary diagnostic tools.

Patient Education: Address the specific question asked by the patient at the end of the vignette. Use empathetic, clear, and patient-friendly language while maintaining medical accuracy.

If you are ready, I will send the first case scenario.

1- A 34-year-old woman is brought to the emergency department with a pounding headache, drenching sweats, and palpitations that began suddenly 30 minutes ago. She appearing extremely anxious and tremulous. Her blood pressure is 210/115 mmHg and her heart rate is 125 bpm. She mentions this has happened three times in the last month, often after exercise. A physical exam reveals no murmurs or abdominal bruits, but she is noticeably orthostatic when sitting up.

   A- What is the most likely diagnosis?

   B- What is the most sensitive initial screening test for this patient?

   C- The patient is scheduled for surgical resection. Which medication must be started first to prevent a hypertensive crisis during induction?

   D- What is the recommended protocol for long-term follow-up, including the duration and the specific diagnostic tools required?

   E- If a patient asks about "Will I be off blood pressure meds forever after surgery?" what do you reply?

2- A 45-year-old man presents for a follow-up regarding resistant hypertension. He is currently taking Lisinopril, Amlodipine, and Chlorthalidone, yet his BP remains 155/98 mmHg. He complains of occasional muscle weakness and fatigue. Routine labs show a serum potassium of 3.1 mEq/L (Normal: 3.5–5.0) despite not being on a loop diuretic. His plasma aldosterone concentration (PAC) is significantly elevated, and his plasma renin activity (PRA) is suppressed.

A- What is the most appropriate dignosis?

B- What is the "Gold Standard" test to differentiate between a unilateral adenoma and bilateral adrenal hyperplasia before surgery?

C- If the patient has bilateral adrenal hyperplasia, what is the first-line medical therapy?

D- What is the recommended protocol for long-term follow-up?

E- If a male patient presents with gynecomastia, what will you offer?

3-A 62-year-old male with a history of heavy smoking and peripheral vascular disease is started on an ACE inhibitor for new-onset hypertension (170/105 mmHg). Two weeks later, his serum creatinine has jumped from 1.1 mg/dL to 1.8 mg/dL. On auscultation of the abdomen, a faint high-pitched systolic-diastolic blowing sound is heard in the left upper quadrant.

A- What is the most likely diagnosis?

B- What is the initial non-invasive screening tool of choice?

C- Besides aggressive statin and antiplatelet therapy, what is the definitive management for refractory cases?

D- What is the recommended protocol for long-term follow-up?

E- If the patient asks about "Why did the blood pressure medicine make my kidney function worse?" what would you reply?

4- A 50-year-old woman presents with persistent hypertension and a history of recurrent calcium oxalate kidney stones. She complains of chronic constipation and a "foggy" mood. Her blood pressure is 150/95 mmHg. Laboratory results reveal a serum calcium of 11.2 mg/dL (Normal: 8.5–10.5) and a suppressed serum phosphorus.

A- What is the most likely diagnosis?

B- Which lab combination confirms the diagnosis?

C- What is the only curative treatment for this patient?

D- What is the recommended protocol for long-term follow-up?

E- The patient asks, Will my 'brain fog' and mood go away after the surgery?" what would you reply?

5- A 19-year-old male athlete presents for a sports physical. He is asymptomatic, but his blood pressure is consistently 145/92 mmHg. He has a BMI of 24, does not smoke, and has no family history of early cardiovascular disease. His physical exam, including femoral pulses and fundoscopy, is entirely normal. Extensive workup (including renal ultrasound, thyroid studies, and metanephrines) returns negative.

A- What is the most likely diagnosis?

B- Before lifelong medication, what should be done to rule out "White Coat Hypertension" in a young athlete?

C- What is the first-line intervention for a young patient with Stage 1 Essential Hypertension and no end-organ damage?

D- What is the recommended protocol for long-term follow-up?

E- The patient asks, "I'm an athlete and I eat well. Why is my BP high?" what would you reply?

6- A 52-year-old male with a BMI of 34 presents for a physical. His wife complains he "stops breathing" at night and snores loudly. He admits to severe daytime sleepiness and morning headaches. His BP is 158/96 mmHg, which is highest in the early morning.

A- What is the most likely cause of his hypertension?

B- What is the "Gold Standard" test to confirm this diagnosis?

C- What is the first-line treatment that can also help lower his blood pressure?

D- What is the recommended protocol for long-term follow-up?

E- The patient asks, Do I really have to wear this mask every night?" what would you reply?

7-An 18-year-old male presents with leg fatigue while playing soccer. On physical exam, his BP in the right arm is 165/90 mmHg, while his BP in the left leg is 110/70 mmHg. You note a continuous murmur heard best over the back, between the scapulae.

A- What is the most likely diagnosis?

B- What is the initial imaging test of choice, and what classic sign might be seen on a chest X-ray?

C- What is the definitive management for this patient?

D- What is the recommended protocol for long-term follow-up?

E- The patient asks, "Is my heart fixed now that the narrowing is gone?" what would you reply?

8-A 38-year-old woman presents with rapid weight gain, specifically in her face and abdomen. She has developed purple streaks (striae) on her stomach and complains of easy bruising. Her BP is 160/100 mmHg. She has no history of exogenous steroid use.

A-What is the most likely diagnosis?

B- Which test is an appropriate initial screening test?

C-If no tumor can not be found, what are the medical treatment of choices?

D- What is the recommended protocol for long-term follow-up?

E- The patient asks, Will these stretch marks and the 'moon face' ever go away?" what would you reply?

9- A 28-year-old male with a history of Type 1 Diabetes presents with a BP of 155/95 mmHg. He notices his urine looks "foamy." A dipstick reveals 3+ protein. His serum creatinine is slightly elevated at 1.4 mg/dL.

A- What is the most likely cause of his secondary hypertension?

B- What is the most important calculation to quantify the severity of this patient's kidney disease?

C- Which class of antihypertensive is specifically indicated here?

D- What is the recommended protocol for long-term follow-up?

E- The patient asks, Does protein in my urine mean my kidneys are failing?" ?" what would you reply?

10- A 28-year-old female marathon runner presents with a persistent, pulsatile "whooshing" sound in her ears (tinnitus) and occasional neck pain. Her blood pressure is 165/105 mmHg. She has no family history of hypertension and a BMI of 21. On physical exam, you hear a faint bruit over the right carotid artery and a separate, high-pitched epigastric bruit. Her serum creatinine is 0.7 mg/dL (Normal: 0.6–1.1).

A- What is the most likely underlying pathology?

B- Why is a Renal Artery Duplex Ultrasound sometimes a "trap" in diagnosis.

C- What is the preferred treatment for this patient?

D- What is the recommended protocol for long-term follow-up?

E- E-The patient asks "I heard I shouldn't do high-impact sports or neck adjustments?" ?" what would you reply?
