# Supplementary material for: Diagnostic Performance and Workup Efficiency of Large Language Models in Secondary Hypertension: A Blinded Comparative Study
Source: Diagnostics (Basel). 2026 Jul 10;16(14):2165. doi: 10.3390/diagnostics16142165 (PMC13409298; doi:10.3390/diagnostics16142165)
Supplement: Supplementary file 1 [file diagnostics-16-02165-s001.zip › Supplementary file S2/4. LLM_Air.pdf]

CASE-1

Diagnosis & Differential Diagnosis

- **Most Likely Diagnosis: Pheochromocytoma.**

The "classic triad" of paroxysmal pounding headache, drenching diaphoresis (sweating), and palpitations, combined with severe hypertension and tachycardia, is highly suggestive. The orthostatic hypotension (despite high supine BP) is a classic sign due to chronic volume depletion and impaired sympathetic reflexes caused by catecholamine excess.

- **Differential Diagnoses:**
  - **Panic Disorder/Anxiety Attack:** Often mimics the "paroxysmal" nature but usually lacks the severe hypertensive urgency and orthostatic changes seen here.
  - **Hyperthyroidism/Thyrotoxicosis:** Can cause palpitations, sweating, and tachycardia, but the blood pressure elevation is typically systolic, and the "attacks" are less episodic.
  - **Renovascular Hypertension:** Considered due to the severe BP, but the absence of abdominal bruits and the presence of the symptomatic triad make it less likely than pheochromocytoma.

Diagnostic Workup

- **Initial Screening Test: Plasma free metanephrines** (high sensitivity, especially for high-risk symptomatic patients) or **24-hour urinary fractionated metanephrines**. Given her current symptomatic state, plasma metanephrines are the most sensitive tool to rule out the condition.
- **Confirmatory "Gold Standard":** If biochemical tests are significantly elevated (usually >3 times the upper limit of normal), the next step is **Anatomical Imaging** (CT or MRI of the abdomen and pelvis) to localize the tumor.

Note: Imaging should only be performed after biochemical confirmation to avoid "incidentaloma" confusion.

Management Plan

1. **Alpha-Adrenergic Blockade (Primary Priority):** Phenoxybenzamine (irreversible) or Doxazosin/Terazosin (selective) must be started first for 7–14 days to expand intravascular volume and control BP.
2. **Beta-Adrenergic Blockade:** Only **after** adequate alpha-blockade is established (usually 2–3 days later). Starting a beta-blocker first can lead to "unopposed alpha-stimulation," causing a paradoxical and potentially fatal hypertensive crisis.
3. **High-Sodium Diet & Fluid Intake:** To reverse catecholamine-induced volume depletion and prevent post-operative hypotension.
4. **Surgical Intervention:** Minimally invasive (laparoscopic or robotic) adrenalectomy is the definitive treatment once the patient is hemodynamically stable.

Long-term Follow-up

- **Immediate Post-Op:** Plasma or urinary metanephrines should be measured **2–4 weeks** after surgery to ensure complete resection.
- **Standard Protocol:** Annual clinical assessment and biochemical testing (metanephrines) for at least **10 years**.
- **High-Risk Protocol:** For patients with genetic mutations (e.g., *SDHB*), large tumors, or paragangliomas, **lifelong annual surveillance** is recommended due to the risk of late recurrence or malignancy.

Patient Education

"It is very common to hope that surgery will be a 'total cure,' and for many patients, it is! However, it's important to understand that while removing the tumor usually stops the dangerous 'attacks' and significantly lowers your blood pressure, about 25% to 30% of patients may still have some degree of high blood pressure afterward. This can happen because long-term high blood pressure sometimes affects the blood vessels or kidneys.

Our goal is to get you off as many medications as possible, but we will monitor you closely to see if your body adjusts. Even if you feel perfect, we will need to check your labs once a year to make sure the 'factory' that makes those stress hormones hasn't started up somewhere else. You're through the hardest part, and we'll be with you for every step of the recovery."

CASE-2

Diagnosis & Differential Diagnosis

- **Most Likely Diagnosis: Primary Aldosteronism (Conn's Syndrome).**

The clinical presentation of resistant hypertension despite a three-drug regimen (including a diuretic), spontaneous hypokalemia, and the biochemical profile of elevated PAC with suppressed PRA (high Aldosterone-to-Renin Ratio) is the classic hallmark of this condition.

- **Differential Diagnoses:**
  - **Liddle Syndrome:** A genetic disorder causing hypertension and hypokalemia, but both aldosterone and renin would be suppressed.
  - **Apparent Mineralocorticoid Excess (AME):** Often due to licorice ingestion or genetic enzyme deficiency; also presents with low renin and low aldosterone.
  - **Renovascular Hypertension:** Would present with high blood pressure and high aldosterone, but **high** renin (secondary aldosteronism).

Diagnostic Workup

- **Initial Screening Test: Aldosterone-to-Renin Ratio (ARR)**. A ratio typically >20–30 (depending on units) with a PAC >15 ng/dL is highly suggestive.

- **Confirmatory Test: Oral Salt Loading Test** or **Saline Infusion Test**. Failure to suppress aldosterone levels below 5–10 ng/dL after salt loading confirms the diagnosis.
- **"Gold Standard" for Differentiation: Adrenal Venous Sampling (AVS)**. While a CT scan can show an adrenal mass, it often misses microadenomas or incorrectly identifies "incidentalomas." AVS is the only definitive way to distinguish between a unilateral aldosterone-producing adenoma (surgical) and bilateral adrenal hyperplasia (medical) by comparing hormone levels directly from the adrenal veins.

Management Plan

1. **Primary Strategy for Bilateral Hyperplasia: Mineralocorticoid Receptor Antagonists (MRAs)**. This is the first-line medical therapy.
2. **Specific Medication: Spironolactone** is the traditional choice, though it has side effects. **Eplerenone** is a more selective alternative if the patient cannot tolerate spironolactone.
3. **Lifestyle Intervention: Strict Sodium Restriction (<100 mmol/day)**. Reducing salt intake is vital to help the medication work effectively and lower blood pressure.
4. **Surgical Intervention:** Only if AVS confirms **unilateral** disease (adrenalectomy). For bilateral disease, surgery is generally not recommended.

Long-term Follow-up

- **Blood Pressure & Electrolyte Monitoring:** Check serum potassium and creatinine within **1–2 weeks** of starting or adjusting MRA therapy, then every 3–6 months once stable.
- **Annual Screenings:** Monitor for complications of long-term aldosterone excess, such as left ventricular hypertrophy (via ECG or Echo) and albuminuria (urine ACR), as these patients have a higher cardiovascular risk than those with essential hypertension.
- **Medication Titration:** Adjust doses to maintain BP <130/80 mmHg and potassium in the mid-normal range (approx. 4.5 mEq/L) to suppress the harmful effects of excess aldosterone.

Patient Education

"It sounds like you are experiencing a side effect that can happen with Spironolactone. Because that medication blocks certain hormones, it can sometimes mimic estrogen's effects in men, leading to breast tenderness or growth (gynecomastia).

The good news is that we have an alternative called **Eplerenone**. It is much more selective, meaning it still treats your blood pressure and protects your heart just as well, but it doesn't usually cause these hormonal side effects. If you're okay with it, I would like to switch your prescription today. The tenderness should gradually go away once we make the change."

CASE-3

Diagnosis & Differential Diagnosis

- **Most Likely Diagnosis: Renovascular Hypertension due to Atherosclerotic Renal Artery Stenosis (ARAS).**

The patient's age, smoking history, and peripheral vascular disease (PVD) strongly point toward atherosclerosis. The "flash" rise in creatinine (greater than 30%) after starting an ACE inhibitor is a pathognomonic clinical clue for bilateral renal artery stenosis or stenosis in a solitary functioning kidney. The systolic-diastolic abdominal bruit further localizes the pathology.

- **Differential Diagnoses:**
  - **Fibromuscular Dysplasia (FMD):** Also causes renal artery stenosis but typically affects younger women (ages 15–50) and has a "string of beads" appearance rather than atherosclerotic plaques.
  - **Hypertensive Nephrosclerosis:** Chronic damage from long-standing hypertension, but it would not explain the acute creatinine spike specifically following ACE inhibitor initiation.
  - **Abdominal Aortic Aneurysm (AAA):** Can present with an abdominal bruit, but does not typically cause the acute renal functional decline seen here.

Diagnostic Workup

- **Initial Screening Test: Duplex Ultrasonography of the Renal Arteries**. It is cost-effective, non-invasive, and avoids contrast (important given his rising creatinine). It evaluates both the anatomy and the blood flow velocity.
- **"Gold Standard" Confirmatory Test: Digital Subtraction Angiography (DSA)**. While CT or MR Angiography are often used, DSA remains the gold standard for definitive diagnosis and allows for simultaneous intervention if necessary. However, it is usually reserved for patients where revascularization is planned.

Management Plan

1. **Medical Management (Primary Priority):** Aggressive cardiovascular risk reduction is the cornerstone. This includes high-intensity **statins**, **antiplatelet therapy** (Aspirin), and smoking cessation.
2. **Blood Pressure Control:** Switching from an ACE inhibitor to other agents (such as Calcium Channel Blockers or Beta-blockers) to stabilize renal function.
3. **Definitive Management (Refractory Cases): Percutaneous Renal Angioplasty and Stenting**. This is indicated if blood pressure remains uncontrolled on maximal medical therapy, if there is a progressive decline in renal function, or in cases of recurrent "flash" pulmonary edema.
4. **Surgical Revascularization:** Reserved for complex cases where stenting is not technically feasible or has failed.

Long-term Follow-up

- **Renal Function Monitoring:** Serum creatinine and estimated GFR (eGFR) should be checked every **3 to 6 months**.
- **Imaging Surveillance:** Repeat Renal Duplex Ultrasound every **6 to 12 months** to monitor for restenosis (if a stent was placed) or progression of the disease in the opposite kidney.
- **Cardiovascular Screening:** Regular monitoring of other vascular beds (carotid and peripheral) due to the high systemic atherosclerotic burden in these patients.

Patient Education

"I understand it’s frustrating that a medicine meant to help your blood pressure seemed to hurt your kidneys. To explain it simply: your kidneys have 'sensors' that help them maintain blood flow. Because the arteries leading to your kidneys have become narrowed—similar to how your other leg arteries have—the kidneys were already working hard to keep pressure up inside themselves.

The ACE inhibitor is a great drug, but it 'relaxed' a specific valve in the kidney that was helping maintain that internal pressure. When that valve relaxed, the pressure inside the kidney dropped too much, causing the temporary rise in your creatinine levels. We caught this early, and by adjusting your medications and focusing on clearing those pathways, we can protect your kidney function moving forward."

CASE-4

Diagnosis & Differential Diagnosis

- **Most Likely Diagnosis: Primary Hyperparathyroidism (PHPT).**

The patient presents with the classic "stones, groans, and psychic overtones": recurrent calcium oxalate stones, chronic constipation ("groans"), and "foggy" mood ("psychic overtones"). The combination of hypercalcemia, suppressed phosphorus, and hypertension is highly characteristic of excess parathyroid hormone (PTH) secretion.

- **Differential Diagnoses:**
  - **Malignancy-Associated Hypercalcemia:** Usually presents with much higher calcium levels and a more acute onset, but PTH would be suppressed.
  - **Thiazide Diuretic Use:** Can cause mild hypercalcemia, but this patient's history of recurrent stones and low phosphorus points toward an endogenous PTH excess.
  - **Familial Hypocalciuric Hypercalcemia (FHH):** A genetic condition causing high calcium, but usually asymptomatic and does not cause kidney stones.

Diagnostic Workup

- **Initial Screening & Confirmation: Serum Calcium + Intact PTH (iPTH)**. The diagnosis is confirmed by finding elevated (or inappropriately "normal") PTH levels in the setting of hypercalcemia.
- **Secondary Workup: 24-hour Urinary Calcium**. This is essential to differentiate PHPT from FHH and to assess the risk of further kidney stone formation.
- **Localization (Pre-Surgical): Sestamibi Scan** or **Neck Ultrasound** to identify which of the four parathyroid glands is enlarged (adenoma) vs. multi-gland hyperplasia.

Management Plan

1. **Definitive Treatment: Surgical Parathyroidectomy**. This is the only curative intervention for primary hyperparathyroidism.
2. **Hydration:** Maintaining high fluid intake to reduce the risk of further kidney stones while awaiting surgery.
3. **Medical Management (If surgery is declined/delayed): Cinacalcet** (a calcimimetic) can lower calcium levels, and **Bisphosphonates** can protect bone density, though neither "cures" the underlying overactive gland.
4. **Avoidance:** Avoid thiazide diuretics and excessive Vitamin D/Calcium supplementation until the gland is removed.

Long-term Follow-up

- **Immediate Post-Op:** Check serum calcium and PTH within **24–48 hours** to confirm success (looking for a >50% drop in PTH) and monitor for "hungry bone syndrome" (hypocalcemia).
- **Bone Health:** A **DEXA Scan** (bone density) should be performed at baseline and repeated 1–2 years after surgery to monitor the expected recovery of bone mass.
- **Renal Monitoring:** Follow-up kidney imaging (ultrasound) if the patient has persistent symptoms of stones or reduced kidney function.
- **Annual Checks:** Yearly serum calcium and creatinine for at least 5 years to ensure there is no recurrence.

Patient Education

"It is very likely that you will feel a significant difference! Many patients find that the 'brain fog,' fatigue, and low mood improve remarkably—often within just a few days or weeks after the overactive gland is removed.

High calcium levels act almost like a 'dimmer switch' on your nervous system, slowing down your digestion (causing the constipation) and affecting your cognitive speed. While every recovery is unique, resolving the chemical imbalance in your blood is the most effective way to clear that fog and help your mood return to its baseline. We'll be monitoring your levels closely to make sure your body finds its new, healthy balance."

CASE-5

Diagnosis & Differential Diagnosis

- **Most Likely Diagnosis: Primary (Essential) Hypertension.**

Despite his young age and athletic lifestyle, the absence of secondary causes (normal renal imaging, thyroid, and catecholamines) and a normal physical exam (ruling out Coarctation of the Aorta via normal femoral pulses) points toward Essential Hypertension. In young, fit males, this is often characterized by high sympathetic drive or "Hyperdynamic Circulation."

- **Differential Diagnoses:**
  - **White Coat Hypertension:** Extremely common in clinical settings, where anxiety about the exam artificially inflates readings.
  - **Substance-Induced Hypertension:** Use of performance-enhancing drugs (PEDs), high-caffeine pre-workout supplements, or NSAIDs for sports injuries must be ruled out.
  - **Obstructive Sleep Apnea (OSA):** Even in athletes, large neck circumference or nasal obstruction can lead to OSA, though less likely given his normal BMI.

Diagnostic Workup

- **Initial Screening Tool: Ambulatory Blood Pressure Monitoring (ABPM)**. This is the gold standard for ruling out "White Coat Hypertension." It records BP over 24 hours during normal activities and sleep. Alternatively, **Home Blood Pressure Monitoring (HBPM)** for 7 days can be used.
- **Target Organ Damage Screen:** Even with negative initial tests, a **12-lead ECG** (to check for Left Ventricular Hypertrophy) and a **Urine Albumin-to-Creatinine Ratio (ACR)** are indicated to determine if the "Stage 1" hypertension is already affecting his heart or kidneys.

Management Plan

1. **Lifestyle Modification (First-Line):** For Stage 1 hypertension (140-159/90-99 mmHg) without end-organ damage, a **3–6 month trial** of lifestyle changes is recommended. This includes the DASH diet (high potassium/magnesium) and reducing sodium intake to <2,300 mg/day.
2. **Review of Supplements:** Immediate cessation of any stimulants, high-caffeine pre-workouts, or anabolic substances.
3. **Medical Therapy:** If BP remains elevated after lifestyle trials, an **ACE inhibitor** or **Angiotensin Receptor Blocker (ARB)** is typically preferred in young patients, as they do not impair exercise performance as much as beta-blockers.

Long-term Follow-up

- **Frequency:** Every **3 to 6 months** to monitor blood pressure stability and adherence to lifestyle changes.
- **Annual Assessment:** Yearly screening for metabolic changes (fasting glucose/lipid profile) and renal function (creatinine/ACR).
- **Re-evaluation:** If he remains controlled for a long period and successfully modifies his diet/supplements, a "step-down" trial to see if medication can be reduced or stopped may be considered under strict supervision.

Patient Education

"It's a great question, and I can see how frustrating it is when you're doing everything 'right' for your fitness. Hypertension isn't always about being out of shape; for some young athletes, it's simply down to genetics—how your body is 'wired' to manage salt or how active your natural adrenaline system is.

Think of it like the idle on a high-performance car engine: sometimes it's just set a little too high. Also, we should look closely at any 'sports' supplements, pre-workouts, or even frequent use of ibuprofen, as these can sneakily raise blood pressure. Our goal isn't to slow you down, but to make sure your heart isn't working harder than it needs to while you're at rest."

CASE-6

Diagnosis & Differential Diagnosis

- **Most Likely Diagnosis: Obstructive Sleep Apnea (OSA) with Secondary Hypertension.**

The patient's BMI of 34 (Class 1 Obesity), loud snoring, witnessed apneas, and morning symptoms (headaches/sleepiness) are hallmark signs. In OSA, repetitive collapses of the upper airway cause intermittent hypoxia and "micro-arousals," triggering a massive sympathetic nervous system surge. This leads to high blood pressure, typically characterized by a **"non-dipping" pattern** where BP fails to drop—or actually rises—during the night and early morning.

- **Differential Diagnoses:**
    - **Primary Hyperaldosteronism:** Often co-exists with OSA; however, the clear history of breathing pauses points to OSA as the primary driver.
    - **Obesity Hypoventilation Syndrome:** Similar to OSA but involves chronic daytime CO_2 retention; usually seen in patients with a BMI >30.
    - **Hypothyroidism:** Can cause weight gain, fatigue, and macroglossia (enlarged tongue) leading to snoring and hypertension.

Diagnostic Workup

- **Initial Screening: STOP-Bang Questionnaire** or **Epworth Sleepiness Scale** to quantify clinical risk.

- **"Gold Standard" Confirmatory Test: In-laboratory nocturnal Polysomnography (PSG)**.

This test measures the Apnea-Hypopnea Index (AHI). An AHI ≥15 (or ≥5 with symptoms) confirms the diagnosis. While home sleep tests are available, in-lab PSG is superior for detecting specific sleep stages and arousals associated with hypertensive surges.

Management Plan

1. **Continuous Positive Airway Pressure (CPAP):** This is the first-line and most effective treatment. It acts as a "pneumatic splint" to keep the airway open, reducing sympathetic overactivity and lowering blood pressure.
2. **Weight Loss & Lifestyle Intervention:** Aim for a 10% weight reduction through a structured diet and exercise program, which can significantly reduce the severity of OSA.
3. **Positional Therapy:** Avoiding the supine (back) sleeping position if the apneas are primarily gravity-dependent.
4. **Medical Management:** If BP remains high despite CPAP, **Mineralocorticoid Receptor Antagonists** (like Spironolactone) have shown particular efficacy in patients with both OSA and resistant hypertension.

Long-term Follow-up

- **CPAP Adherence Monitoring:** Review machine-recorded data (usage hours, leak rate, and residual AHI) after **30 to 90 days**, then annually.
- **Blood Pressure Surveillance:** Home BP monitoring should be performed specifically in the morning to ensure the "early morning surge" is resolving with treatment.
- **Annual Metabolic Review:** Monitoring weight, fasting glucose, and lipid profiles, as OSA is a major risk factor for cardiovascular disease and Type 2 Diabetes.

Patient Education

"I understand that the idea of wearing a mask can feel daunting and intrusive at first. But to answer your question: yes, for the treatment to protect your heart and brain, it needs to be used every night.

Think of the mask like a 'blood pressure pill' that you wear instead of swallow. When you skip a night, your body goes through hours of 'suffocation' cycles, which puts immense stress on your heart and keeps your blood pressure dangerously high the next morning. Most patients find that once they get used to it, the 'fog' lifts, the morning headaches vanish, and they finally have the energy to enjoy their day. We will work closely with you to find the most comfortable mask fit possible so it feels like a help, not a hindrance."

CASE-7

Diagnosis & Differential Diagnosis

- **Most Likely Diagnosis: Coarctation of the Aorta.**

The classic presentation of upper extremity hypertension (165/90 mmHg) compared to lower extremity hypotension (110/70 mmHg)—known as **brachial-femoral delay**—is pathognomonic. The leg fatigue (claudication) occurs because the narrowing restricts blood flow to the lower body. The continuous murmur over the back reflects turbulent flow through the narrowed segment or enlarged collateral vessels.

- **Differential Diagnoses:**
    - **Aortic Arch Hypoplasia:** A more extensive narrowing of the entire aortic arch, usually diagnosed in infancy.
    - **Takayasu Arteritis:** An inflammatory condition that can cause narrowing of large arteries; however, it typically affects females and presents with systemic symptoms like fever or weight loss.
    - **Pseudocoarctation:** A kinking of the aorta without a significant pressure gradient; however, this would not explain the 55 mmHg pressure difference or the leg fatigue.

Diagnostic Workup

- **Initial Imaging Test: Transthoracic Echocardiography (TTE)**. It is non-invasive and highly effective for visualizing the narrowing and measuring the pressure gradient across it.
- **Classic Chest X-ray Findings:**
    1. **"3" Sign:** The contour of the aorta looks like the number 3 due to pre-stenotic and post-stenotic dilation.
    2. **Rib Notching:** Caused by the erosion of the underside of the ribs by enlarged, high-pressure intercostal collateral arteries.
- **Confirmatory "Gold Standard": CT Angiography (CTA)** or **MR Angiography (MRA)** to provide detailed 3D mapping for surgical or interventional planning.

Management Plan

1. **Definitive Management: Transcatheter Stent Implantation** (preferred in adolescents/adults) or **Surgical Repair** (end-to-end anastomosis).
2. **Immediate Medical Control:** Use of Beta-blockers or ACE inhibitors to manage the upper-body hypertension before the procedure to reduce the risk of stroke or heart failure.
3. **Endocarditis Prophylaxis:** While no longer routinely required for simple coarctation, it may be discussed if there are associated heart valve issues (like a Bicuspid Aortic Valve).

Long-term Follow-up

- **Blood Pressure Monitoring:** Lifelong monitoring is mandatory. Many patients remain hypertensive or develop "rebound" hypertension even after the blockage is cleared.
- **Imaging Surveillance:** Periodic **MRI or CT every 3–5 years** to monitor for "re-coarctation" at the repair site or the development of an aortic aneurysm.
- **Cardiac Screening:** Annual screening for a **Bicuspid Aortic Valve**, which is found in up to 50–75% of patients with coarctation and can lead to valve narrowing or leakage later in life.

Patient Education

"It is a great relief to have the narrowing cleared, and it will certainly help your legs feel much better during soccer! However, it's important to think of your heart as having been 'remodeled' by the years it spent pushing against that blockage.

Even though the 'plumbing' issue is fixed, the 'electrical' or 'nervous' system that controls your blood pressure can sometimes stay set at a high level. Also, the walls of your aorta will always need a quick check-up every few years to make sure they remain strong. So, while you are significantly healthier now, we will stay 'teammates' for the long run to keep your blood pressure in the safe zone."

CASE-8

Diagnosis & Differential Diagnosis

- **Most Likely Diagnosis: Cushing's Syndrome.**

The "centripetal" obesity (weight gain in the face and abdomen), "moon facies," wide (>1 cm) purple striae, and easy bruising are classic signs of chronic cortisol excess. Given her hypertension and the absence of outside steroid use, this points to an internal overproduction of cortisol.

- **Differential Diagnoses:**
  - **Cushing's Disease:** Specifically refers to a pituitary tumor secreting ACTH; the most common endogenous cause.
  - **Adrenal Adenoma/Carcinoma:** Primary adrenal overproduction of cortisol (ACTH-independent).
  - **Ectopic ACTH Syndrome:** Often caused by small cell lung cancer or bronchial carcinoids.
  - **Polycystic Ovary Syndrome (PCOS):** Can cause weight gain and hypertension, but would not typically cause purple striae or easy bruising.

Diagnostic Workup

- **Appropriate Initial Screening Tests (Choose one of three):**

    1. **24-hour Urinary Free Cortisol (UFC):** Measures total cortisol excretion over a full day.
    2. **Overnight 1mg Dexamethasone Suppression Test (DST):** Checking if the body fails to suppress cortisol after a synthetic dose.
    3. **Late-Night Salivary Cortisol:** To assess the loss of the normal circadian rhythm of cortisol.
- **Confirmatory Step:** Once hypercortisolism is confirmed, a **Plasma ACTH level** is drawn to differentiate between a pituitary/ectopic source (High ACTH) or an adrenal source (Low ACTH).

Management Plan

1. **Surgical Intervention (Primary Priority):** Transsphenoidal surgery (for pituitary tumors) or unilateral adrenalectomy (for adrenal tumors).
2. **Medical Treatment (If no tumor is found or surgery is not an option):**
    - **Steroidogenesis Inhibitors:** Medications like **Ketoconazole**, **Metyrapone**, or **Osilodrostat** to block the adrenal glands from producing cortisol.
    - **Centrally Acting Agents: Pasireotide** (for pituitary causes).
    - **Glucocorticoid Receptor Antagonist: Mifepristone** (to block the effect of cortisol on tissues).
3. **Bilateral Adrenalectomy:** Considered a last-resort "definitive" cure if medical and pituitary-directed therapies fail.

Long-term Follow-up

- **Immediate Post-Op:** Monitoring for **Adrenal Insufficiency**. Patients often need temporary steroid replacement because their "normal" adrenal tissue has been suppressed for so long.
- **Recurrence Monitoring:** Lifelong annual screening with late-night salivary cortisol or DST, as tumors can recur years later.
- **Comorbidity Management:** Ongoing treatment for secondary conditions like osteoporosis (DEXA scans), hypertension, and diabetes until they ideally resolve post-treatment.

Patient Education

"I have some very encouraging news for you. The physical changes you are seeing—the 'moon face' and the weight in your abdomen—are directly caused by the high levels of cortisol acting on your fat cells and skin. Once we normalize your hormone levels, the 'moon face' usually resolves significantly over several months as the fat redistributes.

Regarding the purple stretch marks: while they may not disappear entirely, they typically fade from a deep purple to a much lighter, silvery-white color that is far less noticeable once the

skin stops being so thin and fragile. It takes time for the body to 'reset,' but the transformation can be quite remarkable. We will guide you through each stage of that recovery."

CASE-9

Diagnosis & Differential Diagnosis

- **Most Likely Diagnosis: Diabetic Nephropathy (Diabetic Kidney Disease).**

In a young patient with Type 1 Diabetes, the presence of persistent hypertension and "foamy" urine (a clinical sign of significant albuminuria) strongly indicates glomerular damage. This is a classic secondary cause of hypertension where the kidney's inability to handle salt and the activation of the renin-angiotensin-system drive blood pressure upward.

- **Differential Diagnoses:**
    - **Chronic Glomerulonephritis:** Other primary kidney diseases (like IgA nephropathy) can occur in diabetics, though the long history of diabetes makes diabetic nephropathy more statistically likely.
    - **Renovascular Hypertension:** Could be considered, but the high-grade proteinuria (3+) is much more characteristic of a "leaky" filter (glomerulus) than a plumbing issue in the renal artery.
    - **Minimal Change Disease:** Usually presents with sudden, massive swelling (edema), whereas diabetic damage is typically a progressive build-up.

Diagnostic Workup

- **Initial Screening & Quantification: Urine Albumin-to-Creatinine Ratio (UACR)**. This is the most important calculation as it quantifies the exact amount of protein leakage, which correlates directly with the risk of disease progression.
- **Functional Assessment: Estimated Glomerular Filtration Rate (eGFR)**. Calculated from the serum creatinine, this tells us the "percentage" of kidney function remaining.
- **Secondary Workup: Fundoscopic Eye Exam**. The presence of diabetic retinopathy (damage to eye vessels) highly correlates with the presence of nephropathy, helping to confirm the diagnosis without a biopsy.

Management Plan

1. **ACE Inhibitors or ARBs (Primary Priority):** These are the "gold standard." Unlike other BP meds, they specifically dilate the exit valve of the kidney's filtering units, reducing internal pressure and "plugging the leaks" (reducing proteinuria).
2. **SGLT2 Inhibitors:** Now recommended in international guidelines for diabetic kidney disease to slow the decline of GFR, even in Type 1 patients (with cautious monitoring for ketosis).

3. **Glycemic Control:** Intensive management of blood glucose (targeting an HbA1c < 7.0%) to prevent further microvascular damage.
4. **Strict Sodium Restriction:** Aiming for <2,300 mg/day to reduce the volume load on the kidneys.

Long-term Follow-up

- **Lab Frequency:** Monitor serum creatinine, potassium, and UACR every **3 to 6 months** depending on the stability of the GFR.
- **Potassium Monitoring:** Since ACE inhibitors and kidney disease both raise potassium, careful monitoring is required to avoid dangerous heart rhythms.
- **Target BP:** Aiming for a stricter goal of **<130/80 mmHg** to maximize kidney protection.

Patient Education

"I understand that seeing '3+ protein' or 'foamy urine' sounds very alarming. To put it simply: protein in the urine is an early warning signal, not a sign of total failure. Think of your kidneys like a fine-mesh coffee filter. Right now, the diabetes has caused some tiny tears in that mesh, allowing protein (which should stay in your blood) to 'leak' through into the urine.

The good news is that we caught this at a stage where we can take action. The specific blood pressure medicine I am prescribing acts like a 'patch' for that filter—it lowers the pressure inside the kidney so the holes don't get bigger. By controlling your blood pressure and blood sugar strictly, we can keep your kidneys working well for a very long time."

CASE-10

Diagnosis & Differential Diagnosis

- **Most Likely Diagnosis: Fibromuscular Dysplasia (FMD).**

This is a non-inflammatory, non-atherosclerotic vascular disease that most commonly affects young-to-middle-aged women. The presence of hypertension (renal artery involvement) along with pulsatile tinnitus and a carotid bruit (cervicocervical involvement) is highly suggestive of multi-vessel FMD.

- **Differential Diagnoses:**
    - **Atherosclerotic Renal Artery Stenosis:** Unlikely given her age, BMI, and status as an athlete with no cardiovascular risk factors.
    - **Takayasu Arteritis:** A large-vessel vasculitis that can cause bruits and high BP, but typically presents with systemic inflammatory markers (elevated ESR/CRP), which are absent here.
    - **Primary Hypertension:** Rare to see such severe hypertension with multi-focal bruits in a healthy 28-year-old without an underlying structural cause.

Diagnostic Workup

- **Initial Screening Tool: CT Angiography (CTA) of the Abdomen, Pelvis, and Neck**. While ultrasound is often used for kidneys, CTA is superior for FMD because it can detect the classic "string of beads" appearance across multiple vascular beds.
- **The Ultrasound "Trap":** Renal Artery Duplex Ultrasound can be a "trap" in FMD because the disease often affects the **mid-to-distal** segments of the renal artery or its branches. Standard ultrasound protocols often focus on the origin of the artery (where atherosclerosis occurs), potentially leading to a **false-negative** result in an FMD patient.

Management Plan

1. **Medical Management: ACE inhibitors or ARBs** are the first-line antihypertensives to counteract the renin-angiotensin system activation caused by renal artery narrowing.
2. **Antiplatelet Therapy:** Daily **Aspirin (75–100 mg)** is generally recommended to prevent thromboembolic events in the affected carotid or renal arteries.
3. **Revascularization (If Indicated): Percutaneous Transluminal Angioplasty (PTA)** *without* stenting is the preferred intervention if blood pressure remains uncontrolled or if there is evidence of worsening renal function. Stenting is usually avoided in FMD unless a dissection occurs during the procedure.
4. **Avoidance of Triggers:** Counseling against tobacco use and avoiding high-velocity neck manipulation.

Long-term Follow-up

- **Vascular Surveillance:** Annual **Duplex Ultrasound** of the renal and carotid arteries to monitor for progression or restenosis.
- **Comprehensive Screening:** Since FMD is often systemic, patients should have at least one **one-time head-to-pelvis cross-sectional imaging** (CTA or MRA) to screen for occult aneurysms or dissections in other vessels.
- **Clinical Monitoring:** Blood pressure and renal function (creatinine/eGFR) should be assessed every **6 months**.

Patient Education

"You are exactly right to ask about that. Because the walls of your arteries are shaped differently and may be slightly more fragile than average, we do recommend a 'safety-first' approach.

Specifically, you should **avoid chiropractic neck adjustments** or any high-velocity neck manipulations, as these could potentially cause a tear (dissection) in those carotid arteries where we heard the 'whooshing.' Regarding your running, you don't have to hang up your shoes! High-impact marathon running is generally fine, but we suggest avoiding activities

with a high risk of sudden, jarring trauma or 'whiplash' (like contact sports or bungee jumping). Our goal is to keep you active while protecting the integrity of your blood vessels."
