# Supplementary material for: Diagnostic Performance and Workup Efficiency of Large Language Models in Secondary Hypertension: A Blinded Comparative Study
Source: Diagnostics (Basel). 2026 Jul 10;16(14):2165. doi: 10.3390/diagnostics16142165 (PMC13409298; doi:10.3390/diagnostics16142165)
Supplement: Supplementary file 1 [file diagnostics-16-02165-s001.zip › Supplementary file S2/5. LLM_Earth.pdf]

CASE-1

Clinical Case Evaluation: Paroxysmal Hypertension in a Young Woman

---

1. Diagnosis & Differential Diagnosis

Most Likely Diagnosis: Pheochromocytoma

The classic triad of episodic headache, diaphoresis, and palpitations — combined with paroxysmal severe hypertension triggered by exercise, orthostasis (from volume depletion due to chronic catecholamine excess), and age of presentation — is pheochromocytoma until proven otherwise.

Differential Diagnoses:

- **Paraganglioma** — extra-adrenal catecholamine-secreting tumor; clinically identical but located outside the adrenal gland
- **Essential hypertension with anxiety/panic disorder** — can mimic episodic symptoms but rarely causes BP of 210/115 in a 34-year-old; no orthostasis expected
- **Hyperthyroidism/thyroid storm** — causes tachycardia and sweating but hypertension is typically mild and not paroxysmal
- **Carcinoid syndrome** — episodic flushing and tachycardia, but hypertension is less prominent and diarrhea is usually present
- **Cocaine/stimulant use** — must be excluded by history and urine toxicology

---

2. Diagnostic Workup

Initial Screening Test (Most Sensitive): 24-hour urinary fractionated metanephrines and catecholamines, or preferably **plasma free metanephrines** (sensitivity ~97–99% per Endocrine Society Guidelines 2014). Plasma free metanephrines are the single most sensitive test and are now preferred as first-line in high-risk patients like this one.

Confirmatory & Localization:

- **CT abdomen/pelvis with contrast** — first-line imaging once biochemical confirmation is established (do NOT image before biochemical proof)
- **MRI** preferred in younger patients, pregnant women, or suspected paraganglioma
- **^{123}I-MIBG scintigraphy or ^{68}Ga-DOTATATE PET/CT** — for metastatic, multifocal, or extra-adrenal disease
- **Genetic testing** (RET, VHL, SDHB/C/D, NF1, MAX) — recommended in all patients, especially given young age; up to 40% of pheochromocytomas are hereditary

---

3. Management Plan

Step 1 — Alpha-blockade FIRST (mandatory, non-negotiable): Phenoxybenzamine (non-competitive alpha-blocker) 10 mg twice daily, titrated over 10–14 days preoperatively.

Alternatively, **doxazosin** (competitive alpha-blocker) is used in many centers with a favorable side-effect profile.

Step 2 — Beta-blockade ONLY after adequate alpha-blockade: Propranolol or atenolol to control reflex tachycardia. Beta-blockers must NEVER be started before alpha-blockade — doing so leaves alpha receptors unopposed, potentially precipitating a fatal hypertensive crisis.

Step 3 — Liberal salt and fluid intake during preoperative preparation to expand contracted plasma volume and reduce post-resection hypotension risk.

Step 4 — Surgical resection: Laparoscopic adrenalectomy is the gold standard for localized adrenal pheochromocytoma. Open surgery is reserved for large (>6 cm) or suspected malignant tumors.

Intraoperative management: Experienced anesthesia team with IV **phentolamine or nicardipine** on standby for hypertensive surges; **magnesium sulfate** as adjunct; meticulous surgical handling to minimize catecholamine release.

---

4. Long-term Follow-up

Per Endocrine Society Guidelines and recent consensus statements:

Biochemical monitoring:

- **Plasma free metanephrines or 24-hour urinary fractionated metanephrines** at **6 weeks postoperatively** to confirm biochemical cure
- Then **annually for at least 10 years** — lifelong follow-up is now strongly recommended given recurrence and malignancy risk
- For patients with **SDHx mutations**: annual biochemical testing **lifelong**

Imaging:

- Repeat cross-sectional imaging (CT or MRI) **only if biochemical recurrence is detected** or in high-risk genetic cases
- **^{123}I-MIBG or ^{68}Ga-DOTATATE PET/CT** for metastatic surveillance in SDHB mutation carriers (highest malignancy risk ~25–35%)

Genetic counseling:

- All first-degree relatives should be offered genetic testing if a germline mutation is identified

Blood pressure monitoring:

- At every follow-up visit; monitor for residual or recurrent hypertension

---

5. Patient Education — *"Will I be off blood pressure meds forever after surgery?"*

"That's a really important question, and I completely understand why you're hoping for a clean break after going through all of this.

The encouraging news is that for most patients — especially someone young like you with what appears to be a single tumor — surgery is curative, and the majority of patients do see their blood pressure normalize within weeks to months after the operation, allowing them to come off medications entirely.

However, I want to be fully transparent with you: about 20–30% of patients may still have some degree of high blood pressure after surgery. This can happen for a few reasons — sometimes the blood vessels have undergone changes from years of high pressure, or there may be an underlying tendency toward hypertension that was simply unmasked. In rare cases, there can be residual or recurrent tumor tissue.

This is also why we will test your blood and urine about six weeks after surgery and then annually — to make sure everything stays in remission.

So the honest answer is: there's a very good chance you'll be medication-free, and that is absolutely our goal. But we'll follow you closely and make that determination together based on how your body responds after surgery. You won't be navigating this alone."

Key Teaching Point: Alpha-blockade before beta-blockade is the cardinal preoperative rule. Reversing this order is a potentially fatal error and a classic board and clinical exam question.

CASE-2

Clinical Case Evaluation: Resistant Hypertension with Hypokalemia

---

1. Diagnosis & Differential Diagnosis

Most Likely Diagnosis: Primary Hyperaldosteronism (Conn's Syndrome)

The constellation of resistant hypertension on three agents (including a diuretic), spontaneous hypokalemia, elevated PAC with suppressed PRA, muscle weakness, and fatigue creates a textbook presentation. The **Aldosterone-to-Renin Ratio (ARR)** in this patient would be markedly elevated, confirming autonomous aldosterone secretion independent of the renin-angiotensin system.

Differential Diagnoses:

- **Unilateral aldosterone-producing adenoma (APA)** — ~35% of primary hyperaldosteronism cases; typically more severe hypokalemia and higher aldosterone levels; surgically curable
- **Bilateral adrenal hyperplasia (BAH/IHA)** — ~60% of cases; milder biochemical derangement; managed medically
- **Glucocorticoid-remediable aldosteronism (GRA/FH-I)** — rare autosomal dominant; aldosterone suppressed by dexamethasone; younger patients with strong family history
- **Secondary hyperaldosteronism** — elevated renin AND aldosterone (renal artery stenosis, heart failure, cirrhosis); excluded here by suppressed PRA

- **Apparent mineralocorticoid excess (AME)** — cortisol-mediated; low aldosterone AND low renin
- **Liddle syndrome** — gain-of-function ENaC mutation; low aldosterone AND low renin; not mineralocorticoid-mediated

---

2. Diagnostic Workup

Step 1 — Confirmatory Testing: Oral sodium loading test or **fludrocortisone suppression test (FST)** — gold standard for confirmation. Failure to suppress aldosterone below 6 ng/dL (oral load) or 6–10 ng/dL (FST) confirms autonomous secretion. Salt infusion test (IV saline, 2L over 4 hours) is widely used due to practicality.

Step 2 — Subtype Differentiation: CT abdomen (adrenal protocol) — first-line imaging to identify gross adenoma vs. hyperplasia and exclude adrenocortical carcinoma. However, CT alone is insufficient for surgical decision-making due to ~50% false localization rate.

Gold Standard for Subtype Differentiation: Adrenal Vein Sampling (AVS) — simultaneous bilateral sampling of adrenal vein aldosterone and cortisol. A **lateralization index ≥4:1** (aldosterone/cortisol ratio) confirms unilateral disease. This is mandatory before surgery in all patients >35 years, per Endocrine Society 2016 Guidelines, as CT frequently misclassifies subtype.

---

3. Management Plan

A. Unilateral Adenoma: Laparoscopic unilateral adrenalectomy — treatment of choice. Results in biochemical cure in ~100% and clinical BP cure in ~50%, with improvement in the remainder.

Preoperative preparation:

- Correct hypokalemia with oral potassium supplementation
- **Spironolactone or eplerenone** to normalize BP and potassium preoperatively
- Adequate duration: 4–6 weeks minimum before surgery

B. Bilateral Adrenal Hyperplasia — First-Line Medical Therapy:

Mineralocorticoid Receptor Antagonists (MRAs):

- **Spironolactone** — first-line agent; start at **12.5–25 mg/day**, titrate to **25–100 mg/day**; most cost-effective with robust evidence
- **Eplerenone** — preferred when spironolactone is poorly tolerated (due to anti-androgenic side effects); start at **25 mg twice daily**; more selective but less potent per mg
- Target: serum potassium normalization and BP <130/80 mmHg (ESH/ESC 2023 targets)
- Add existing antihypertensives as needed; **avoid ACE inhibitors/ARBs** if renin remains suppressed

Lifestyle interventions (adjunct, all patients):

- Low-sodium diet (<2g/day) — reduces aldosterone-driven fluid retention significantly
- Weight reduction, aerobic exercise, alcohol moderation

---

4. Long-term Follow-up Protocol

Post-adrenalectomy (Unilateral APA):

- **At 1–3 months:** Repeat serum electrolytes, PAC, PRA/direct renin, and 24-hour urinary aldosterone to confirm biochemical cure
- **At 6 months and 12 months:** BP assessment, repeat biochemistry; gradually wean antihypertensives guided by response
- **Annually thereafter for 5 years:** Biochemical panel (PAC, PRA, potassium); monitor for contralateral hyperplasia development
- **Genetic testing:** Offer in patients <20 years or with family history (KCNJ5, CYP11B2 mutations; ARMC5 for bilateral disease)

Medical Management (Bilateral Hyperplasia):

- **Every 3–6 months initially:** Serum potassium, renal function, BP review; titrate spironolactone/eplerenone dose
- **Annually:** PAC, PRA, metabolic panel, eGFR, urine albumin-to-creatinine ratio (aldosterone drives renal and cardiac fibrosis independently of BP)
- **Cardiovascular risk assessment annually:** ECG for LVH, echocardiogram every 3–5 years (aldosterone causes disproportionate cardiac remodeling vs. essential hypertension)
- **Repeat adrenal CT only if:** Biochemical escape from control, suspicion of developing adenoma on background of hyperplasia, or adrenocortical carcinoma concern

---

5. Patient Education — *Male Patient Presenting with Gynecomastia*

Clinical context: Gynecomastia in this setting is a well-recognized anti-androgenic side effect of **spironolactone**, occurring in up to **10–25% of male patients**, due to its off-target binding to androgen receptors and progesterone receptors.

"I really appreciate you bringing this up, and I want you to know this is something we take seriously — it can understandably be distressing and affect your quality of life.

The gynecomastia you're experiencing is most likely a side effect of the spironolactone we started for your condition. Spironolactone is a very effective medication, but in men, it sometimes interferes with male hormone pathways, which can cause breast tissue to enlarge. This is not dangerous, but I completely understand it's uncomfortable and concerning.

Here is what we can do for you:

*The most straightforward solution is to switch you to a medication called **eplerenone**. It works in exactly the same way as spironolactone — blocking the hormone aldosterone that is*

causing your high blood pressure and low potassium — but it is much more selective and does not have the anti-hormonal side effects. Most men who switch see the gynecomastia gradually improve over several weeks to months.

If eplerenone is not available or covered by your insurance, we can also consider ***dose reduction*** *of spironolactone, though this must be balanced against keeping your blood pressure and potassium well controlled.*

We will recheck your labs and blood pressure after the switch to make sure everything remains stable. Please don't stop any medication on your own before we speak — but rest assured, we have a good solution for you."

Key Teaching Point: AVS is the non-negotiable gold standard before adrenalectomy — CT alone will lead to wrong-side surgery or unnecessary surgery in a significant proportion of cases. Never operate based on imaging alone in primary hyperaldosteronism.

CASE-3

Clinical Case Evaluation: ACE Inhibitor-Induced Acute Kidney Injury in a Hypertensive Smoker

---

1. Diagnosis & Differential Diagnosis

Most Likely Diagnosis: Renovascular Hypertension due to Atherosclerotic Renal Artery Stenosis (RAS)

The clinical fingerprints here are unmistakable: a heavy smoker with established peripheral vascular disease (a systemic atherosclerotic disease marker), new-onset hypertension, an **abdominal bruit** in the left upper quadrant (renal artery territory), and — most critically — a **>30% rise in serum creatinine within 2 weeks of ACE inhibitor initiation**. This creatinine rise is the pathognomonic functional signature of hemodynamically significant bilateral RAS or RAS in a solitary functioning kidney, where glomerular filtration is entirely dependent on angiotensin II-mediated efferent arteriolar tone.

Differential Diagnoses:

- **Fibromuscular Dysplasia (FMD)** — non-inflammatory, non-atherosclerotic arterial disease; classically affects young-to-middle-aged women; "string of beads" appearance on imaging; less likely here given age, sex, and smoking history but must be considered
- **Atheroembolic renal disease** — cholesterol crystal embolization causing AKI; associated with recent vascular procedures or anticoagulation; livedo reticularis and eosinophilia may be present
- **Contrast nephropathy** — not applicable here; no contrast exposure reported
- **ACE inhibitor-induced functional AKI without RAS** — can occur in severe heart failure or volume depletion; clinical context does not support this
- **Renal artery dissection or thrombosis** — acute presentation, usually with flank pain; less consistent with this timeline

- **Aortic coarctation** — would present earlier in life with upper/lower limb BP discrepancy

---

2. Diagnostic Workup

Immediate Action: Discontinue the ACE inhibitor now. Do not rechallenge until anatomy is fully defined. Monitor creatinine, potassium, and urine output closely over 48–72 hours. Transition to a calcium channel blocker (e.g., amlodipine) for interim BP control — safe in RAS without efferent arteriolar dependence concern.

Initial Non-Invasive Screening Tool of Choice:

Renal Duplex Ultrasonography (Doppler Ultrasound)

- First-line per ACC/AHA and ESH/ESC guidelines
- Evaluates renal artery peak systolic velocity (PSV >180–200 cm/s suggests significant stenosis), resistive index, renal size asymmetry (>1.5 cm difference is significant), and post-stenotic turbulence
- Advantages: no radiation, no nephrotoxic contrast, widely available, cost-effective
- Limitation: operator-dependent; technically difficult in obese patients or those with bowel gas

Second-Line Non-Invasive Options:

- **CT Angiography (CTA)** — excellent sensitivity/specificity (~95%); limited by contrast nephrotoxicity risk in this patient with already-compromised renal function
- **MR Angiography (MRA) with gadolinium** — preferred over CTA when eGFR allows; however, gadolinium is contraindicated if eGFR <30 due to nephrogenic systemic fibrosis risk
- **Non-contrast MRA (time-of-flight or phase contrast)** — emerging as safe alternative in CKD patients

Gold Standard Confirmatory Test: Digital Subtraction Angiography (DSA) / Conventional Catheter Angiography — provides definitive anatomical characterization and allows simultaneous intervention (angioplasty/stenting); reserved for cases where non-invasive imaging is inconclusive or revascularization is planned.

---

3. Management Plan

Tier 1 — Medical Therapy (All Patients, Including Pre- and Post-Revascularization):

- **Aggressive statin therapy** (high-intensity: atorvastatin 40–80 mg or rosuvastatin 20–40 mg) — stabilizes plaque, reduces cardiovascular events, may slow stenosis progression
- **Antiplatelet therapy** — aspirin 75–100 mg daily; clopidogrel if aspirin-intolerant or post-stenting

- **BP control:** Calcium channel blockers (amlodipine) and loop diuretics are safest in bilateral RAS; **ACE inhibitors and ARBs are contraindicated in bilateral RAS or RAS in solitary kidney**
- **Smoking cessation** — absolutely mandatory; continued smoking accelerates atherosclerotic progression and negates revascularization benefit
- **Glycemic and lipid targets** per current cardiovascular guidelines

Tier 2 — Revascularization (Refractory/High-Risk Cases):

Percutaneous Transluminal Renal Angioplasty (PTRA) with Stenting — definitive management for refractory cases:

Indications (per ACC/AHA 2013 and ESC 2017 guidelines):

- Hemodynamically significant stenosis (>70% by angiography or >60% with pressure gradient >20 mmHg) **plus** at least one of:
  - Resistant hypertension despite ≥3 agents including a diuretic
  - Recurrent flash pulmonary edema (Pickering syndrome) — compelling indication
  - Progressive ischemic nephropathy / deteriorating renal function
  - Bilateral RAS or RAS in solitary kidney
  - Inability to tolerate antihypertensive medications

Important caveat: The ASTRAL and CORAL trials showed no significant benefit of stenting over optimal medical therapy in unselected patients — patient selection is therefore critical. Revascularization is most beneficial in hemodynamically significant stenosis with preserved downstream renal parenchyma and recent-onset hypertension.

Surgical Revascularization (Aortorenal bypass or endarterectomy):

- Reserved for failed angioplasty, complex anatomy, or concomitant aortic surgery need
- Higher risk in this patient population given PVD and smoking history

---

4. Long-term Follow-up Protocol

Immediate Post-ACE Inhibitor Discontinuation:

- Recheck **serum creatinine, BUN, potassium within 48–72 hours** to confirm stabilization or recovery

Post-Revascularization Monitoring:

- **At 1 month:** Serum creatinine, eGFR, electrolytes, BP assessment, urine protein-to-creatinine ratio
- **At 3 and 6 months:** Repeat renal Doppler ultrasound to assess stent patency and restenosis (occurs in ~15–20% within 12 months); repeat biochemistry
- **Annually thereafter:** Renal Doppler ultrasound, serum creatinine, eGFR, urine albumin, BP review, cardiovascular risk factor assessment

- **CTA or DSA** only if Doppler suggests restenosis or clinical deterioration (worsening BP control, rising creatinine)

Ongoing Cardiovascular Surveillance:

- **Ankle-Brachial Index (ABI)** annually — monitor PVD progression
- **Lipid panel** every 6–12 months on statin therapy
- **Smoking cessation counseling** at every visit — pharmacotherapy (varenicline, NRT) if not already initiated
- **Renal function trajectory:** If eGFR declines >25% from baseline over any 6-month period, repeat full imaging workup

---

5. Patient Education — *"Why did the blood pressure medicine make my kidney function worse?"*

"That is such an important question, and I'm really glad you asked it — because understanding what happened will help us keep you safe going forward.

Your kidneys have been working under very difficult conditions. The artery that supplies blood to one or possibly both of your kidneys has become significantly narrowed — most likely from the same process of hardening and furring of the arteries that has affected the blood vessels in your legs. Because of this narrowing, your kidney has been receiving less blood flow than it needs.

Now, here is the key part: your body is remarkably clever. When blood flow drops, your kidney releases a hormone called angiotensin II, which tightens a specific valve at the exit of the kidney's tiny filters. This tightening actually maintains the pressure inside the filters and keeps them working — it's your kidney's way of compensating and protecting itself.

The blood pressure medication we started — called an ACE inhibitor — works by blocking angiotensin II throughout the body, which is normally exactly what we want for high blood pressure. But in your situation, by blocking that hormone, we inadvertently relaxed that protective valve in your kidney. With the narrowed artery upstream AND the valve now relaxed, the pressure inside your kidney filters dropped too low, and they temporarily struggled to do their job — which is why your creatinine went up.

This is actually a very valuable clue. It told us there is likely a significant narrowing in your renal artery that we now need to investigate and treat properly.

We have already stopped that medication, and we expect your kidney function to recover. We are now switching you to a different, safer blood pressure medication in the meantime, and we are arranging the appropriate tests to look at those kidney arteries directly.

The good news is — this is treatable. And catching it now means we can protect both your blood pressure and your kidneys going forward."

Key Teaching Point: ACE inhibitor or ARB-induced AKI (>30% creatinine rise) in a patient with vascular risk factors and an abdominal bruit is RAS until proven otherwise. This is not a contraindication to diagnosis — it is the diagnosis. Doppler ultrasound first, angiography when intervention is planned.

CASE-4

Clinical Case Evaluation: Hypercalcemia with Hypertension and Nephrolithiasis

---

1. Diagnosis & Differential Diagnosis

Most Likely Diagnosis: Primary Hyperparathyroidism (PHPT)

This case is a masterclass in recognizing the classic mnemonic **"Bones, Stones, Groans, and Psychic Moans"** — the four cardinal manifestations of primary hyperparathyroidism. This 50-year-old woman presents with hypercalcemia, recurrent calcium oxalate nephrolithiasis, constipation (groans), and neurocognitive symptoms including brain fog and mood disturbance (psychic moans), all occurring in the demographic sweet spot — postmenopausal women are the highest-risk group, with PHPT being the most common cause of hypercalcemia in the outpatient setting.

The pathophysiology is autonomous, PTH-driven calcium mobilization from bone, enhanced renal calcium reabsorption, and increased 1,25-dihydroxyvitamin D synthesis causing enhanced gut calcium absorption — all operating independently of normal feedback inhibition.

Differential Diagnoses:

- **Malignancy-associated hypercalcemia** — most common cause of hypercalcemia in hospitalized patients; mediated by PTHrP (humoral hypercalcemia of malignancy) or direct osteolytic metastases; PTH will be suppressed (not elevated); typically more severe and acute onset; must be actively excluded
- **Familial Hypocalciuric Hypercalcemia (FHH)** — autosomal dominant inactivating mutation of CaSR gene; benign lifelong mild hypercalcemia; differentiated by 24-hour urine calcium-to-creatinine clearance ratio <0.01; PTH is normal or mildly elevated; surgery is NOT indicated and must not be performed
- **Vitamin D toxicity** — elevated 25-OH vitamin D; PTH suppressed; history of supplementation
- **Sarcoidosis and other granulomatous diseases** — elevated 1,25-dihydroxyvitamin D due to unregulated macrophage hydroxylation; PTH suppressed; ACE level may be elevated
- **Thiazide diuretic use** — reduces urinary calcium excretion, unmasking latent hypercalcemia; medication review essential
- **Multiple Endocrine Neoplasia Type 1 (MEN-1)** — PHPT is the most common and earliest manifestation; always consider in younger patients or those with pituitary/pancreatic pathology; screen with prolactin, gastrin, fasting glucose
- **MEN-2A** — PHPT associated with medullary thyroid carcinoma and pheochromocytoma; less common but important to exclude
- **Lithium therapy** — shifts the calcium set-point; medication history critical

---

2. Diagnostic Workup

Confirmatory Lab Combination:

The diagnosis of PHPT is confirmed by the simultaneous finding of:

Elevated or inappropriately normal serum PTH + Hypercalcemia (elevated total and/or ionized calcium)

In any other cause of hypercalcemia, PTH should be fully suppressed by negative feedback. A PTH that is elevated or even in the upper-normal range in the setting of hypercalcemia is physiologically inappropriate and diagnostic of PTH-dependent hypercalcemia — i.e., PHPT.

Complete Diagnostic Panel:

- **Serum intact PTH (iPTH)** — two-site immunoradiometric assay; gold standard PTH measurement
- **Serum total and ionized calcium** — ionized calcium is the physiologically active fraction and more reliable in hypoalbuminemia; correct total calcium for albumin: add 0.8 mg/dL per 1 g/dL albumin below 4.0
- **Serum phosphorus** — typically low/suppressed (PTH promotes phosphaturia)
- **Serum creatinine and eGFR** — assess renal function; nephrolithiasis can cause CKD
- **24-hour urine calcium and creatinine** — essential to exclude FHH (ratio <0.01 suggests FHH, not PHPT); also quantifies stone risk
- **25-hydroxyvitamin D** — vitamin D deficiency is common in PHPT and should be corrected cautiously post-surgery; also excludes vitamin D toxicity
- **1,25-dihydroxyvitamin D** — elevated in PHPT due to PTH-stimulated renal hydroxylation
- **Alkaline phosphatase (bone-specific)** — marker of bone turnover; elevated in osteitis fibrosa cystica
- **Serum PTHrP** — if malignancy suspected and PTH is suppressed

Localization Imaging (Pre-surgical, NOT diagnostic):

- **Technetium-99m sestamibi scintigraphy (MIBI scan)** — first-line for parathyroid adenoma localization; sensitivity ~80–90% for single adenoma
- **Neck ultrasound** — complementary to MIBI; operator-dependent; identifies concomitant thyroid pathology
- **4D-CT parathyroid protocol** — superior sensitivity for ectopic or multiglandular disease; used when MIBI and ultrasound are discordant
- **MRI neck/chest** — for ectopic mediastinal glands
- **Intraoperative PTH monitoring** — rapid PTH assay during surgery; >50% drop from baseline within 10 minutes of gland excision confirms cure (Miami criterion)

Bone Assessment:

- **DEXA scan** — mandatory; assess at lumbar spine, hip, and **distal 1/3 radius** (cortical bone most affected by PTH-mediated resorption); baseline and post-surgical monitoring

3. Management Plan

The Only Curative Treatment: Parathyroidectomy

Surgical Indications (Endocrine Society / AACE / AAES 2022 Guidelines) — operate if ANY of the following:

- Serum calcium >1.0 mg/dL above upper limit of normal (>11.5 mg/dL) — this patient approaches threshold
- eGFR <60 mL/min/1.73m²
- **Nephrolithiasis or nephrocalcinosis** — present in this patient; compelling indication regardless of calcium level
- DEXA T-score ≤ −2.5 at any site, or vertebral fracture on imaging
- Age <50 years
- 24-hour urine calcium >400 mg/day with elevated stone risk profile
- Patient preference even without meeting above criteria, if surgery is safe

This patient meets surgical criteria based on recurrent nephrolithiasis alone.

Surgical Approach:

- **Minimally invasive parathyroidectomy (MIP)** — preferred when single adenoma is localized concordantly on MIBI and ultrasound; lower morbidity, shorter operative time, equivalent cure rates (~95–98%)
- **Bilateral neck exploration** — gold standard when imaging is discordant, multiglandular disease suspected, MEN-1 (all four glands at risk), or familial PHPT
- **Intraoperative PTH monitoring** — mandatory during MIP to confirm complete resection

Post-Surgical Management — "Hungry Bone Syndrome":

- Anticipate hypocalcemia, hypomagnesemia, and hypophosphatemia in patients with significant bone disease
- Prophylactic oral **calcium carbonate 1–2g TID** and **calcitriol 0.25–0.5 mcg BID** starting perioperatively
- Monitor serum calcium every 6–12 hours for 24–48 hours post-surgery

Medical Management (Non-Surgical Candidates Only):

- **Cinacalcet (calcimimetic)** — reduces serum calcium by increasing CaSR sensitivity; 30–90 mg twice daily; does NOT improve bone density or reduce stone risk; purely biochemical control
- **Bisphosphonates (alendronate)** — improve bone density but do not lower calcium effectively
- **Adequate hydration** — reduces stone risk and hypercalcemic symptoms

- **Avoid:** thiazide diuretics (worsen hypercalcemia), lithium, prolonged immobilization, dehydration

---

4. Long-term Follow-up Protocol

Post-Parathyroidectomy (Confirmed Cure):

- **24–48 hours post-op:** Serum calcium, phosphorus, magnesium, PTH — detect hungry bone syndrome early
- **At 2 weeks:** Repeat calcium, PTH, renal function — confirm normocalcemia and PTH suppression
- **At 3–6 months:** Full biochemical panel (calcium, PTH, phosphorus, 25-OH vitamin D, creatinine, 24-hour urine calcium); DEXA scan at 1 year to document bone density recovery
- **Annually for 5 years:** Serum calcium and PTH — recurrence rate ~5%; multiglandular disease or MEN may present later
- **Renal imaging (ultrasound or low-dose CT):** At baseline and every 2–3 years to monitor for new stone formation; stone risk decreases but does not disappear post-surgery
- **DEXA scan:** At 1–2 years post-surgery and every 2–3 years thereafter; expect significant bone density recovery, particularly at lumbar spine and hip over 2–5 years
- **Vitamin D optimization:** Maintain 25-OH vitamin D at 40–60 ng/mL; supplement if deficient (safe post-cure)

Surveillance for MEN Syndromes:

- If age <40, multiglandular disease, or family history: genetic testing for **MEN1 gene mutation**, **HRPT2/CDC73** (hyperparathyroidism-jaw tumor syndrome), **CaSR mutation** (FHH)
- MEN-1 patients: annual prolactin, fasting gastrin, fasting insulin/glucose, pituitary MRI every 3–5 years

Persistent or Recurrent Hyperparathyroidism:

- PTH remains elevated post-op: re-image with 4D-CT and sestamibi; consider ectopic gland or supernumerary tissue
- Refer to high-volume parathyroid surgery center for re-exploration

---

5. Patient Education — *"Will my brain fog and mood go away after surgery?"*

"This is one of the questions I hear most often from patients before surgery — and it's one of my favorite questions to answer, because the answer is genuinely encouraging for most people.

What you're experiencing — the fogginess, the difficulty concentrating, the low mood, perhaps the fatigue and irritability — these are all real, recognized effects of having too much calcium in your blood for an extended period. Calcium plays a critical role in how your brain

cells communicate with one another, and when levels are chronically elevated, it disrupts that communication in subtle but meaningful ways. You are not imagining it, and it is not 'just stress.'

The research tells us that the majority of patients — particularly those your age with the types of symptoms you're describing — report meaningful improvement in cognitive clarity, mood, and energy within weeks to months after successful surgery. Many patients describe it as a 'lifting of a fog' they hadn't even fully realized had been there. Some studies show improvements in quality of life, anxiety scores, and depressive symptoms that continue for up to 12 months post-operatively.

However, I want to be completely honest with you, because I believe you deserve a realistic picture: the response is not identical in every patient. Those with milder or more recent symptoms tend to recover most fully and quickly. If the hypercalcemia has been present for many years, some degree of neurological adaptation may have occurred, and recovery — while still likely — may be more gradual.

What I can tell you with confidence is this: surgery gives you the best possible chance of feeling like yourself again. We will monitor you closely after the procedure, and if mood symptoms persist beyond a few months, we will evaluate whether additional support — such as a referral to a mental health colleague — might be beneficial.

You have been living with this condition affecting your brain chemistry. It is entirely reasonable — and medically supported — to expect to feel significantly better. Let's get you to surgery and get your calcium back where it belongs."

Key Teaching Point: PHPT is the great mimicker of psychiatric and cognitive disease in middle-aged women. Nephrolithiasis alone is a sufficient surgical indication regardless of calcium level. Never perform surgery based on imaging alone — localization guides the surgeon, but the biochemistry makes the diagnosis. Always exclude FHH before referring for parathyroidectomy, as surgery is not only ineffective but harmful in that condition.

CASE-5

Clinical Case Evaluation: Hypertension in a Young Asymptomatic Athlete

---

1. Diagnosis & Differential Diagnosis

Most Likely Diagnosis: Primary (Essential) Hypertension

This is a diagnosis of exclusion, arrived at after a methodical and thorough secondary hypertension workup has returned negative. The patient is a 19-year-old male with a BMI of 24 (normal), no smoking history, no family history of premature cardiovascular disease, normal femoral pulses (excludes aortic coarctation), normal fundoscopy (no hypertensive retinopathy suggesting chronicity), normal renal ultrasound (excludes renovascular and parenchymal disease), normal thyroid studies (excludes hyperthyroidism), and normal metanephrines (excludes pheochromocytoma). With all secondary causes excluded, primary hypertension is the appropriate diagnosis.

However, before committing this young man to a lifelong diagnosis and potential pharmacotherapy, two important considerations must be addressed: white coat hypertension and exercise-induced or athletic hemodynamic adaptation.

Differential Diagnoses:

- **White Coat Hypertension (WCH)** — BP elevation exclusively in clinical settings due to sympathetic arousal; prevalence up to 30–40% in young adults; carries lower cardiovascular risk than sustained hypertension but is not entirely benign; must be actively excluded before diagnosis and treatment
- **Masked Hypertension** — normal office BP but elevated ambulatory/home BP; paradoxically higher cardiovascular risk than white coat hypertension; more common in young males and athletes; would be missed without out-of-office monitoring
- **Aortic Coarctation** — congenital narrowing of the descending aorta; presents with upper extremity hypertension, diminished/delayed femoral pulses, rib notching on chest X-ray, and systolic murmur; excluded here by normal femoral pulses and normal exam
- **Renal Parenchymal Disease** — most common secondary cause in young patients overall; glomerulonephritis, polycystic kidney disease, reflux nephropathy; excluded by normal renal ultrasound and normal urinalysis
- **Renovascular Hypertension (FMD in young patients)** — fibromuscular dysplasia is the predominant cause in young women; less common in young males; excluded by normal renal ultrasound and absence of abdominal bruit
- **Primary Hyperaldosteronism** — consider if hypokalemia present; normal labs make this unlikely but ARR can be checked if clinical suspicion arises
- **Hyperthyroidism** — tachycardia, systolic hypertension, weight loss; excluded by normal thyroid studies
- **Stimulant/Performance-Enhancing Drug Use** — anabolic steroids, amphetamines, EPO, NSAIDs (chronic use raises BP); must be explored tactfully but directly in a young male athlete; this is a clinically critical and frequently overlooked secondary cause in this demographic
- **Obstructive Sleep Apnea** — increasingly recognized in young athletes, particularly those with high muscle mass and larger neck circumference; causes nocturnal and morning hypertension; worth screening with STOP-BANG questionnaire

---

2. Diagnostic Workup

Ruling Out White Coat Hypertension — Before Any Medication:

24-Hour Ambulatory Blood Pressure Monitoring (ABPM) This is the gold standard recommended by ESH/ESC 2023 and ACC/AHA 2017 guidelines before initiating antihypertensive pharmacotherapy in any young patient, and is particularly indispensable here. ABPM provides:

- Daytime average BP (awake period)
- Nighttime average BP (sleep period) — nocturnal dipping pattern is physiologically important
- 24-hour mean BP
- BP variability and load calculations

- Elimination of white coat effect

Diagnostic Thresholds (ESH/ESC 2023):

- Normal ABPM: 24-hour <130/80, daytime <135/85, nighttime <120/70 mmHg
- White coat hypertension: Elevated office BP + normal ABPM values
- Masked hypertension: Normal office BP + elevated ABPM values
- Sustained hypertension: Elevated both office and ABPM values

Home Blood Pressure Monitoring (HBPM) is an acceptable and cost-effective alternative where ABPM is unavailable — morning and evening readings over 7 days (discard day 1), average of remaining readings; daytime threshold >135/85 mmHg is abnormal.

Additional Workup Before Treatment Decision:

- **Urinalysis with microscopy and urine albumin-to-creatinine ratio** — detect early renal involvement or subclinical glomerular disease
- **Fasting glucose and HbA1c** — insulin resistance and early metabolic syndrome can drive hypertension even in lean individuals
- **Fasting lipid panel** — global cardiovascular risk stratification
- **Serum electrolytes, creatinine, eGFR** — baseline renal function
- **ECG** — screen for left ventricular hypertrophy (LVH), conduction abnormalities, or athletic heart changes
- **Echocardiogram** — consider if ECG shows LVH or if athlete's heart vs. hypertensive heart distinction is clinically relevant; also provides baseline cardiac geometry
- **Urine toxicology / targeted history** regarding anabolic steroid, NSAID, stimulant, or recreational drug use — essential in young male athlete

---

3. Management Plan

First-Line Intervention: Structured Lifestyle Modification (3–6 Month Trial Before Pharmacotherapy)

Per ACC/AHA 2017, ESH/ESC 2023, and Endocrine Society guidelines, a young patient with Stage 1 hypertension (130–139/80–89 mmHg by ACC/AHA; Grade 1 by ESH: 140–159/90–99 mmHg) and **no evidence of end-organ damage, diabetes, CKD, or established cardiovascular disease** should receive an intensive, structured, time-defined lifestyle intervention trial before any medication is initiated.

Evidence-Based Lifestyle Interventions (in order of BP-lowering magnitude):

Dietary Sodium Restriction — target <2g sodium/day (<5g salt/day); reduces systolic BP by 5–10 mmHg in sodium-sensitive individuals; most impactful single dietary intervention; athletes often consume high-sodium processed foods and sports drinks without awareness

DASH Diet (Dietary Approaches to Stop Hypertension) — rich in fruits, vegetables, whole grains, low-fat dairy, lean protein, potassium, magnesium, and calcium; reduces systolic BP by 8–14 mmHg; synergistic with sodium restriction

Potassium optimization — dietary potassium 3,500–5,000 mg/day through whole foods (bananas, sweet potatoes, leafy greens, legumes); counteracts sodium's pressor effect; reduces systolic BP 4–5 mmHg

Aerobic exercise optimization — paradoxically, while he is an athlete, the type of exercise matters enormously. Dynamic aerobic exercise (running, cycling, swimming) lowers resting BP by 5–8 mmHg. Heavy isometric/resistance training (powerlifting, heavy weightlifting) acutely raises BP dramatically and may sustain elevation; exercise prescription should emphasize aerobic components

Alcohol restriction — if applicable; even moderate intake raises BP; each drink/day reduction lowers systolic BP ~1 mmHg

Weight maintenance and stress reduction — BMI is already normal; mindfulness-based stress reduction and adequate sleep (7–9 hours) are underappreciated BP modulators in competitive athletes

Caffeine and stimulant review — pre-workout supplements, energy drinks, and caffeine in high doses acutely and chronically raise BP; review and restrict

Re-evaluate at 3 months: If BP normalizes on ABPM/HBPM → continue lifestyle, monitor every 6 months. If BP remains ≥140/90 mmHg with confirmed ambulatory elevation and no response to lifestyle → initiate pharmacotherapy.

Pharmacotherapy (if lifestyle fails or end-organ damage is present):

- **ACE inhibitor (ramipril/lisinopril) or ARB (losartan)** — first-line in young patients; favorable metabolic profile; renoprotective; well tolerated in athletes
- **Calcium channel blockers (amlodipine)** — excellent BP lowering; no metabolic side effects; suitable for athletes
- **Thiazide-like diuretics** — effective but risk of volume depletion, cramping, and electrolyte disturbance in competitive athletes; use with caution
- **Beta-blockers** — generally avoided as first-line in young athletes; reduce exercise capacity, blunt physiological heart rate response, and are **banned in certain precision sports** (archery, shooting) by WADA — this is a critical sports medicine consideration

---

4. Long-term Follow-up Protocol

During Lifestyle Modification Trial:

- **Every 4–6 weeks:** Office BP measurement and HBPM diary review; assess adherence, dietary sodium recall, supplement and stimulant use
- **At 3 months:** Repeat ABPM to objectively assess response; repeat urinalysis and basic metabolic panel

If Pharmacotherapy Initiated:

- **At 2–4 weeks after starting medication:** BP response, tolerability, renal function, and electrolytes

- **At 3 months:** ABPM to confirm target BP achievement (<130/80 mmHg per ACC/AHA; <140/90 mmHg per ESH/ESC for office BP)
- **Every 6 months:** Office BP, metabolic panel, urinalysis, urine albumin-to-creatinine ratio, medication review

Annual Surveillance:

- **ECG** — monitor for LVH regression or progression; distinguish athlete's heart (physiological eccentric hypertrophy, normal diastolic function) from hypertensive heart disease (concentric remodeling, diastolic dysfunction)
- **Echocardiogram every 2–3 years** or sooner if ECG changes — particularly important in competitive athletes to monitor LV geometry and function
- **DEXA/metabolic screening** — if corticosteroid or high-dose NSAID use suspected
- **Reassess secondary causes** if BP becomes resistant to therapy, worsens unexpectedly, or if new clinical features emerge
- **Sports participation guidance:** Stage 1 hypertension with no end-organ damage — **no restriction from competitive sport**; Stage 2 (≥160/100 mmHg) — restrict from high-static sports until controlled, per ACC/AHA and European Association of Preventive Cardiology guidelines

---

5. Patient Education — *"I'm an athlete and I eat well. Why is my BP high?"*

"I completely understand why this feels confusing — and honestly, it's one of the most common and genuinely fascinating questions in cardiovascular medicine. You're doing so many things right, and your frustration is completely valid.

Here's the honest truth: being physically active and eating well are two of the most powerful things you can do for your heart and blood vessels, and they absolutely reduce your risk compared to someone who doesn't do those things. But they don't make you immune to high blood pressure — and here's why.

Blood pressure is controlled by an extraordinarily complex system involving your kidneys, your nervous system, your hormones, the elasticity of your blood vessel walls, and your genetic blueprint. For most young people who develop high blood pressure without an obvious secondary cause, the underlying driver is a combination of genetic predisposition — meaning the way your body is wired to regulate sodium, fluid balance, and vascular tone — along with subtle environmental factors that interact with that biology.

There are also some things specific to athletes that we want to think about together. Certain types of training — particularly heavy lifting and high-intensity resistance work — can chronically elevate resting blood pressure over time. Some sports supplements, pre-workout formulas, protein powders, and energy drinks contain stimulants, high levels of caffeine, or sodium that can raise blood pressure even in healthy, fit people. And the physical and psychological stress of competitive sport, combined with disrupted sleep during training cycles, activates your stress hormone system in ways that drive blood pressure up.

None of this means you've done anything wrong. What it means is that your body has a tendency toward higher blood pressure that we've now caught early — which is actually the best possible scenario. Caught at 19, with no damage to your heart, kidneys, or eyes, we

have every opportunity to manage this with lifestyle adjustments and protect you for the long term.

We're going to work together on this. We'll look carefully at your diet — particularly sodium, which hides in places most athletes don't expect — review everything you're taking for training, make sure your exercise program is optimized for cardiovascular health, and monitor you closely. The goal is to keep you competing at your best while protecting your heart for the next 50 years."

Key Teaching Point: Never initiate lifelong antihypertensive therapy in a young patient without first performing ABPM to exclude white coat hypertension — up to 30–40% of young adults with elevated office BP have normal ambulatory readings. Additionally, always screen young male athletes tactfully but directly for anabolic steroid and stimulant use, and remember that beta-blockers are WADA-banned in precision sports — a critical prescribing consideration unique to this population.

CASE-6

Clinical Case Evaluation: Obstructive Sleep Apnea-Related Hypertension

---

1. Diagnosis & Differential Diagnosis

Most Likely Diagnosis: Obstructive Sleep Apnea (OSA) as the Primary Cause of Hypertension

This case is a near-perfect clinical portrait of OSA-driven hypertension. The convergence of obesity (BMI 34), witnessed apneic episodes by the bed partner, loud snoring, excessive daytime somnolence, and morning headaches — combined with the critically important finding of **early morning BP predominance** — constitutes a classic and highly specific phenotype. The morning hypertension pattern is the hemodynamic fingerprint of OSA: repeated nocturnal hypoxemic episodes trigger surges of sympathetic nervous system activation, catecholamine release, and renin-angiotensin-aldosterone system (RAAS) stimulation that persist into the waking hours, explaining why these patients characteristically have their highest BP readings upon awakening rather than in the evening.

OSA is now recognized by ESH/ESC 2023 and JNC guidelines as one of the most important and prevalent identifiable causes of secondary and resistant hypertension, present in up to **30–40% of hypertensive patients** and **80% of patients with truly resistant hypertension**.

Pathophysiological Cascade: Airway collapse → apnea → hypoxia + hypercapnia → arousal → massive sympathetic surge → catecholamine release → vasoconstriction and tachycardia → BP spike → repeated hundreds of times nightly → sustained sympathetic overdrive → structural vascular remodeling → chronic daytime hypertension → loss of normal nocturnal BP dipping → non-dipper or reverse-dipper ABPM pattern

Differential Diagnoses:

- **Primary (Essential) Hypertension** — most common hypertension diagnosis overall; however, the clinical context here strongly implicates OSA as the driving etiology rather than idiopathic primary hypertension; both can coexist and frequently do

- **Primary Hyperaldosteronism** — highly prevalent in OSA patients (hypoxia stimulates aldosterone secretion independently); the two conditions are biologically intertwined and co-occur far more than previously recognized; ARR should be checked if BP remains resistant after OSA treatment
- **Pheochromocytoma** — nocturnal hypertensive surges and morning headaches can overlap; however, absence of diaphoresis, palpitations, and paroxysmal episodes makes this less likely; metanephrines can be checked if clinical suspicion warrants
- **Cushing's Syndrome** — central obesity, hypertension, and metabolic derangement overlap with OSA; look for moon face, purple striae, proximal myopathy, and hyperglycemia; 24-hour urinary cortisol or overnight dexamethasone suppression test if suspected
- **Hypothyroidism** — causes weight gain, fatigue, and contributes to upper airway muscle laxity, worsening OSA; TSH should always be checked in new OSA diagnosis
- **Resistant Hypertension from any cause** — OSA is the most common reversible contributor to apparent treatment-resistant hypertension and should be systematically sought in all resistant hypertension workups
- **Central Sleep Apnea** — apnea without obstructive mechanism; associated with heart failure, stroke, or opioid use; differentiated on polysomnography by absence of respiratory effort during apneic episodes; different treatment pathway (ASV therapy rather than CPAP)
- **Obesity Hypoventilation Syndrome (Pickwickian Syndrome)** — chronic daytime hypercapnia in obese patients; can coexist with OSA; diagnosed by arterial blood gas showing elevated $PaCO_2$ while awake

---

2. Diagnostic Workup

Gold Standard Confirmatory Test:

In-Laboratory Attended Polysomnography (PSG)

Full overnight polysomnography in a sleep laboratory is the definitive gold standard for OSA diagnosis and severity classification. It simultaneously records:

- **Electroencephalography (EEG)** — sleep staging and arousal detection
- **Electromyography (EMG)** — chin and limb muscle activity
- **Electrooculography (EOG)** — eye movement for REM identification
- **Airflow** — nasal pressure transducer and thermistor
- **Respiratory effort** — thoracic and abdominal inductance plethysmography belts (distinguishes obstructive from central apnea)
- **Oxygen saturation (SpO_2)** — continuous pulse oximetry
- **Heart rate and ECG**
- **Body position** — positional OSA identification
- **Leg movements** — exclude periodic limb movement disorder

Apnea-Hypopnea Index (AHI) Classification:

- Mild OSA: AHI 5–14 events/hour
- Moderate OSA: AHI 15–29 events/hour
- Severe OSA: AHI ≥30 events/hour

Home Sleep Apnea Testing (HSAT): An acceptable, cost-effective alternative for patients with **high pre-test probability of moderate-to-severe uncomplicated OSA** (like this patient) per AASM guidelines. Records airflow, respiratory effort, and oximetry but does not capture sleep staging. A negative or inconclusive HSAT in a symptomatic patient must be followed by full in-laboratory PSG.

Additional Recommended Workup:

- **ABPM (24-hour ambulatory BP monitoring)** — characterize non-dipping or reverse-dipping nocturnal pattern; quantify BP load; essential for hypertension phenotyping in OSA
- **TSH** — exclude hypothyroidism contributing to both OSA and hypertension
- **Fasting glucose, HbA1c, fasting lipid panel** — OSA is independently associated with insulin resistance and metabolic syndrome; cardiovascular risk stratification
- **Serum electrolytes, creatinine, eGFR, urinalysis** — assess end-organ renal involvement
- **ECG** — assess for LVH, atrial fibrillation (OSA is the most common reversible cause of AF), conduction disease
- **Aldosterone-to-renin ratio (ARR)** — if BP remains poorly controlled after CPAP initiation; co-existing primary hyperaldosteronism is present in up to 30% of OSA patients
- **Echocardiography** — if ECG abnormal or clinical concern for pulmonary hypertension (OSA-related) or LV dysfunction; assess diastolic function, RV pressure, and LV geometry

---

3. Management Plan

First-Line Treatment: Continuous Positive Airway Pressure (CPAP) Therapy

CPAP is the cornerstone treatment for moderate-to-severe OSA and carries the strongest evidence for both symptom relief and cardiovascular risk reduction, including meaningful antihypertensive effects.

Mechanism of BP Lowering by CPAP:

- Eliminates apnea-induced sympathetic surges
- Reduces nocturnal catecholamine release
- Restores normal nocturnal BP dipping pattern
- Reduces systemic inflammation (CRP, IL-6) and oxidative stress
- Decreases aldosterone secretion driven by chronic intermittent hypoxia

- Improves endothelial function and arterial compliance

Expected BP Reduction with CPAP: Meta-analyses consistently show systolic BP reduction of **3–10 mmHg** — most pronounced in patients with severe OSA, resistant hypertension, high CPAP compliance (>4 hours/night), and daytime sleepiness. While modest, this reduction is clinically significant at a population level and reduces cardiovascular event risk.

CPAP Prescription Parameters:

- **Auto-titrating CPAP (APAP)** preferred for initial therapy — self-adjusts pressure breath-by-breath; superior comfort and adherence vs. fixed pressure
- Pressure range typically 4–20 cmH2O; titrated based on residual AHI data downloaded from device
- Heated humidifier — mandatory to reduce mucosal dryness and improve adherence
- Mask interface selection — nasal pillow, nasal mask, or full face mask based on patient anatomy and mouth breathing tendency; mask fit is the single most important determinant of adherence

Adjunctive and Complementary Interventions:

Weight loss — the single most impactful disease-modifying intervention in obese OSA patients; 10% body weight reduction can reduce AHI by ~26%; bariatric surgery in morbid obesity can achieve near-complete OSA resolution in selected patients; however, weight loss alone is insufficient for moderate-to-severe OSA and should never replace CPAP

Positional therapy — if PSG confirms predominant supine OSA; positional devices or sleep position training as adjunct

Upper airway muscle training (Myofunctional therapy) — emerging evidence; reduces AHI by ~50% in mild-to-moderate OSA; useful adjunct

Mandibular Advancement Device (MAD) — indicated for mild-to-moderate OSA or CPAP-intolerant patients; less effective than CPAP for severe OSA but superior adherence in some patients

Surgical options:

- **Uvulopalatopharyngoplasty (UPPP)** — moderate evidence; reserved for CPAP-intolerant patients with specific upper airway anatomy
- **Hypoglossal nerve stimulation (Inspire therapy)** — highly effective FDA-approved implantable device for moderate-to-severe OSA in CPAP-intolerant patients; AHI 15–65, BMI <35, no concentric palatal collapse on drug-induced sleep endoscopy; growing evidence base
- **Bariatric surgery** — for BMI ≥35 with obesity-related comorbidities including OSA; highly effective for weight-driven disease

Antihypertensive Pharmacotherapy:

- Initiate or optimize antihypertensive therapy concurrently — do not delay treatment waiting for CPAP response

- **Aldosterone antagonists (spironolactone/eplerenone)** — particularly effective in OSA-related hypertension given the RAAS activation and co-existing hyperaldosteronism component; evidence supports superior BP reduction vs. other agents in this phenotype
- **ACE inhibitors or ARBs** — first-line for renoprotection and RAAS blockade
- **Calcium channel blockers** — excellent adjunct; no metabolic concerns
- Target BP <130/80 mmHg per ACC/AHA 2017; reassess antihypertensive requirements after 3 months of effective CPAP therapy as medication doses may be reducible

---

4. Long-term Follow-up Protocol

Short-Term (First 3 Months — Critical Adherence Window):

- **At 2–4 weeks:** CPAP device data download — assess average nightly usage hours, residual AHI, mask leak data, pressure adequacy; troubleshoot mask fit, pressure comfort, and side effects; this visit is the most important for long-term adherence success
- **At 6–8 weeks:** BP reassessment (office and HBPM diary); symptomatic response (Epworth Sleepiness Scale); weight, metabolic panel
- **At 3 months:** Repeat ABPM — assess restoration of nocturnal dipping, BP load reduction; CPAP compliance review; fasting glucose, HbA1c, lipid panel; reassess antihypertensive regimen

Medium-Term (3–12 Months):

- **Every 3 months:** CPAP data review, BP monitoring, weight tracking, metabolic parameters
- **At 6 months:** Repeat sleep study (HSAT or PSG) if significant weight loss achieved (>10%) to reassess AHI and determine if CPAP pressure adjustment or therapy discontinuation trial is appropriate
- **Cardiology or hypertension specialist referral** if BP remains >140/90 mmHg despite CPAP adherence and dual antihypertensive therapy — evaluate for co-existing primary hyperaldosteronism

Annual Surveillance:

- **CPAP device data review** — adherence, residual AHI, equipment maintenance (mask replacement every 3–6 months, filters monthly)
- **Full metabolic panel** — glucose, HbA1c, lipid panel, renal function
- **BP assessment with ABPM** every 1–2 years — monitor nocturnal dipping restoration and sustained BP control
- **ECG annually** — monitor for atrial fibrillation (OSA is the most common reversible AF trigger), LVH regression
- **Echocardiogram every 3–5 years** — LV diastolic function, pulmonary artery pressure, RV function

- **Weight and BMI at every visit** — central to disease trajectory
- **Repeat PSG** if: symptoms recur despite CPAP, significant weight change (gain or loss >10%), CPAP residual AHI persistently elevated, or therapy change considered

---

5. Patient Education — *"Do I really have to wear this mask every night?"*

"I really appreciate you asking that directly — because it tells me you're thinking seriously about this, and that matters enormously for your health outcomes. And I want to give you a completely straight answer: yes, ideally every night. But let me explain exactly why, because I think once you understand what's happening in your body while you sleep, this will make a lot more sense.

Every time you stop breathing at night — and based on your symptoms, this is likely happening dozens, possibly hundreds of times — your brain detects the drop in oxygen and triggers an emergency alarm response. Your body floods with stress hormones, your heart rate surges, your blood vessels clamp down, and your blood pressure spikes. Then you start breathing again, and it settles — until the next episode, minutes later. This cycle repeats all night, every night.

The reason your blood pressure is highest in the morning is precisely because your body has been running this emergency stress response for six to eight hours while you were asleep. Your cardiovascular system never gets to rest. And over months and years, this relentless overnight strain damages the walls of your blood vessels, strains your heart, disrupts your heart rhythm, and significantly raises your risk of heart attack, stroke, and heart failure.

The CPAP mask stops all of that. It gently keeps your airway open so the apneas don't happen, the oxygen stays stable, the stress hormones don't surge, and your heart and blood vessels finally get the overnight recovery they need. Many patients see their morning blood pressure improve meaningfully within weeks — some are eventually able to reduce their blood pressure medications.

I won't pretend the mask feels natural at first — it doesn't for most people. But here is what the evidence tells us: the patients who struggle most in the first two to four weeks often become the most committed long-term users once they feel the difference — better sleep, clearer thinking, more energy, lower blood pressure, and reduced cardiovascular risk.

Modern CPAP machines are quieter, lighter, and more comfortable than ever before. We will work closely with you to find the right mask, the right settings, and the right support to make this work for your life. The first month is the hardest. Let's get through it together — your heart is counting on it."

Key Teaching Point: OSA is the most common reversible cause of resistant hypertension and the leading reversible trigger of atrial fibrillation. The morning hypertension pattern and non-dipping ABPM profile are its hemodynamic signatures. Always check ARR after CPAP initiation if BP remains resistant — co-existing primary hyperaldosteronism is present in up to 30% of OSA patients and is frequently unmasked only after sleep-disordered breathing is treated.

CASE-7

Clinical Case Evaluation: Hypertension with Upper-Lower Extremity BP Gradient in a Young Male

---

1. Diagnosis & Differential Diagnosis

Most Likely Diagnosis: Coarctation of the Aorta (CoA)

This case is a textbook presentation of aortic coarctation delivering its cardinal clinical triad with surgical precision. The **upper extremity hypertension with lower extremity hypotension** (pressure gradient >20 mmHg between arms and legs), **interscapular continuous murmur** from collateral vessel flow, and **exertional leg fatigue** from reduced lower limb perfusion during exercise in an 18-year-old male constitute one of the most recognizable diagnostic pictures in cardiovascular medicine. The continuous murmur heard between the scapulae represents blood flowing through intercostal collateral arteries that have developed over years to bypass the obstruction — a testament to the body's remarkable but ultimately insufficient compensatory vascular remodeling.

Coarctation most commonly occurs **just distal to the left subclavian artery** at the level of the ligamentum arteriosum (juxtaductal position), accounting for >95% of cases. The resulting mechanical obstruction creates a pressure compartment — high pressure proximal (upper body, arms, brain) and relatively low pressure distal (lower body, legs, kidneys) — with profoundly different physiological consequences in each territory.

Pathophysiological Basis of Hypertension in CoA: Hypertension in coarctation is not purely mechanical. It involves three interacting mechanisms: direct pressure elevation proximal to the obstruction, activation of the RAAS by relative renal hypoperfusion (the kidneys "sense" reduced perfusion despite normal or even elevated proximal BP), and increased sympathetic nervous system activity — explaining why hypertension frequently persists or recurs even after anatomically successful repair.

Differential Diagnoses:

- **Takayasu Arteritis** — large vessel vasculitis causing aortic and branch vessel stenosis; typically affects young Asian women aged 10–40; associated with constitutional symptoms (fever, malaise, weight loss), elevated ESR/CRP, and absent or diminished upper extremity pulses; can cause aortic narrowing mimicking coarctation anatomically but distinguished by inflammatory markers, vessel wall thickening on MRI/CT angiography, and systemic inflammatory features
- **Essential Hypertension** — by far the most common cause of hypertension overall but should never be diagnosed in a young patient without first excluding secondary causes; the striking BP differential and murmur make this diagnosis untenable here
- **Renal Artery Stenosis** — causes secondary hypertension via RAAS activation; no upper-lower extremity gradient; no murmur between scapulae; abdominal bruit may be present
- **Williams Syndrome** — genetic disorder (ELN gene deletion) causing supravalvular aortic stenosis and peripheral pulmonary artery stenosis; elfin facies, hypercalcemia, intellectual disability; different murmur location and character
- **Middle Aortic Syndrome** — rare segmental narrowing of the descending thoracic or abdominal aorta; can mimic coarctation hemodynamically; occurs in children and

young adults; associated with neurofibromatosis, Williams syndrome, or Takayasu arteritis

- **Interrupted Aortic Arch** — complete discontinuity of the aortic arch; typically presents in neonates with ductal-dependent circulation; incompatible with survival to age 18 without prior intervention unless partial
- **Subclavian Steal Syndrome** — proximal subclavian artery stenosis causing arm BP discrepancy; neurological symptoms with arm exercise; no lower extremity gradient

---

2. Diagnostic Workup

Initial Imaging Test of Choice: Transthoracic Echocardiography (TTE) with Doppler

Echocardiography is the first-line imaging modality in suspected coarctation, providing:

- **Direct visualization** of the coarctation site, particularly in children and young adults with good acoustic windows
- **Continuous wave Doppler** across the coarctation — peak gradient measurement; a **mean gradient >20 mmHg** at rest is hemodynamically significant; the characteristic **"diastolic tail" or saw-tooth pattern** (persistent diastolic forward flow) is pathognomonic of significant coarctation
- **Assessment of associated cardiac anomalies** — critically important: **bicuspid aortic valve (BAV)** is present in **50–85% of coarctation patients**; ventricular septal defect, mitral valve abnormalities, and hypoplastic left heart spectrum must be systematically excluded
- **LV hypertrophy and function** — quantify hypertensive cardiac remodeling
- **Aortic root and ascending aorta dimensions** — BAV-associated aortopathy causes progressive dilatation independent of valve function

Chest X-Ray (CXR) — Classic Signs:

While not the primary diagnostic tool, CXR findings are historically important and clinically recognizable:

- **"Figure 3 sign"** — the most classic radiographic sign; the upper curve of the "3" represents the dilated aortic knuckle and left subclavian artery proximal to the coarctation, while the lower curve represents post-stenotic dilatation of the descending aorta; the indentation between them marks the coarctation itself
- **"Reverse 3 sign" (E sign)** on barium swallow — indentation of the esophagus at the coarctation level
- **Rib notching (inferior rib scalloping)** — the most specific CXR finding; bilateral notching of the posterior inferior aspects of ribs 3–8 caused by tortuous, enlarged intercostal collateral arteries eroding the rib undersurfaces over years; typically appears after age 6–7 and becomes progressively more pronounced; its presence confirms long-standing, hemodynamically significant coarctation
- **Cardiomegaly** — LV enlargement from chronic pressure overload
- **Prominent left heart border** — LV hypertrophy pattern

Definitive Anatomical Imaging:

- **CT Angiography (CTA) of the aorta** — gold standard for pre-interventional planning; provides precise anatomical detail of coarctation length, location, diameter, tortuosity, relationship to arch vessels, and extent of collateral circulation; 3D reconstruction guides intervention strategy
- **MRI/MR Angiography** — preferred in younger patients to avoid radiation; equivalent anatomical detail; additionally provides 4D flow mapping, collateral quantification, and myocardial tissue characterization; the **gold standard for post-repair surveillance** due to zero radiation exposure over lifetime follow-up
- **Cardiac catheterization with angiography** — reserved for cases where non-invasive imaging is insufficient or when simultaneous intervention is planned; provides hemodynamic pressure gradient measurement across the coarctation under physiological conditions

---

3. Management Plan

Definitive Management: Repair of Coarctation

The indication for repair is met when **peak-to-peak catheterization gradient >20 mmHg**, or **mean Doppler gradient >20 mmHg**, or significant coarctation with upper-lower extremity BP differential >20 mmHg with radiological evidence of collateral flow — all present in this patient. At age 18, repair should not be delayed.

Option A: Percutaneous Catheter-Based Intervention (Preferred in Adolescents and Adults)

Balloon angioplasty with stent implantation is the treatment of choice for native discrete coarctation in adolescents and adults per ACC/AHA Congenital Heart Disease Guidelines 2018:

- Covered stents preferred — prevent aortic wall injury, contain dissection risk, allow future re-dilation as patient grows
- Performed under general anesthesia with intravascular ultrasound and pressure wire guidance
- Immediate gradient abolition in >95% of cases
- Advantages: no thoracotomy, shorter recovery, excellent results in discrete juxtaductal coarctation
- Complications: aortic dissection, stent migration, restenosis (~10% at 5 years), paradoxical hypertension post-procedure (requires anticipatory beta-blockade)

Option B: Surgical Repair (Preferred for Complex Anatomy)

Indicated for: long-segment coarctation, hypoplastic aortic arch, coarctation in infants, associated cardiac defects requiring simultaneous repair, or failed/unsuitable percutaneous approach:

- **Resection with end-to-end anastomosis** — preferred for discrete coarctation; lowest restenosis rate

- **Extended end-to-end anastomosis** — for arch hypoplasia
- **Subclavian flap aortoplasty** — historically used in infants; sacrifices left subclavian artery
- **Patch aortoplasty** — largely abandoned due to high late aneurysm formation risk
- **Interposition graft** — for long-segment coarctation; prosthetic graft material

Perioperative Management:

- **Beta-blockade** (esmolol IV intraoperatively; oral beta-blocker post-procedure) — prevents paradoxical post-repair hypertension, which can cause hypertensive encephalopathy, aortic dissection, or mesenteric arteritis in the immediate post-repair period
- **Spinal cord protection** during surgery — motor evoked potential monitoring; minimize aortic cross-clamp time; left heart bypass in complex cases
- **Neurological monitoring** — paradoxical hypertension and hyperperfusion injury risk in the early post-repair period

Management of Associated Bicuspid Aortic Valve:

- Echocardiographic surveillance of BAV function and aortic root dimensions at baseline and annually — BAV-associated aortopathy progresses independently of coarctation repair status
- Aortic root surgery if diameter ≥5.0 cm (or ≥4.5 cm with rapid progression or family history of dissection) per current guidelines

---

4. Long-term Follow-up Protocol

This is lifelong. Coarctation repair is not a cure — it is the beginning of a lifetime of structured surveillance.

Immediate Post-Repair (First 72 Hours):

- Continuous intra-arterial BP monitoring — detect and treat post-repair paradoxical hypertension aggressively
- Neurological assessment — spinal cord ischemia, cerebral hyperperfusion
- Pulse oximetry and bilateral limb BP measurement — confirm gradient abolition
- Echocardiography before discharge — residual gradient, LV function, pericardial effusion

At 1 Month:

- Office BP both arms and one leg — confirm gradient elimination
- Echocardiogram — residual gradient assessment, LV remodeling
- Wound/access site assessment

At 3 and 6 Months:

- BP assessment and ABPM — detect exercise hypertension or masked residual hypertension; up to 30% of patients have normal resting BP but pathological BP response to exercise post-repair
- **Exercise stress test with BP monitoring** — exaggerated BP response to exercise (systolic BP >210 mmHg) is an independent predictor of late cardiovascular events even with successful repair
- Echocardiogram — LV hypertrophy regression, gradient surveillance

Annual Lifelong Surveillance:

- **Office BP bilaterally and ABPM** — hypertension persists or recurs in 25–68% of repaired patients depending on age at repair; earlier repair = lower recurrence risk
- **MRI/MRA of the entire aorta** — every **3–5 years** as minimum; detect restenosis at repair site, aortic aneurysm formation (occurs in 5–10% post-surgical repair at anastomosis site), aortic root dilatation from BAV-associated aortopathy, and new aneurysms in the collateral vessels
- **Echocardiogram annually** — BAV surveillance (stenosis, regurgitation progression), aortic root dimensions, LV function and geometry
- **Exercise stress test with BP measurement** — every 2–3 years; critical for detecting exercise-induced hypertension which predicts late cerebrovascular events
- **Neurovascular assessment** — intracranial aneurysms (Circle of Willis) occur in 10% of CoA patients (associated with connective tissue abnormality); MRA of the brain at baseline and if headaches develop; berry aneurysm rupture is a recognized cause of death in this population
- **Genetic counseling and family screening** — CoA and BAV cluster in families; first-degree relatives should undergo echocardiographic screening; evaluate for associated syndromes (Turner syndrome in females — 45,X; NOTCH1, GATA5 mutations)
- **Contraception and pregnancy counseling in females** — pregnancy in unrepaired or residual CoA carries very high maternal cardiovascular risk; pre-pregnancy multidisciplinary assessment mandatory; delivery in specialized congenital heart disease center

---

5. Patient Education — *"Is my heart fixed now that the narrowing is gone?"*

"I am so glad you asked that question — because it gives me the opportunity to have one of the most important conversations we will have together. And I want to be completely honest with you, because you deserve to understand your body and your future.

The short answer is: the procedure was a critical and necessary step, and it went well — but it is not a complete cure, and your heart and aorta will need attention for the rest of your life. Let me explain why, because this is not meant to worry you — it's meant to empower you.

First, the good news: by fixing the narrowing, we have removed the main mechanical obstacle that was overworking your heart and starving your legs of blood flow. Your heart no longer has to pump against that resistance every single beat of every single day. Over the coming months, we expect your blood pressure to improve, your leg fatigue during soccer to

resolve, and your heart muscle to gradually normalize. That is a genuinely significant and life-changing improvement.

But here is what we also know from decades of following patients just like you. The high blood pressure that built up over the years before your repair caused changes in your blood vessel walls and heart muscle that don't simply disappear overnight. Some patients continue to have blood pressure that runs a little high — especially during exercise — even after a perfect repair. That is why we will monitor your blood pressure carefully, including during an exercise test, for years to come.

Second, about half the people born with aortic coarctation also have a bicuspid aortic valve — meaning the main valve in your heart has two leaflets instead of the normal three. We need to watch that valve over time because it can gradually become stiff or leaky, and the aorta connected to it can slowly enlarge. We will check this with ultrasound of your heart every year.

Third, the aorta at the repair site needs surveillance with MRI scans every few years to make sure it remains wide open and has not developed any abnormal bulging.

And finally — this is important for your whole family — this condition can run in families. Your parents, siblings, and one day your own children should have their hearts checked.

So to answer your question directly: your heart is significantly better, and the hardest part is behind you. But 'fixed' in the sense of 'never needs attention again' — that would not be accurate, and I would be doing you a disservice to say otherwise. Think of it this way: you now have a heart that needs a good doctor and annual check-ins, but one that can absolutely support a full, active, long, and healthy life — including soccer."

Key Teaching Point: Coarctation of the aorta is never truly cured by repair — it is managed. Late hypertension, restenosis, BAV-related aortopathy, aortic aneurysm at the repair site, and intracranial aneurysms are well-documented long-term complications requiring structured lifelong surveillance. Age at repair is the single most important determinant of late cardiovascular outcomes — earlier repair yields better long-term BP control and lower cardiovascular mortality.

CASE-8

Clinical Case Evaluation: Rapid Weight Gain, Hypertension, and Purple Striae in a Young Woman

---

1. Diagnosis & Differential Diagnosis

Most Likely Diagnosis: Endogenous Cushing's Syndrome

This case assembles the hallmark features of hypercortisolism with remarkable clarity. The combination of **centripetal obesity** (face and abdomen predominantly), **wide violaceous striae** (>1 cm wide purple stretch marks — a highly specific feature of cortisol excess distinguishing them from the narrow white striae of simple obesity or pregnancy), **easy bruising** from cortisol-induced collagen degradation and capillary fragility, and **hypertension** in a 38-year-old woman without exogenous steroid exposure constitutes a compelling clinical phenotype of endogenous Cushing's syndrome until proven otherwise.

The pathophysiology is driven by chronic cortisol excess acting on multiple organ systems simultaneously: mineralocorticoid receptor activation causing sodium retention and hypertension, proteolysis of skin and connective tissue causing striae and bruising, redistribution of adipose tissue to visceral and facial depots (moon face, buffalo hump, supraclavicular fat pads), suppression of gonadal and growth hormone axes, and insulin resistance contributing to metabolic syndrome.

Etiology Classification of Endogenous Cushing's Syndrome:

ACTH-Dependent (80% of cases):

- **Cushing's Disease** (pituitary corticotroph adenoma) — most common cause overall, accounting for ~70% of endogenous cases; typically microadenoma (<10mm); more common in women aged 20–50; ACTH drives bilateral adrenal hyperplasia
- **Ectopic ACTH Syndrome** — ~10% of cases; ACTH secreted by non-pituitary tumor; small cell lung carcinoma (most common), bronchial carcinoid, thymic carcinoid, pancreatic neuroendocrine tumor, medullary thyroid carcinoma; often presents with more severe, rapidly progressive hypercortisolism, hypokalemia, hyperpigmentation, and less classic cushingoid features due to speed of onset

ACTH-Independent (20% of cases):

- **Adrenal adenoma** — benign unilateral cortisol-secreting tumor; suppresses ACTH; well-defined on CT; surgically curable
- **Adrenal carcinoma** — rare but aggressive; large heterogeneous adrenal mass; often secretes multiple steroids (cortisol, androgens, aldosterone precursors); poor prognosis
- **Bilateral adrenal hyperplasia variants** — Primary Pigmented Nodular Adrenocortical Disease (PPNAD, associated with Carney Complex); ACTH-Independent Macronodular Adrenal Hyperplasia (AIMAH/PBMAH)

Differential Diagnoses (Pseudo-Cushing States — Must Exclude):

- **Obesity with metabolic syndrome** — can cause mild hypercortisoluria but striae are typically narrow and white; dexamethasone suppression is usually normal
- **Alcoholic pseudo-Cushing** — chronic heavy alcohol intake causes clinical and biochemical features indistinguishable from Cushing's; alcohol history essential; resolves with abstinence
- **Major depressive disorder** — activates HPA axis; can cause mild cortisol elevation and abnormal dexamethasone suppression; psychiatric evaluation essential
- **Polycystic Ovary Syndrome (PCOS)** — shares features of weight gain, hirsutism, menstrual irregularity; normal cortisol biochemistry
- **Exogenous/Factitious steroid use** — must be meticulously excluded including topical, inhaled, intra-articular, and herbal preparations containing glucocorticoids; plasma ACTH will be suppressed and endogenous cortisol low

---

2. Diagnostic Workup

Screening Tests — Three Validated First-Line Options (Endocrine Society Guidelines 2008, Updated 2015):

Any ONE of the following is appropriate as initial screening; two abnormal tests from different modalities are required to confirm the diagnosis:

Option 1: Late-Night Salivary Cortisol (LNSC) — Preferred Initial Screen

- Collected at 11 PM–midnight on two separate nights
- Exploits the loss of normal circadian cortisol nadir in Cushing's syndrome
- Cortisol should be at its lowest point at midnight; elevation above laboratory threshold is highly abnormal
- Sensitivity ~92–100%, Specificity ~93–100%
- Non-invasive, outpatient, patient-collected; ideal for initial screening
- Falsely elevated by: shift work, night shift employment, acute illness, smoking, licorice ingestion

Option 2: 24-Hour Urinary Free Cortisol (UFC)

- Collected on two separate occasions (day-to-day variability necessitates duplicate collection)
- Measures integrated cortisol production over 24 hours
- Must confirm complete collection with urinary creatinine measurement
- Sensitivity ~95%, Specificity ~98% when >3–4× upper limit of normal
- Less useful in mild Cushing's; falsely elevated by high fluid intake (>5L/day) and renal tubular abnormalities

Option 3: 1mg Overnight Dexamethasone Suppression Test (ODST)

- 1mg dexamethasone orally at 11 PM; serum cortisol measured at 8 AM next morning
- Normal suppression: cortisol <1.8 mcg/dL
- Failure to suppress confirms autonomous cortisol secretion
- High sensitivity (~95%) but lower specificity (~80%); false positives with: CBG elevation (estrogen use, pregnancy), CYP3A4 inducers (phenytoin, rifampicin, carbamazepine) increasing dexamethasone metabolism, depression, alcoholism, obesity

Confirmatory Testing (after two abnormal screens):

2-Day Low-Dose Dexamethasone Suppression Test (LDDST)

- Dexamethasone 0.5mg every 6 hours for 48 hours; cortisol measured 6 hours after final dose
- Failure to suppress cortisol <1.8 mcg/dL confirms Cushing's syndrome
- Gold standard biochemical confirmation test

Localization and Etiology Workup (after biochemical confirmation):

Step 1 — Plasma ACTH measurement:

- **ACTH suppressed (<5 pg/mL)** → ACTH-independent → adrenal source → **adrenal CT**
- **ACTH elevated or inappropriately normal (>15–20 pg/mL)** → ACTH-dependent → pituitary vs. ectopic

Step 2 — For ACTH-dependent disease:

- **Pituitary MRI with gadolinium (dynamic protocol)** — first-line; identifies adenoma in only ~50–60% of Cushing's disease cases due to microadenoma size; normal MRI does NOT exclude pituitary source
- **High-Dose Dexamethasone Suppression Test (HDDST)** — 8mg overnight; >50% suppression of cortisol suggests pituitary source (Cushing's disease); no suppression suggests ectopic; imperfect discriminator used adjunctively
- **CRH stimulation test** — exaggerated ACTH and cortisol response suggests pituitary adenoma; blunted response suggests ectopic source

Gold Standard for Distinguishing Cushing's Disease from Ectopic ACTH: Bilateral Inferior Petrosal Sinus Sampling (BIPSS)

- Simultaneous sampling from both inferior petrosal sinuses (draining the pituitary) and peripheral vein before and after CRH stimulation
- Central-to-peripheral ACTH gradient ≥2 (basal) or ≥3 (post-CRH) confirms pituitary source with sensitivity ~95%, specificity ~95%
- Additionally provides **lateralization** of the adenoma within the pituitary gland to guide surgical approach
- Indicated when pituitary MRI is negative or equivocal, or when ectopic source cannot be excluded clinically

For Ectopic ACTH — Tumor Search:

- **CT chest/abdomen/pelvis** with contrast — first-line for occult ectopic source
- **^{18}F-FDG PET/CT or ^{68}Ga-DOTATATE PET/CT** — superior for well-differentiated neuroendocrine tumors (bronchial carcinoid); highly recommended when CT is negative
- **Octreotide scintigraphy** — for somatostatin receptor-expressing tumors

---

3. Medical Management When No Tumor Can Be Found

When the source of ACTH or cortisol excess cannot be identified or localized despite complete workup, or when surgery has failed, is not feasible, or the patient is awaiting surgery, medical therapy targets cortisol synthesis or action:

Category 1 — Steroidogenesis Inhibitors (Adrenal-Directed):

Metyrapone — inhibits 11β-hydroxylase (final step in cortisol synthesis); rapid onset of action within hours; first-line in many centers for urgent cortisol reduction; oral administration;

side effects: hirsutism/acne (androgen precursor accumulation), hypokalemia, hypertension; dose 500mg–6g/day divided doses; monitor with UFC or serum cortisol

Ketoconazole — antifungal with potent adrenal steroidogenesis inhibition (multiple enzymatic steps); widely used historically; oral; side effects: hepatotoxicity (requires LFT monitoring), drug interactions via CYP3A4; dose 200–1200mg/day; less favored in newer guidelines but still widely used

Osilodrostat — potent 11β-hydroxylase inhibitor; FDA-approved (2020) specifically for Cushing's disease; more selective than metyrapone with favorable side effect profile; dose 2–30mg twice daily; rapidly becoming preferred first-line steroidogenesis inhibitor

Mitotane — adrenolytic agent (causes adrenal cortical destruction) and steroidogenesis inhibitor; primarily used for adrenocortical carcinoma; used in persistent/recurrent Cushing's at lower doses; narrow therapeutic window; significant toxicity (GI, neurological); requires mitotane serum level monitoring

Category 2 — Pituitary-Directed (ACTH-Targeted):

Pasireotide — somatostatin analogue with high affinity for SST5 receptor (predominant in corticotroph adenomas); FDA-approved for Cushing's disease; subcutaneous injection twice daily or monthly LAR formulation; reduces ACTH and cortisol in ~25–40% of patients; major side effect: **hyperglycemia** (occurs in up to 75% of patients — requires glucose monitoring and often insulin); dose 0.6–0.9mg SC twice daily

Cabergoline — dopamine D2 receptor agonist; ~30–40% of corticotroph adenomas express D2 receptors; oral weekly dosing; modest efficacy but favorable tolerability and cost profile; often used as adjunct or in mild disease

Category 3 — Glucocorticoid Receptor Antagonist:

Mifepristone (RU-486) — blocks glucocorticoid receptor peripherally; FDA-approved for hyperglycemia associated with Cushing's syndrome; does NOT lower cortisol levels (which rise further due to loss of negative feedback — ACTH and cortisol rise on treatment); clinical and metabolic improvement despite rising cortisol; cannot use cortisol as monitoring parameter — use clinical response and glucose; significant drug interactions; risk of adrenal insufficiency during illness; dose 300–1200mg/day

Bilateral Adrenalectomy — When Medical Therapy Fails: When no tumor is found after exhaustive workup and medical therapy is inadequate or poorly tolerated, **bilateral laparoscopic adrenalectomy** achieves immediate and definitive cortisol normalization. Trade-off: permanent adrenal insufficiency requiring lifelong glucocorticoid and mineralocorticoid replacement. Risk of **Nelson's Syndrome** (aggressive growth of the underlying corticotroph adenoma after removal of cortisol feedback) — occurs in 15–25%; requires lifelong pituitary MRI surveillance post-adrenalectomy; prophylactic pituitary radiation may reduce risk.

---

4. Long-term Follow-up Protocol

Post-Surgical Remission (Transsphenoidal Surgery for Cushing's Disease):

Immediate Post-op (Days 1–7):

- Expect **post-surgical adrenal insufficiency** — the hallmark of surgical remission and biochemical cure; suppressed normal corticotrophs require months to recover

- Hydrocortisone replacement initiated immediately post-op (10–15mg/m^2/day in divided doses)
- Morning serum cortisol on post-op day 1–3 (before hydrocortisone): cortisol <2 mcg/dL = remission; >5 mcg/dL = persistent disease
- Monitor for hyponatremia (post-op SIADH is common after transsphenoidal surgery)
- Diabetes insipidus monitoring (urine output, serum sodium, osmolality)

At 6–12 Weeks:

- Full biochemical reassessment: 24-hour UFC, late-night salivary cortisol, LDDST
- Pituitary hormone panel: ACTH, morning cortisol (off hydrocortisone for 18–24 hours if safe), IGF-1, prolactin, LH/FSH, TSH/FT4, testosterone/estradiol
- Assess adrenal recovery — formal cortisol stimulation test (250mcg Synacthen) to determine if replacement can be weaned

At 6 Months and 1 Year:

- Repeat pituitary MRI — baseline post-op imaging; compare to pre-op for residual tumor
- Full hormonal reassessment; taper hydrocortisone if adrenal recovery confirmed
- DEXA scan — Cushing's causes significant osteoporosis; reassess bone density and initiate bisphosphonate if T-score ≤−2.5
- Metabolic panel, BP, glucose, lipid profile — cardiovascular risk factor reassessment; many normalize with cure

Annual Lifelong Surveillance:

- **Late-night salivary cortisol or 24-hour UFC** — recurrence occurs in 15–25% within 10 years; annual biochemical screening mandatory indefinitely
- **Pituitary MRI** every 1–2 years for first 5 years; then every 3–5 years if stable
- **DEXA scan** every 2 years — monitor bone density recovery (significant improvement expected over 2–5 years post-cure)
- **Cardiovascular risk factor monitoring** — BP, fasting glucose/HbA1c, lipid panel, weight; many but not all cardiovascular risk factors normalize after cure; residual excess cardiovascular mortality persists for years post-remission
- **Neuropsychological assessment** — depression, anxiety, and cognitive impairment are common post-cure and may persist; refer to psychiatry/psychology as needed
- **For Nelson's Syndrome (post-bilateral adrenalectomy):** Pituitary MRI every 6–12 months for first 5 years, annually thereafter; plasma ACTH levels serially — rising ACTH precedes radiological progression

---

5. Patient Education — *"Will these stretch marks and the 'moon face' ever go away?"*

"Thank you so much for asking this — because I know these physical changes have been one of the most distressing aspects of this illness, and you deserve a thoughtful and honest answer.

Let me start with the encouraging part, because there genuinely is a lot of hope here.

The changes to your face — the roundness, the fullness in your cheeks, and the fat deposits that have accumulated — are caused by the excess cortisol redistributing where your body stores fat. Once we successfully treat the underlying cause and your cortisol levels return to normal, that fat redistribution gradually reverses. Most patients see meaningful improvement in their facial appearance within ***3 to 12 months*** *after successful treatment. The moon face is one of the features that tends to respond most visibly and most gratifyingly. Many of my patients have told me it was the moment they started recognizing themselves in the mirror again — and that is a powerful thing.*

The abdominal fat also improves significantly, though it takes longer — often 12 to 24 months — and regular physical activity and a healthy diet after recovery will help that process considerably.

Now, for the stretch marks — I want to be honest with you, because I respect you too much to offer false reassurance. The purple color you see now is from active inflammation in the skin where the collagen has been torn by rapid stretching combined with cortisol-mediated skin fragility. The good news is that as your cortisol normalizes, that purple color will fade — often over several months — and the striae will gradually turn white or silver, as stretch marks typically appear in healthy skin.

However, the structural change to the skin — the actual mark itself — does not fully disappear, because the collagen fibers that were disrupted cannot fully regenerate to their original state. What you will likely be left with are faded, silvery marks that are far less visible than what you see today. Some patients find that over several years they become barely noticeable; others may wish to explore dermatological treatments such as laser therapy or microneedling after full remission, which can further improve their appearance.

I want you to know something important: the fact that these marks exist means your body went through something genuinely difficult — this was a real illness, not a lifestyle choice, and none of this was your fault. Your body did its best to cope. And now we are going to treat the cause and give your body the chance to heal.

The person you were before this illness started is still there. And with successful treatment, most of her is coming back."

Key Teaching Point: Two abnormal tests from different modalities are required to biochemically confirm Cushing's syndrome before any imaging is performed — imaging must never drive the diagnosis. BIPSS is the gold standard for distinguishing pituitary from ectopic ACTH when non-invasive testing is discordant. Nelson's syndrome is a critical long-term risk after bilateral adrenalectomy and mandates lifelong pituitary surveillance.

CASE-9

Clinical Case Evaluation: Hypertension with Proteinuria in Type 1 Diabetes

---

1. Diagnosis & Differential Diagnosis

Most Likely Diagnosis: Diabetic Nephropathy (Diabetic Kidney Disease — DKD)

This case presents with surgical precision the classic triad of established diabetic nephropathy: **hypertension**, **significant proteinuria** (foamy urine with 3+ dipstick protein), and **reduced renal function** (elevated creatinine at 1.4 mg/dL) in a young man with Type 1 Diabetes. The foamy appearance of urine is caused by the surface tension-lowering effect of albumin — a symptom patients often notice and report before any formal testing, making it a clinically important historical clue that should never be dismissed.

Pathophysiological Cascade of Diabetic Nephropathy: Chronic hyperglycemia initiates a devastating and self-amplifying sequence of renal injury. Advanced glycation end-products (AGEs) accumulate in the glomerular basement membrane, causing structural thickening. Intraglomerular hypertension develops from afferent arteriolar vasodilation and efferent arteriolar constriction mediated by angiotensin II, causing hyperfiltration that mechanically stresses podocytes. Podocyte injury disrupts the filtration barrier, allowing albumin to leak into the tubular lumen. TGF-β driven mesangial expansion and Kimmelstiel-Wilson nodule formation — the pathognomonic lesion of diabetic nephropathy — progressively obliterate glomerular surface area. Simultaneously, tubular inflammation and interstitial fibrosis driven by proteinuria-induced tubular toxicity accelerate the progression toward end-stage renal disease (ESRD).

The natural history of untreated Type 1 DKD progresses predictably: normalbuminuria → microalbuminuria (30–300 mg/day) → macroalbuminuria (>300 mg/day, as in this patient) → declining GFR → ESRD over 10–20 years. This patient has reached the macroalbuminuria stage with early GFR reduction — a critical inflection point where aggressive intervention can meaningfully alter trajectory.

Differential Diagnoses — Critical to Consider Before Attributing All Proteinuria to Diabetes:

- **IgA Nephropathy** — most common primary glomerulonephritis worldwide; can occur independently in a diabetic patient; hematuria is more prominent; renal biopsy differentiates; consider if atypical features present
- **Focal Segmental Glomerulosclerosis (FSGS)** — primary podocytopathy causing nephrotic-range proteinuria; may coexist with or mimic diabetic nephropathy; biopsy required for definitive diagnosis
- **Membranous Nephropathy** — autoimmune podocytopathy (anti-PLA2R antibody); causes heavy proteinuria; may occur in diabetic patients independently; check anti-PLA2R antibody
- **Lupus Nephritis** — young patients with proteinuria and hypertension; check ANA, anti-dsDNA, complement levels; particularly important to exclude in young women
- **Hypertensive Nephrosclerosis** — chronic hypertension causing glomerular ischemia and proteinuria; the chicken-and-egg relationship between hypertension and proteinuria in diabetes makes this diagnostically challenging
- **Non-diabetic Renal Disease in a Diabetic Patient** — up to 30% of diabetic patients with proteinuria have a non-diabetic etiology on biopsy; clinical features suggesting non-diabetic cause include: absence of diabetic retinopathy (retinopathy almost universally precedes nephropathy in Type 1 DM), rapid onset of proteinuria, significant hematuria, short diabetes duration (<5 years), or systemic features suggesting alternative diagnosis

Indications for Renal Biopsy in a Diabetic Patient: Atypical features as above; absence of retinopathy; abrupt onset nephrotic syndrome; active urinary sediment; unexpectedly rapid GFR decline; suspicion of superimposed glomerulonephritis — biopsy should not be withheld simply because the patient has diabetes.

---

2. Diagnostic Workup

Most Important Calculation: Estimated Glomerular Filtration Rate (eGFR)

eGFR is the cornerstone metric for staging chronic kidney disease severity, guiding treatment decisions, determining medication dosing safety, and prognosticating renal outcomes. It is calculated from serum creatinine using validated equations:

CKD-EPI 2021 Creatinine Equation (race-free, currently recommended): Incorporates serum creatinine, age, and sex — provides the most accurate GFR estimation across the full range of kidney function and is now the preferred equation per KDIGO 2022 guidelines, replacing the MDRD equation.

For this patient (28-year-old male, creatinine 1.4 mg/dL): Estimated eGFR ≈ **55–60 mL/min/1.73m²** — placing him in **CKD Stage G3a** (mild-to-moderately decreased kidney function, eGFR 45–59)

CKD Staging by GFR (KDIGO 2022):

- G1: ≥90 mL/min (normal or high — if albuminuria present)
- G2: 60–89 mL/min (mildly decreased)
- G3a: 45–59 mL/min (mild-to-moderately decreased)
- G3b: 30–44 mL/min (moderately-to-severely decreased)
- G4: 15–29 mL/min (severely decreased)
- G5: <15 mL/min (kidney failure/ESRD)

Albuminuria Staging (equally critical — dual staging system):

- A1: <30 mg/g (normal to mildly increased)
- A2: 30–300 mg/g (moderately increased — microalbuminuria)
- A3: >300 mg/g (severely increased — macroalbuminuria) ← **This patient**

This patient's CKD classification: G3a A3 — high-to-very-high risk category for CKD progression and cardiovascular events per KDIGO heat map; requires nephrology involvement and aggressive multifactorial intervention.

Complete Diagnostic Workup:

Quantification of Proteinuria:

- **Urine albumin-to-creatinine ratio (UACR)** on first-morning void — preferred over 24-hour urine collection (equivalent accuracy, far more practical); >300 mg/g confirms macroalbuminuria; values >3500 mg/g suggest nephrotic-range proteinuria
- **24-hour urine protein** — if UACR suggests nephrotic range or clinical concern for non-diabetic glomerulonephritis; protein >3.5g/24h = nephrotic syndrome

Renal and Metabolic Assessment:

- **Serum creatinine with eGFR calculation** (CKD-EPI 2021)
- **Cystatin C-based eGFR** — more accurate than creatinine-based in early CKD and in patients with abnormal muscle mass; confirmatory when creatinine-based eGFR is borderline
- **Serum electrolytes** — hyperkalemia risk with RAAS blockade; monitor closely
- **Serum bicarbonate** — metabolic acidosis in CKD accelerates disease progression; treat if <22 mEq/L
- **Urine microscopy** — red cell casts suggest active glomerulonephritis (non-diabetic etiology); oval fat bodies in nephrotic syndrome
- **Fasting glucose, HbA1c** — quantify glycemic control; target HbA1c <7% per ADA/EASD
- **Complete lipid panel** — diabetic dyslipidemia accelerates both cardiovascular and renal progression

Diabetic Complication Screening:

- **Dilated fundoscopy** — diabetic retinopathy assessment; presence of retinopathy strongly supports diabetic nephropathy etiology; its absence should prompt biopsy consideration
- **Peripheral neuropathy assessment** — monofilament testing, vibration sense
- **Ankle-Brachial Index (ABI)** — peripheral arterial disease screening

Immunological Screen (to exclude non-diabetic GN):

- ANA, anti-dsDNA, complement C3/C4 — lupus nephritis
- Anti-PLA2R antibody — membranous nephropathy
- ANCA (MPO, PR3) — vasculitis
- Serum protein electrophoresis (SPEP) — paraprotein-related nephropathy
- HIV, hepatitis B and C serology — secondary membranous or MPGN

Renal Ultrasound:

- Assess kidney size (enlarged in early DKD — hyperfiltration phase; small echogenic kidneys in advanced CKD), cortical thickness, echogenicity, hydronephrosis, and anatomical abnormalities; bilateral enlarged echogenic kidneys support diabetic nephropathy

---

3. Management Plan

First-Line and Specifically Indicated Antihypertensive Class: ACE Inhibitors (ACEi) or Angiotensin Receptor Blockers (ARBs)

Per ADA Standards of Care 2024, KDIGO Diabetes Management in CKD 2022, and ESH/ESC 2023 guidelines, **ACEi or ARB therapy is the mandatory first-line**

antihypertensive choice in any diabetic patient with albuminuria regardless of BP level — and is doubly indicated here given both hypertension and macroalbuminuria.

Mechanism of Renoprotection Beyond BP Lowering:

- Preferential dilation of the efferent arteriole → reduction of intraglomerular hypertension → decreased mechanical stress on podocytes → reduced proteinuria
- Reduction of TGF-β-mediated mesangial expansion and glomerulosclerosis
- Anti-fibrotic effects on tubular interstitium
- Proteinuria reduction of 30–50% independent of BP effect — this antiproteinuric effect is the primary renoprotective mechanism
- Landmark trials: **DCCT/EDIC** (glycemic control), **IRMA-2** (irbesartan preventing progression to macroalbuminuria), **IDNT and RENAAL** (irbesartan and losartan reducing ESRD risk in Type 2 DM with macroalbuminuria — extrapolated to Type 1)

Practical Prescribing:

- Start **lisinopril 5–10mg daily** or **ramipril 2.5–5mg daily** (ACEi) OR **losartan 50mg daily** or **irbesartan 150mg daily** (ARB)
- Titrate to maximum tolerated dose — higher doses provide greater antiproteinuric effect
- **Do NOT combine ACEi + ARB** — dual RAAS blockade increases hyperkalemia and AKI risk without additional renoprotective benefit (VA NEPHRON-D and ONTARGET trials); this combination is contraindicated
- Recheck serum creatinine and potassium at **1–2 weeks** after initiation — acceptable creatinine rise <30% from baseline; if >30%, hold and investigate for bilateral renal artery stenosis
- **BP target: <130/80 mmHg** per ADA 2024 and ESH/ESC 2023 in diabetic CKD with albuminuria; <120/80 mmHg if tolerated per ACC/AHA 2017

Transformative Add-On Therapies — Now Guideline-Recommended:

SGLT2 Inhibitors (Sodium-Glucose Cotransporter-2 Inhibitors):

- **Empagliflozin, dapagliflozin, or canagliflozin** — now first-line renoprotective agents alongside ACEi/ARB per KDIGO 2022 and ADA 2024 in patients with eGFR ≥20 mL/min and UACR >200 mg/g
- Mechanism: tubuloglomerular feedback restoration via macula densa sodium delivery, reducing intraglomerular hypertension; anti-inflammatory and anti-fibrotic effects; hemodynamic and metabolic benefits
- **CREDENCE trial** (canagliflozin) and **DAPA-CKD trial** (dapagliflozin) demonstrated 30–40% reduction in ESRD, doubling of creatinine, and renal death — among the most impactful nephrology trial results in a generation
- Also provides cardiovascular benefit, weight reduction, and modest BP lowering
- Safe to initiate down to eGFR ≥20 (though glucose-lowering effect diminishes below eGFR 45)

Finerenone (Non-steroidal MRA):

- Novel selective mineralocorticoid receptor antagonist; superior to spironolactone in renal fibrosis reduction with lower hyperkalemia risk
- **FIDELIO-DKD and FIGARO-DKD trials** — significant reduction in CKD progression and cardiovascular events in diabetic CKD
- Now recommended as add-on therapy in diabetic CKD with UACR >300 mg/g despite maximum ACEi/ARB
- Monitor potassium closely; avoid if eGFR <25 or potassium >5.0 mEq/L at initiation

GLP-1 Receptor Agonists:

- **Semaglutide** — FLOW trial (2024) demonstrated significant reduction in kidney failure, cardiovascular death, and GFR decline in diabetic CKD; now emerging as a fourth pillar of diabetic nephropathy management alongside ACEi/ARB, SGLT2i, and finerenone

Comprehensive Multifactorial Risk Factor Management:

- **Glycemic control** — HbA1c target <7% (ADA); each 1% HbA1c reduction decreases microvascular complication risk by ~25%; avoid hypoglycemia in CKD (reduced insulin clearance and gluconeogenesis)
- **High-intensity statin therapy** — atorvastatin 40–80mg; diabetic CKD confers very high cardiovascular risk; target LDL <55 mg/dL per ESC 2021
- **Dietary protein restriction** — 0.8g/kg/day of high biological value protein; reduces intraglomerular hyperfiltration and proteinuria-driven tubular toxicity; refer to renal dietitian
- **Dietary sodium restriction** — <2g/day; potentiates RAAS blockade efficacy
- **Smoking cessation** — smoking is an independent accelerator of DKD progression
- **Phosphate restriction** if eGFR declining below 45 mL/min**
- **Bicarbonate supplementation** if serum bicarbonate <22 mEq/L — slows CKD progression

---

4. Long-term Follow-up Protocol

Frequency Guided by CKD Stage and Albuminuria Category (KDIGO 2022): This patient (G3a A3) requires **monitoring every 3 months** at minimum.

Every Visit (Every 3 Months):

- Office BP measurement (bilateral) and home BP diary review — target <130/80 mmHg
- Serum creatinine, eGFR calculation — track trajectory; eGFR decline >5 mL/min/year or >10 mL/min over 5 years indicates rapid progression warranting urgent nephrology review
- Serum potassium — critical with ACEi/ARB and finerenone; target 3.5–5.0 mEq/L

- Serum bicarbonate
- HbA1c — every 3 months until stable, then every 6 months
- UACR — quantify antiproteinuric response to therapy; target >30% reduction from baseline
- Weight, BMI, fluid status assessment
- Review medication adherence, tolerability, and dose optimization

Every 6 Months:

- Complete metabolic panel including calcium, phosphorus, uric acid
- Lipid panel — ensure LDL target achieved on statin therapy
- CBC — anemia of CKD develops as eGFR declines; check hemoglobin; if Hb <10 g/dL, evaluate iron stores and consider erythropoiesis-stimulating agent
- Urine microscopy — monitor for hematuria suggesting non-diabetic process
- SGLT2 inhibitor-specific monitoring: genital mycotic infections, urinary tract infections, euglycemic DKA risk (particularly in Type 1 DM — requires careful monitoring; SGLT2i use in T1DM for renal protection requires specialist supervision)

Annually:

- **24-hour ambulatory BP monitoring** — detect masked hypertension and non-dipping pattern
- **Dilated fundoscopy** — diabetic retinopathy progression mirrors nephropathy; worsening retinopathy signals inadequate metabolic control
- **Peripheral neuropathy assessment** — monofilament, vibration, ankle reflexes
- **ABI and foot examination** — peripheral vascular disease and diabetic foot risk
- **Echocardiogram** — LV hypertrophy, diastolic dysfunction, and cardiomyopathy assessment; cardiovascular disease is the leading cause of death in diabetic CKD
- **Renal ultrasound** — every 1–2 years; monitor kidney size (shrinking kidneys = progressive fibrosis)
- **Bone mineral density (DEXA)** — CKD-mineral bone disorder assessment when eGFR <45; PTH, vitamin D, calcium, phosphorus panel annually below this threshold
- **Vaccination status** — influenza annually; pneumococcal, hepatitis B (impaired vaccine response in CKD — check anti-HBs titers); COVID-19 per current guidelines

Nephrology Referral Triggers (KDIGO 2022):

- eGFR <30 mL/min/1.73m^2 (CKD G4) — mandatory referral; prepare for renal replacement therapy planning
- eGFR decline >5 mL/min/year
- UACR >300 mg/g — already met; concurrent nephrology involvement recommended
- Resistant hypertension despite ≥3 agents

- Hyperkalemia refractory to dietary and medication management
- Atypical features suggesting non-diabetic CKD requiring biopsy
- **AV fistula creation discussion when eGFR <20** — if hemodialysis trajectory anticipated; early referral is critical as fistula maturation takes 3–6 months

---

5. Patient Education — *"Does protein in my urine mean my kidneys are failing?"*

"I can hear the worry in that question, and I want to address it directly and honestly — because you deserve a clear answer, not vague reassurance.

The short answer is: no, protein in your urine does not mean your kidneys are failing right now. But it does mean your kidneys are under significant stress, and it is a serious warning sign that we need to act on together — today, not someday.

Let me explain what is actually happening. Your kidneys contain millions of tiny filters called glomeruli. In a healthy kidney, these filters are incredibly precise — they keep large, important proteins like albumin inside your bloodstream where they belong. In your situation, years of high blood sugar have gradually damaged those filters, making them leaky. The foamy urine you noticed is actually albumin escaping into your urine — your body losing something it needs to keep.

Now, here is the critical and genuinely hopeful part of this conversation: we caught this at a stage where we can make a real difference. Your kidneys are working at reduced capacity right now, but they are far from failing. With the right treatment — and there are now more effective treatments for this than ever before in the history of medicine — we can significantly slow, and in some cases nearly stop, the progression of this kidney disease.

The medicines I am recommending — particularly the blood pressure medication called an ACE inhibitor — do something remarkable. They don't just lower your blood pressure; they specifically reduce the pressure inside those tiny kidney filters, allowing them to heal and leak less protein. We also have newer medications, originally developed for diabetes, that have been proven in large clinical trials to protect the kidneys powerfully.

But I also want to be transparent with you about what this means going forward. This is a long-term commitment that requires partnership between us. Your blood sugar control, your blood pressure, your diet, and your willingness to take these medications consistently will determine your kidney's future more than anything I can do in this office. The choices you make every day matter enormously.

Protein in your urine is not a death sentence for your kidneys. It is an alarm bell — and you heard it, and you came in, and now we are going to answer it together. That is exactly the right response."

Key Teaching Point: Diabetic nephropathy management has been revolutionized by four pillars: ACEi/ARB for intraglomerular pressure reduction, SGLT2 inhibitors for tubuloglomerular feedback restoration, finerenone for anti-fibrotic mineralocorticoid blockade, and GLP-1 receptor agonists for cardiorenal protection. The absence of diabetic retinopathy in a diabetic patient with proteinuria should always prompt consideration of renal biopsy to exclude non-diabetic glomerular disease. UACR on first morning void is the most practical and validated quantification tool for albuminuria monitoring.

CASE-10

Clinical Case Evaluation: Pulsatile Tinnitus, Carotid Bruit, and Hypertension in a Young Female Athlete

---

1. Diagnosis & Differential Diagnosis

Most Likely Diagnosis: Fibromuscular Dysplasia (FMD)

This case assembles the defining clinical portrait of fibromuscular dysplasia with exceptional clarity. A **young, lean woman** (the demographic epicenter of FMD — affecting women aged 20–60 in >90% of cases), **marathon runner** (high cardiovascular fitness making essential hypertension implausible), **pulsatile tinnitus** (pathognomonic of arterial wall abnormality transmitting turbulent flow to the inner ear via the carotid system), **neck pain** (carotid or vertebral artery involvement), **carotid bruit** (turbulent flow through dysplastic cervical artery), **epigastric bruit** (turbulent renal artery flow — the most common arterial territory affected in FMD), **significant hypertension** (renovascular origin from renal artery FMD), and **normal renal function** (preserved despite stenosis due to young age and compensatory mechanisms) — every single feature points toward FMD as the unifying diagnosis.

FMD is a non-inflammatory, non-atherosclerotic, idiopathic arteriopathy affecting medium-sized arteries, causing a spectrum of pathological changes in the arterial wall — from fibromuscular proliferation creating the classic "string of beads" appearance (alternating stenoses and aneurysms within the same vessel) to focal stenosis, dissection, and aneurysm formation. Unlike atherosclerosis, FMD involves the media and adventitia rather than the intima, occurs in the absence of cardiovascular risk factors, and preferentially affects the renal arteries (60–75% of cases) and cervicocephalic arteries (carotid and vertebral, 75–80% of cases) — frequently involving multiple arterial territories simultaneously, as in this patient.

Pathophysiological Mechanisms of Hypertension in Renal FMD: Multifocal stenoses in the renal artery reduce perfusion pressure to the juxtaglomerular apparatus, triggering RAAS activation via renin release — identical mechanistically to atherosclerotic renovascular hypertension but occurring in a completely different patient demographic and without the inflammatory and metabolic substrate of atherosclerosis. The hypertension is often severe, poorly responsive to standard antihypertensives, and potentially curable with revascularization.

Multi-Territory FMD in This Patient:

- **Renal arteries** (epigastric bruit, hypertension) — primary hemodynamic consequence
- **Carotid arteries** (carotid bruit, pulsatile tinnitus) — turbulent flow transmitted to tympanic vasculature
- **Vertebral arteries** (neck pain) — possible vertebral FMD causing mechanical symptoms and dissection risk
- **Intracranial arteries** — intracranial aneurysms occur in 7–10% of FMD patients; must be screened

Differential Diagnoses:

- **Atherosclerotic Renal Artery Stenosis** — overwhelmingly the most common cause of renovascular hypertension overall; however, completely incongruent with this

clinical picture: wrong age (typically >55), wrong sex predominance (more equal), wrong risk factor profile (no smoking, dyslipidemia, diabetes), wrong arterial morphology (focal ostial or proximal plaque vs. mid-vessel string of beads); carotid and epigastric bruits would be unusual bilaterally without atherosclerotic risk factors

- **Takayasu Arteritis** — large vessel granulomatous vasculitis affecting the aorta and its primary branches; young Asian women; however, characterized by inflammatory features (elevated ESR, CRP, constitutional symptoms — fever, malaise, weight loss), wall thickening and enhancement on vessel wall MRI, absent/diminished pulses rather than bruits, and involvement of large proximal vessels rather than mid-vessel segments; the absence of systemic inflammation distinguishes FMD clearly
- **Neurofibromatosis Type 1 (NF1)** — causes renovascular hypertension and arterial dysplasia via neurofibromin loss; look for café-au-lait spots, axillary freckling, Lisch nodules, neurofibromas; mid-aortic syndrome can occur; genetic testing for NF1 mutation if clinical features present
- **Spontaneous Carotid Artery Dissection** — occurs in young patients, athletes, and those with connective tissue disorders; neck pain and pulsatile tinnitus overlap with FMD; critically, FMD itself predisposes to dissection — the two diagnoses are not mutually exclusive and frequently coexist; FMD is found in 15–20% of patients presenting with spontaneous cervical artery dissection
- **Connective Tissue Disorders (Ehlers-Danlos Syndrome Type IV, Marfan Syndrome, Loeys-Dietz Syndrome)** — share arterial fragility, aneurysm formation, and dissection risk with FMD; vascular EDS (COL3A1 mutation) in particular causes arterial dissection and rupture in young patients; skin hyperextensibility, joint hypermobility, easy bruising; genetic testing indicated if clinical features present; FMD may represent a forme fruste of a broader arteriopathy spectrum
- **Segmental Arterial Mediolysis (SAM)** — rare non-inflammatory, non-atherosclerotic arteriopathy of visceral arteries; causes spontaneous dissection and hemorrhage; overlapping features with FMD and may represent the same disease spectrum
- **Polyarteritis Nodosa** — medium vessel vasculitis; causes renal artery microaneurysms and hypertension; associated with hepatitis B; inflammatory markers elevated; renal biopsy or angiography differentiates
- **Essential Hypertension** — categorically implausible in a 28-year-old lean female marathon runner with bilateral arterial bruits, pulsatile tinnitus, and neck pain; must never be accepted as a diagnosis without complete secondary hypertension exclusion in this demographic

---

2. Why Renal Artery Duplex Ultrasound is Sometimes a "Trap" in FMD Diagnosis

This is one of the most clinically important and frequently underappreciated diagnostic pitfalls in renovascular medicine, and understanding it is essential to avoiding a false-negative diagnosis with serious consequences.

The Anatomical Trap:

Renal artery duplex ultrasound is an excellent first-line screening tool for **atherosclerotic** renal artery stenosis, which characteristically affects the **ostium and proximal renal artery**

— a location that is relatively accessible to ultrasound beam penetration and Doppler interrogation.

FMD, however, almost exclusively affects the **mid and distal segments of the renal artery**, and frequently extends into the **segmental and interlobar branches** — vascular territories that are anatomically deep, obscured by bowel gas and retroperitoneal fat, technically inaccessible to reliable Doppler interrogation, and beyond the spatial resolution of standard duplex ultrasound in most clinical settings.

Specific Reasons Duplex Ultrasound Fails in FMD:

Anatomical inaccessibility — the mid and distal renal artery lies deep in the retroperitoneum, beyond the reliable Doppler window in most patients, particularly those with any body habitus or bowel gas interference; even in optimal conditions, the distal third of the renal artery is visualized in fewer than 50% of examinations

Morphological mismatch — duplex ultrasound detects hemodynamic stenosis by measuring peak systolic velocity (PSV >180–200 cm/s) and velocity ratios; the "string of beads" morphology of FMD creates multiple alternating stenoses and post-stenotic dilatations that may not produce a single dominant high-velocity jet detectable by standard Doppler criteria; the integrated hemodynamic effect may be underestimated

Operator dependency — renal duplex is notoriously operator-dependent; FMD's distal location requires exceptional technical skill and dedicated scanning protocols that many centers, including experienced vascular laboratories, do not routinely apply to the distal renal vasculature

False negative rate — studies suggest duplex ultrasound misses FMD in up to **50–60% of confirmed cases** when compared to the gold standard; a normal or equivocal duplex study in a young woman with clinical features of FMD must absolutely not be interpreted as exclusionary

The Clinical Consequence of This Trap: A clinician who orders renal duplex ultrasound, receives a "normal" or "technically limited" report, and concludes that renovascular hypertension has been excluded in this patient will miss the diagnosis, fail to refer for definitive imaging, and condemn the patient to years of uncontrolled hypertension with progressive end-organ damage and arterial complication risk — when she may have been entirely curable.

The Correct Diagnostic Approach in Suspected FMD:

CTA or MRA of the renal and cervicocephalic arteries — cross-sectional vascular imaging is the appropriate first-line diagnostic tool in suspected FMD, not duplex ultrasound:

- **CT Angiography (CTA) with multi-detector reconstruction** — provides excellent spatial resolution of the entire renal artery from ostium to segmental branches; clearly demonstrates the "string of beads" morphology; simultaneously images the aorta, mesenteric vessels, and iliac arteries for multi-territory disease; rapid acquisition; limitation is radiation exposure and contrast nephrotoxicity (less relevant here given normal renal function and young age)
- **MR Angiography (MRA)** — preferred in young patients to minimize radiation; gadolinium-enhanced MRA provides excellent renal and cervicocephalic arterial imaging without ionizing radiation; slightly lower spatial resolution than CTA for small distal lesions; preferred for long-term surveillance to minimize cumulative radiation

- **Catheter-Based Digital Subtraction Angiography (DSA)** — gold standard for definitive diagnosis and simultaneous therapeutic intervention (PTRA); provides superior spatial resolution for segmental and branch vessel disease; allows pressure gradient measurement across stenoses; reserved for cases where CTA/MRA is inconclusive or when revascularization is planned

Cervicocephalic Imaging — Mandatory in All FMD Patients: Given the carotid bruit and tinnitus in this patient, CTA or MRA of the **neck and brain** must be performed to:

- Characterize carotid and vertebral FMD
- Screen for **intracranial aneurysms** (present in 7–10% of FMD patients) — a potentially life-threatening finding requiring separate management consideration
- Detect arterial dissection (carotid or vertebral) which may be present simultaneously

---

3. Management Plan

Preferred Treatment: Percutaneous Transluminal Renal Angioplasty (PTRA) Without Stenting

This patient is an ideal candidate for revascularization. Young age, preserved renal function, likely short duration of hypertension, multi-territory FMD confirming systemic arteriopathy, and a potentially curable cause of severe hypertension make intervention the treatment of first choice per the **2019 American Heart Association Scientific Statement on FMD** and **European FMD Consensus 2021**.

Why PTRA Without Stenting (Balloon Angioplasty Alone):

In FMD-related renal artery stenosis, balloon angioplasty alone achieves **technical success in >90% of cases** with excellent long-term patency — fundamentally different from atherosclerotic RAS where stenting is required to prevent elastic recoil of calcified plaque. The "string of beads" lesions of medial FMD respond beautifully to balloon dilation, which disrupts the fibromuscular webs and restores normal luminal diameter without the need for metallic stent implantation. Avoiding stents preserves future re-intervention options, eliminates stent fracture risk (particularly important in a young active patient), and avoids the neointimal hyperplasia that drives in-stent restenosis.

Expected Outcomes Post-PTRA in Young FMD Patients:

- **Hypertension cure** (normalization without medications): 35–50% of patients — highest rates in young women with short hypertension duration
- **Hypertension improvement** (significant reduction in BP or medication requirements): additional 40–45%
- **Overall benefit rate >85%** in appropriately selected young patients
- Technical failure or restenosis requiring repeat PTRA: ~10–15%; easily re-treated with repeat balloon angioplasty

Perioperative and Procedural Considerations:

- Performed via femoral or radial arterial access under conscious sedation

- Intraoperative **pressure wire measurement** across each stenosis — fractional flow reserve (FFR) or transstenotic gradient assessment confirms hemodynamic significance before dilation
- **Intravascular ultrasound (IVUS)** or **optical coherence tomography (OCT)** — characterize lesion morphology and guide balloon sizing
- **Intraoperative heparin anticoagulation** — prevent thromboembolic complications
- **Antihypertensive medications continued** until procedural day; anticipate BP normalization requiring rapid medication weaning post-procedure to avoid hypotension

Cervicocephalic FMD Management:

- **Asymptomatic carotid FMD** — antiplatelet therapy (aspirin 75–100mg daily) and observation with serial imaging; revascularization not routinely recommended for asymptomatic carotid FMD
- **Symptomatic carotid FMD** (TIA, stroke, dissection) — carotid angioplasty (without stenting where anatomy permits) at an experienced center
- **Intracranial aneurysms** — neurovascular multidisciplinary assessment; management individualized based on size, location, morphology, and patient factors; aneurysms ≥7mm or with high-risk features typically treated (endovascular coiling preferred over surgical clipping in most centers)
- **Vertebral artery FMD** — antiplatelet therapy; revascularization if symptomatic posterior circulation ischemia

Medical Therapy (Adjunct — Not Definitive):

- **ACE inhibitors or ARBs** — effective BP control pre-procedure and in patients unsuitable for intervention; reduces intraglomerular hypertension; **use with caution in bilateral renal FMD** — monitor creatinine and potassium closely
- **Antiplatelet therapy (aspirin 75–100mg daily)** — recommended lifelong in all FMD patients with cervicocephalic involvement; reduces dissection and thromboembolic risk
- **Calcium channel blockers (amlodipine)** — safe and effective adjunct antihypertensive without RAAS concerns
- **Avoid beta-blockers as monotherapy** — may theoretically worsen renal perfusion in renovascular hypertension; also limits exercise performance in a marathon runner

---

4. Long-term Follow-up Protocol

FMD is a systemic arteriopathy requiring lifelong structured surveillance — successful renal PTRA does not eliminate the underlying disease process.

Immediate Post-PTRA (First 48 Hours):

- Frequent BP monitoring — anticipate dramatic BP reduction; rapidly wean antihypertensives to prevent hypotension

- Serum creatinine and electrolytes at 24–48 hours — detect contrast nephropathy (unlikely with normal baseline renal function) or hemodynamic renal hypoperfusion
- Vascular access site assessment — hematoma, pseudoaneurysm, retroperitoneal bleed

At 1 Month:

- Office BP and HBPM diary — quantify antihypertensive response; adjust or discontinue medications as BP allows
- Serum creatinine, electrolytes
- Clinical assessment of carotid and tinnitus symptoms — improvement in pulsatile tinnitus suggests reduced carotid turbulence

At 3 and 6 Months:

- **ABPM** — objective 24-hour BP assessment; confirm cure or improvement
- **MRA of renal arteries** — assess PTRA result, detect restenosis (occurs in ~10–15%); MRA preferred over CTA to minimize cumulative radiation in young patient
- **MRA of cervicocephalic arteries** — monitor carotid and vertebral FMD stability; screen for new dissection
- Repeat clinical neurological and vascular assessment
- Antiplatelet therapy adherence review

Annually — Lifelong:

- **Office BP bilaterally and ABPM** — hypertension recurrence indicates restenosis or progression in contralateral or new arterial territory
- **MRA of renal, mesenteric, cervicocephalic, and intracranial arteries** — comprehensive multi-territory surveillance; FMD can progress or manifest in new territories over time; **intracranial aneurysm surveillance** mandatory (re-image every 1–3 years depending on aneurysm characteristics)
- **Renal function** — serum creatinine, eGFR, UACR annually
- **Pregnancy counseling** — FMD and pregnancy intersect critically; pregnancy-related hemodynamic and hormonal changes can accelerate arterial dissection and aneurysm growth; all pregnancies in FMD patients classified as **high-risk**; pre-conception multidisciplinary planning with maternal-fetal medicine, cardiology, and vascular surgery mandatory; antihypertensive management requires modification (ACEi/ARBs contraindicated in pregnancy — switch to labetalol, nifedipine, or methyldopa)
- **FMD Patient Registry enrollment** — strongly encouraged; the United States Registry for Fibromuscular Dysplasia and European FMD Registry provide longitudinal outcome data guiding evolving management recommendations; patients benefit from registry participation through access to specialist expertise and clinical trial opportunities

- **Genetic counseling** — FMD has familial clustering; first-degree relatives (particularly female) should be offered screening with MRA if index patient has confirmed multi-territory disease; evaluate for associated connective tissue disorders

---

5. Patient Education — *"I heard I shouldn't do high-impact sports or neck adjustments?"*

"That is an incredibly important question, and I am really glad you brought it up — because the answer requires some nuance, and getting it right matters enormously for both your safety and your quality of life as an athlete.

Let me start with the neck adjustments, because this one is more straightforward. Chiropractic cervical manipulation — the kind where your neck is rapidly rotated or thrust — carries a small but real and well-documented risk of causing a carotid or vertebral artery dissection in any person. In someone with FMD like you, where the arterial walls in your neck are inherently more vulnerable and structurally different from a normal artery, that risk is meaningfully higher. A dissection of the carotid or vertebral artery can cause a stroke. This is not a theoretical concern — it has been reported in FMD patients following cervical manipulation. My recommendation is clear and firm: please avoid high-velocity cervical manipulation from any practitioner. If you have neck pain — and I know you mentioned that — we can absolutely address it through physical therapy focusing on gentle mobilization, strengthening, and postural techniques that are safe for your arterial anatomy.

Now, marathon running — this is where I want to be thoughtful rather than reflexively restrictive, because your identity as an athlete matters, and blanket exercise prohibition would be both incorrect and harmful to your wellbeing.

The current evidence and expert consensus on FMD does not support prohibiting aerobic exercise like running. In fact, regular moderate-to-vigorous aerobic exercise is beneficial for blood pressure control, cardiovascular health, and overall wellbeing — and we want to preserve that for you. There is no credible evidence that running marathons causes FMD to progress or increases dissection risk in a meaningful way.

However — and this is important — there are some specific considerations I want you to keep in mind. We need your blood pressure to be well-controlled before you return to competitive marathon training, because exercising hard with significantly elevated blood pressure does place additional hemodynamic stress on already-vulnerable arterial walls. Once we have treated the narrowing in your kidney artery and your blood pressure normalizes, returning to full marathon training is a very reasonable goal.

The activities I would ask you to be more cautious about are those involving extreme isometric straining — very heavy resistance training with maximum breath-holding and valsalva maneuver — because these create acute spikes in arterial wall tension. Moderate strength training is fine; powerlifting competition-style maximum effort lifting is the type of activity to moderate.

I also want you to be aware of the warning signs that should prompt you to stop exercise immediately and seek urgent evaluation: sudden severe headache unlike any you have had before, neck pain that begins suddenly during or after exercise, visual disturbances, facial drooping, arm or leg weakness, or difficulty speaking. These could indicate an arterial dissection and require emergency assessment.

Bottom line: you can run. You can live fully and actively. We just need to do it smartly, with your blood pressure controlled, your arteries monitored, and your awareness sharp. Being an athlete does not conflict with having FMD — it just means we need to be thoughtful partners in managing it together."

Key Teaching Point: Renal duplex ultrasound is an unreliable screening tool for FMD because the disease affects the mid and distal renal artery — territories beyond reliable Doppler interrogation in most laboratories. CTA or MRA is the appropriate first-line imaging in any young woman with clinical features of FMD. A normal duplex study never excludes FMD. PTRA without stenting is the preferred treatment, achieving cure or meaningful improvement in >85% of appropriately selected young patients. All FMD patients require lifelong multi-territory arterial surveillance and intracranial aneurysm screening.
