# Supplementary material for: Diagnostic Performance and Workup Efficiency of Large Language Models in Secondary Hypertension: A Blinded Comparative Study
Source: Diagnostics (Basel). 2026 Jul 10;16(14):2165. doi: 10.3390/diagnostics16142165 (PMC13409298; doi:10.3390/diagnostics16142165)
Supplement: Supplementary file 1 [file diagnostics-16-02165-s001.zip › Supplementary file S2/6. LLM_Water.pdf]

CASE-1

1) Diagnosis & Differential Diagnosis

Most Likely Diagnosis:
Pheochromocytoma — a catecholamine-secreting tumor of the adrenal medulla.

Pathophysiological justification:

- Episodic headache, diaphoresis, and palpitations (classic triad)
- Paroxysmal severe hypertension (210/115 mmHg)
- Tachycardia (125 bpm)
- Precipitation by exercise (catecholamine surge trigger)
- Orthostatic hypotension (chronic vasoconstriction → volume contraction)

These findings strongly align with **catecholamine excess** due to chromaffin cell tumor.

Differential Diagnoses:

- **Paraganglioma** – extra-adrenal catecholamine-secreting tumor (same physiology).
- **Panic disorder** – episodic symptoms but typically normotensive or mildly elevated BP; no severe paroxysmal hypertension.
- **Thyrotoxicosis** – persistent tachycardia rather than episodic hypertensive crises.
- **Substance-induced sympathetic crisis** (cocaine, amphetamines) – history usually reveals exposure.
- **Renovascular hypertension** – sustained rather than episodic hypertension; abdominal bruit often present.

---

2) Diagnostic Workup

Most Sensitive Initial Screening Test:

- **Plasma free metanephrines** (supine sampling after 20–30 minutes rest).

This is the most sensitive test per Endocrine Society guidelines and preferred in high-suspicion cases.

Alternative (if low pretest probability):

- 24-hour urinary fractionated metanephrines.

Gold Standard Confirmatory Test:
There is no separate "gold standard" biochemical test beyond clearly elevated plasma or urinary metanephrines. If borderline elevation occurs, proceed with:

- **Clonidine suppression test** (if needed to distinguish false positives).

Imaging (after biochemical confirmation):

- **CT abdomen with adrenal protocol** (first-line imaging).
- MRI if CT contraindicated (e.g., pregnancy).

- Functional imaging (e.g., MIBG, PET) only if metastatic disease or hereditary suspicion.

Cost-effective approach:
Biochemical confirmation → targeted CT → no unnecessary advanced imaging unless indicated.

---

3) Management Plan

Acute Hypertensive Crisis (if unstable):

- IV nicardipine or phentolamine.
- Avoid beta-blocker monotherapy.

Preoperative Preparation (critical):

First medication to start:

- **Alpha-adrenergic blockade (phenoxybenzamine or selective alpha-1 blocker such as doxazosin).**

Rationale:
Beta-blockade before alpha-blockade can cause unopposed alpha stimulation → catastrophic hypertensive crisis.

Protocol:

- Alpha-blockade for 7–14 days.
- Liberal salt intake and fluid repletion to correct volume contraction.
- Add beta-blocker only after adequate alpha blockade if tachycardia persists.

Definitive Treatment:

- **Laparoscopic adrenalectomy.**

---

4) Long-Term Follow-up

Pheochromocytoma carries recurrence risk (especially in young patients).

Postoperative protocol:

- Measure plasma free metanephrines 2–6 weeks after surgery to confirm biochemical cure.
- If normalized → annual biochemical testing (lifelong recommended by Endocrine Society).

Imaging:

- Only if biochemical recurrence or hereditary mutation.

Genetic testing:
Recommended for all patients diagnosed under age 45 or with paraganglioma, bilateral disease, or family history.

Duration:

- **Lifelong annual surveillance** (biochemical testing).

---

5) Patient Education (Answer to: "Will I be off blood pressure meds forever after surgery?")

"Most patients experience significant improvement after the tumor is removed, and many no longer need blood pressure medications. However, this depends on how long the hypertension has been present and whether any permanent vascular changes have occurred.

If your high blood pressure is entirely caused by the tumor, there is a good chance it will resolve. That said, some patients may still require medication, although usually at lower doses.

We will monitor your blood pressure closely after surgery and adjust treatment based on your individual response."

CASE-2

1) Diagnosis & Differential Diagnosis

Most Likely Diagnosis:
Primary Aldosteronism (PA) — most likely due to **aldosterone-producing adenoma (APA)** or **bilateral adrenal hyperplasia (BAH)**.

Pathophysiological justification:

- Resistant hypertension (on ACE inhibitor + CCB + thiazide-like diuretic)
- Hypokalemia (K^+ 3.1 mEq/L) without loop diuretic
- Elevated plasma aldosterone concentration (PAC)
- Suppressed plasma renin activity (PRA)
- High aldosterone-to-renin ratio (ARR)

These findings are classic for autonomous aldosterone production.

Differential Diagnoses:

- **Secondary hyperaldosteronism** (e.g., renal artery stenosis): renin would be elevated.
- **Liddle syndrome:** low renin and low aldosterone.
- **Cushing syndrome:** may cause resistant HTN and hypokalemia, but aldosterone not elevated.
- **Apparent mineralocorticoid excess:** low renin, low aldosterone.

---

2) Diagnostic Workup

Initial Screening (already done):

- Elevated ARR (PAC high, PRA suppressed).

Confirmatory Test (to confirm PA):
One of the following (per Endocrine Society Guidelines):

- **Saline infusion test** (most commonly used)
- Oral sodium loading test
- Fludrocortisone suppression test
- Captopril challenge

In clear-cut cases (spontaneous hypokalemia + suppressed renin + PAC >20 ng/dL), confirmatory testing may be bypassed.

Gold Standard to Differentiate Unilateral vs Bilateral Disease:
Adrenal Vein Sampling (AVS).

Rationale:

- CT imaging alone cannot reliably distinguish unilateral adenoma from bilateral hyperplasia.
- AVS determines lateralization before surgery.
- Mandatory in most patients >35 years (unless very young with classic unilateral lesion).

---

3) Management Plan

If Unilateral Aldosterone-Producing Adenoma:

- Laparoscopic adrenalectomy.

If Bilateral Adrenal Hyperplasia (BAH):

First-line Medical Therapy:

- **Mineralocorticoid receptor antagonist (MRA)**

Preferred options:

- **Spironolactone** (first-line, cost-effective)
- Eplerenone (if spironolactone not tolerated)

Additional measures:

- Sodium restriction
- Optimize other antihypertensives as needed

Goal:

- Normalize potassium
- Raise renin into detectable range (marker of adequate blockade)
- Control BP

---

4) Long-Term Follow-up

If Surgical Treatment:

- Check potassium and BP within 1–2 weeks post-op
- Repeat ARR at 6–12 weeks
- Annual BP monitoring lifelong

If Medical Therapy (BAH):

- Monitor potassium and creatinine 1 week after starting or adjusting MRA
- Then every 4–6 weeks until stable
- Once stable: every 3–6 months
- Annual cardiovascular risk assessment

Treatment targets:

- BP <130/80 mmHg (per ESC/ESH guidance)
- Potassium normalized
- Renin no longer suppressed

Lifelong monitoring is required.

---

5) Patient Education (Gynecomastia Question)

"If gynecomastia develops, it is most commonly related to spironolactone. This medication can interact with androgen receptors and cause breast enlargement in some men.

In that case, we would switch you to **eplerenone**, which works similarly but has much less effect on sex hormone receptors.

If needed, we can also adjust the dose. The goal is to control your blood pressure and potassium while minimizing side effects. This issue is manageable and does not mean treatment has failed."

CASE-3

1) Diagnosis & Differential Diagnosis

Most Likely Diagnosis:
Atherosclerotic Renal Artery Stenosis (RAS) causing renovascular hypertension.

Pathophysiological justification:

- Age >60 with heavy smoking history and peripheral vascular disease
- Severe hypertension (170/105 mmHg)
- Rise in creatinine >30% after initiation of ACE inhibitor (1.1 → 1.8 mg/dL)
- Abdominal systolic–diastolic bruit

ACE inhibitors reduce efferent arteriolar tone. In significant RAS (especially bilateral or solitary functioning kidney), glomerular filtration becomes dependent on angiotensin II–mediated efferent constriction. Blocking RAAS causes abrupt GFR decline.

Differential Diagnoses:

- Bilateral renal artery stenosis
- Solitary kidney with stenosis
- Acute interstitial nephritis (no bruit, different timing pattern)
- Volume depletion–related AKI
- Fibromuscular dysplasia (less likely at this age; more common in younger women)

---

2) Diagnostic Workup

Initial Non-Invasive Screening Tool of Choice:
Renal artery duplex Doppler ultrasonography.

Rationale:

- Non-invasive
- No contrast exposure
- Cost-effective
- Provides flow velocity and resistive indices

If Doppler is inconclusive:

- CT angiography (if renal function allows)
- MR angiography (if contrast contraindicated)

Gold standard (rarely required initially):

- Digital subtraction angiography (reserved for intervention planning)

---

3) Management Plan

Initial Medical Therapy (first-line for most patients):

- Blood pressure control (ACEi/ARB can be continued cautiously if unilateral and creatinine stabilizes)
- High-intensity statin
- Antiplatelet therapy
- Smoking cessation
- Risk factor optimization

Definitive Management for Refractory Cases:
Percutaneous transluminal renal angioplasty with stenting

Indications (per ESC/ESH and KDIGO guidance):

- Recurrent flash pulmonary edema
- Rapidly declining kidney function
- Refractory hypertension despite optimal medical therapy
- Bilateral high-grade stenosis with progressive renal impairment

Routine stenting is NOT recommended in stable cases due to lack of outcome benefit in major trials (e.g., CORAL).

---

4) Long-Term Follow-up

If Managed Medically:

- Serum creatinine and potassium:
  - 1–2 weeks after medication adjustments
  - Then every 3–6 months
- BP monitoring every 1–3 months until controlled
- Annual renal ultrasound if progressive disease suspected

If Revascularized:

- Creatinine at 1–2 weeks post-procedure
- Duplex ultrasound at 6 months
- Then annually

Lifelong cardiovascular risk surveillance is essential.

---

5) Patient Education

"If the kidney artery is narrowed, your kidney relies on a hormone called angiotensin II to keep pressure inside the filtering unit high enough to function.

ACE inhibitors block that hormone. In a normal kidney, that's protective. But if there's a severe narrowing in the kidney artery, the kidney becomes dependent on that mechanism to maintain filtration. When we block it, filtration pressure drops and creatinine rises.

This doesn't mean the medication is harmful in general — it revealed that there may be a narrowing in the kidney artery. We will adjust treatment carefully and evaluate the artery further."

CASE-4

1) Diagnosis & Differential Diagnosis

Most Likely Diagnosis:
Primary Hyperparathyroidism (PHPT) — most likely due to a parathyroid adenoma.

Pathophysiological justification:

- Hypercalcemia (Ca 11.2 mg/dL)
- Suppressed serum phosphorus (PTH-mediated phosphaturia)
- Recurrent calcium oxalate nephrolithiasis
- Hypertension (common association)
- Neurocognitive symptoms (“brain fog”), constipation

These findings are classic for PTH-mediated hypercalcemia.

Differential Diagnoses:

- **Malignancy-associated hypercalcemia** (PTH suppressed, PTHrP elevated)
- **Familial hypocalciuric hypercalcemia (FHH)** (mild hypercalcemia, low urinary calcium, normal/mildly elevated PTH)
- **Vitamin D intoxication** (high Ca, high phosphate)
- **Sarcoidosis** (elevated 1,25-OH vitamin D, suppressed PTH)

---

2) Diagnostic Workup

Lab Combination that Confirms Diagnosis:

- Elevated serum calcium
- Elevated or inappropriately normal intact PTH

(High calcium with non-suppressed PTH confirms PHPT.)

Additional required tests:

- 25-OH vitamin D
- 24-hour urinary calcium (to exclude FHH)
- Renal function
- DEXA scan (lumbar spine, total hip, distal radius)

Imaging (only after biochemical confirmation):

- Neck ultrasound
- Sestamibi scan (for localization, not diagnosis)

---

3) Management Plan

Only Curative Treatment:
Parathyroidectomy.

Indications per Endocrine Society guidelines include:

- Symptomatic disease (stones, neurocognitive symptoms)
- Serum calcium >1 mg/dL above upper limit

- Nephrolithiasis
- Osteoporosis
- Age <50
- Reduced GFR

Medical therapy (if not surgical candidate):

- Cinacalcet (controls calcium, not curative)
- Bisphosphonates for bone protection

But surgery is definitive.

---

4) Long-Term Follow-up

After Surgery:

- Serum calcium at 1–2 weeks
- Calcium and PTH at 6 months
- Annual serum calcium lifelong

Bone monitoring:

- DEXA at baseline and 1 year post-op
- Then every 1–2 years

Renal monitoring:

- Periodic creatinine
- Imaging if stone recurrence suspected

If managed non-operatively:

- Calcium every 6 months
- DEXA every 1–2 years
- Annual renal assessment

---

5) Patient Education

“Many patients report improvement in concentration, energy, and mood after successful surgery. The high calcium level can affect brain function and cause symptoms like fogginess, low mood, or fatigue.

In most cases, these symptoms improve gradually over weeks to months after calcium normalizes. However, if symptoms have been present for a long time, improvement may not be immediate.

We will monitor your calcium closely after surgery and follow your symptoms over time. There is a good chance you will feel clearer mentally once your calcium levels return to normal.”

CASE-5

1) Diagnosis & Differential Diagnosis

Most Likely Diagnosis:
Primary (Essential) Hypertension – Early Onset.

Rationale:

- Persistent BP 145/92 mmHg (Stage 1–2 range depending on guideline)
- Normal BMI
- No secondary cause identified after appropriate workup
- Normal pulses and exam

In young adults, primary hypertension is still the most common cause once secondary etiologies are excluded.

Differential Diagnoses:

- **White Coat Hypertension (WCH)**
- **Masked Hypertension**
- Early **renovascular disease** (unlikely with negative workup)
- **Coarctation of the aorta** (normal femoral pulses argue against)
- **Primary aldosteronism** (unlikely without hypokalemia or suppressed renin)

---

2) Diagnostic Workup

To rule out White Coat Hypertension before lifelong therapy:

24-hour Ambulatory Blood Pressure Monitoring (ABPM) — preferred method.

Alternative:

- Structured Home Blood Pressure Monitoring (HBPM) over 7 days (twice daily readings).

ABPM provides:

- Daytime average
- Nighttime dipping pattern
- Detection of masked or sustained hypertension

Per ESC/ESH and ACC/AHA guidelines, out-of-office BP confirmation is mandatory before labeling a young patient with lifelong hypertension.

---

3) Management Plan

If ABPM confirms **Stage 1 Hypertension without target organ damage and low CV risk**:

First-line intervention:
Lifestyle modification.

Key measures:

- Sodium restriction (<5 g salt/day)
- Optimize potassium intake through diet
- Limit alcohol
- Ensure adequate sleep
- Avoid performance-enhancing stimulants
- Stress management

Trial duration:

- 3–6 months before pharmacologic therapy if BP <160/100 and no organ damage.

If BP remains elevated after lifestyle optimization → consider pharmacologic therapy.

---

4) Long-Term Follow-up

If White Coat Hypertension:

- Annual ABPM or HBPM
- Office BP every 6–12 months

If Confirmed Essential Hypertension (managed non-pharmacologically):

- Reassess every 3–6 months
- Annual labs (creatinine, electrolytes, fasting glucose, lipids)
- ECG every 2–3 years

If medication initiated:

- Follow-up 4–6 weeks after initiation
- Then every 3–6 months once controlled

Target BP (per ESC):

- <130/80 mmHg if tolerated

Lifelong monitoring is required.

---

5) Patient Education

"It's a common misconception that only people who are overweight or inactive develop high blood pressure. Genetics, vascular tone regulation, salt sensitivity, and even sympathetic nervous system activity can influence blood pressure — even in athletes.

Sometimes, intense training or stress can temporarily elevate readings. That's why we will confirm your blood pressure with 24-hour monitoring before labeling this as true hypertension.

If it is confirmed, it does not mean you've done something wrong. Many healthy individuals develop essential hypertension due to factors beyond lifestyle alone. Our goal is early prevention to protect your heart and blood vessels long-term."

CASE-6

1) Diagnosis & Differential Diagnosis

Most Likely Diagnosis:
Obstructive Sleep Apnea (OSA)–related hypertension.

Pathophysiological justification:

- BMI 34 (obesity is a major risk factor)
- Loud snoring and witnessed apneas
- Severe daytime sleepiness
- Morning headaches
- Early-morning BP surge

OSA leads to recurrent nocturnal hypoxia → sympathetic activation → RAAS activation → endothelial dysfunction → sustained hypertension, particularly non-dipping or morning-predominant hypertension.

Differential Diagnoses:

- Primary (essential) hypertension
- Secondary causes (primary aldosteronism, renal disease)
- Central sleep apnea (less likely without heart failure or neurologic disease)
- Cushing syndrome (no suggestive features provided)

---

2) Diagnostic Workup

Gold Standard Test:
Overnight in-laboratory polysomnography (PSG).

Measures:

- Apnea-Hypopnea Index (AHI)
- Oxygen desaturation
- Sleep architecture

Diagnostic criteria:
AHI ≥5 with symptoms or ≥15 regardless of symptoms.

Home sleep apnea testing may be acceptable in high-probability uncomplicated cases but PSG remains the gold standard.

---

3) Management Plan

First-line Treatment:
Continuous Positive Airway Pressure (CPAP).

Benefits:

- Reduces sympathetic activation
- Improves nocturnal oxygenation
- Lowers BP (average 5–10 mmHg reduction; greater effect in resistant HTN)
- Improves daytime alertness

Additional measures:

- Weight reduction (target ≥10% body weight loss)
- Alcohol avoidance at night
- Positional therapy (if positional OSA)

Antihypertensive therapy should be optimized concurrently (often ACEi/ARB + CCB ± diuretic).

---

4) Long-Term Follow-up

Initial follow-up:

- 1–3 months after CPAP initiation
- Assess adherence (≥4 hours/night on ≥70% nights)
- Reassess BP

Ongoing monitoring:

- BP every 3–6 months
- Annual metabolic evaluation (glucose, lipids)
- Weight monitoring
- CPAP compliance data review periodically

If weight loss achieved, repeat sleep study may be considered.

OSA management is lifelong unless substantial weight loss resolves disease.

---

5) Patient Education

“Yes, wearing the CPAP mask every night is important. The breathing pauses happen repeatedly during sleep, and each episode stresses your heart and blood vessels.

When untreated, sleep apnea increases the risk of heart attack, stroke, arrhythmias, and persistent high blood pressure. The mask prevents those breathing pauses and allows your body to rest properly.

Many patients find it uncomfortable at first, but most adapt within a few weeks. Proper mask fitting makes a big difference. Using it consistently is what protects your heart and improves your daytime energy.”

CASE-7

1) Diagnosis & Differential Diagnosis

Most Likely Diagnosis:
Coarctation of the Aorta (post-ductal type).

Pathophysiological justification:

- Upper extremity hypertension (165/90 mmHg)
- Lower extremity hypotension (110/70 mmHg)
- Arm–leg systolic BP gradient >20 mmHg
- Leg fatigue (claudication from reduced distal perfusion)
- Continuous murmur over the interscapular area (collateral flow)

This presentation is classic for aortic narrowing distal to the left subclavian artery.

Differential Diagnoses:

- Interrupted aortic arch (usually neonatal)
- Takayasu arteritis (systemic inflammatory signs)
- Severe peripheral arterial disease (unlikely at 18 years old)
- Subclavian stenosis (would not explain leg gradient)

---

2) Diagnostic Workup

Initial Imaging Test of Choice:
Transthoracic echocardiography (TTE) — first-line, especially in young patients.

It evaluates:

- Location and gradient across coarctation
- Associated bicuspid aortic valve (common association)
- LV hypertrophy

Further Imaging (for anatomy):

- CT angiography (CTA) or
- MR angiography (MRA)

Classic Chest X-ray Finding:

- **“Figure-of-3 sign”** (aortic indentation)
- Rib notching (from enlarged intercostal collateral arteries)

---

3) Management Plan

Definitive Management:

- **Endovascular stent placement** (preferred in adolescents/adults) or
- Surgical repair (if anatomy unsuitable for stenting)

Indications:

- Peak-to-peak gradient ≥20 mmHg
- Hypertension attributable to coarctation
- Significant collateral formation

Medical therapy:

- Beta-blockers or ACE inhibitors for BP control pre- and post-repair

---

4) Long-Term Follow-up

Coarctation requires **lifelong cardiology follow-up**, even after repair.

Monitoring Protocol:

- BP measurement in both arms and at least one leg at every visit
- Annual office visit
- Periodic ambulatory BP monitoring
- Echocardiography annually or every 1–2 years
- MRI/CTA every 3–5 years to evaluate:
    - Recoarctation
    - Aneurysm formation at repair site

Monitor for:

- Persistent hypertension
- Aortic aneurysm
- Bicuspid aortic valve disease

Exercise guidance should be individualized.

---

5) Patient Education

"Repairing the narrowing corrects the blood flow problem, but it doesn't always completely reset the blood vessels. Some patients continue to have high blood pressure because their arteries have adapted to years of increased pressure.

So while the structural problem may be fixed, you will still need lifelong monitoring. Many patients do very well after repair, but we follow you carefully to ensure there's no recurrence or late complications.

Your heart is much better protected after repair, but ongoing follow-up is essential."

CASE-8

1) Diagnosis & Differential Diagnosis

Most Likely Diagnosis:
Endogenous Cushing Syndrome (Hypercortisolism).

Pathophysiological justification:

- Rapid central weight gain (face, abdomen)
- Facial rounding ("moon face")
- Wide purple striae
- Easy bruising (skin collagen breakdown)
- Hypertension (160/100 mmHg)
- No exogenous steroid exposure

These features strongly indicate cortisol excess.

Differential Diagnoses:

- **Cushing disease** (pituitary ACTH adenoma – most common endogenous cause)
- **Adrenal cortisol-producing adenoma**
- **Ectopic ACTH syndrome**
- Severe obesity/metabolic syndrome (does not cause purple striae or easy bruising)
- Pseudo-Cushing states (depression, alcoholism)

---

2) Diagnostic Workup

Appropriate Initial Screening Test (choose one of the following):

- **1 mg overnight dexamethasone suppression test (DST)**
- Late-night salivary cortisol (×2)
- 24-hour urinary free cortisol (×2)

Most practical first-line: **1 mg overnight DST.**

If abnormal → repeat with a second confirmatory screening test before etiologic workup.

---

3) Management Plan

After confirming hypercortisolism:

- Measure ACTH to determine ACTH-dependent vs independent cause
- Imaging based on ACTH level (pituitary MRI vs adrenal CT)

If no tumor can be localized or surgery is not feasible:

Medical Treatment Options:

1. **Steroidogenesis inhibitors**
    - Ketoconazole
    - Metyrapone
    - Osilodrostat
2. **Pituitary-directed therapy (if ACTH-dependent)**
    - Pasireotide
    - Cabergoline
3. **Glucocorticoid receptor antagonist**
    - Mifepristone (particularly if diabetes prominent)

Choice depends on ACTH source, severity, comorbidities, and availability.

---

4) Long-Term Follow-up

Cushing syndrome requires structured lifelong monitoring.

If surgically treated:

- Morning cortisol post-op
- Monitor for adrenal insufficiency
- Gradual glucocorticoid taper if needed
- Clinical and biochemical evaluation every 3–6 months for first 2 years
- Then annually

If medically treated:

- Monitor cortisol levels every 4–6 weeks during titration
- Liver function tests (if ketoconazole)
- Electrolytes
- Blood pressure, glucose, weight
- Annual pituitary/adrenal imaging if indicated

Also monitor:

- Bone density (DEXA)
- Cardiovascular risk
- Psychiatric symptoms

---

5) Patient Education

“The facial fullness and stretch marks are caused by prolonged exposure to high cortisol levels. Once cortisol levels are normalized, many changes improve gradually.

The ‘moon face’ often improves over several months. The purple stretch marks typically fade in color but may not disappear completely, especially if they are wide.

The most important step is controlling the cortisol excess. Once that is treated, your body will slowly recover, and many of these physical changes will improve over time.”

CASE-9

1) Diagnosis & Differential Diagnosis

Most Likely Diagnosis:
Diabetic Kidney Disease (Diabetic Nephropathy) causing secondary hypertension.

Pathophysiological justification:

- Long-standing Type 1 Diabetes
- Hypertension (155/95 mmHg)
- Foamy urine (proteinuria)
- 3+ protein on dipstick
- Mildly elevated creatinine (1.4 mg/dL)

Hyperglycemia → glomerular hyperfiltration → mesangial expansion → basement membrane thickening → albuminuria → progressive CKD → RAAS activation → hypertension.

Differential Diagnoses:

- Non-diabetic glomerulonephritis (especially if rapid onset or active sediment)
- Focal segmental glomerulosclerosis
- IgA nephropathy
- Hypertensive nephrosclerosis (less likely at 28 without long-standing HTN)

In Type 1 DM, albuminuria strongly suggests diabetic nephropathy unless atypical features exist.

---

2) Diagnostic Workup

Most Important Calculation:
Urine Albumin-to-Creatinine Ratio (UACR)

and
Estimated Glomerular Filtration Rate (eGFR).

These allow staging per KDIGO classification:

- CKD stage based on eGFR
- Albuminuria category (A1, A2, A3)

Quantification is essential for prognosis and management.

---

3) Management Plan

Specifically Indicated Antihypertensive Class:
ACE inhibitor or ARB.

Rationale:

- Reduces intraglomerular pressure
- Decreases proteinuria
- Slows CKD progression
- Independent renoprotective effect

BP target (per KDIGO/ESH):

- <130/80 mmHg (if tolerated)

Additional measures:

- Tight glycemic control (HbA1c individualized ~7%)
- Sodium restriction
- Lipid management (statin if indicated)

Avoid dual ACEi + ARB therapy.

---

4) Long-Term Follow-up

Monitoring Frequency:

- UACR: every 3–6 months
- eGFR: every 3–6 months (more often if progressive decline)
- Serum potassium and creatinine: 1–2 weeks after ACEi initiation or dose change
- BP monitoring at every visit

Annual:

- Lipid panel
- Fundoscopy
- Neuropathy screening

Refer to nephrology if:

- eGFR <30
- Rapid decline (>5 mL/min/year)
- Nephrotic-range proteinuria

---

5) Patient Education

"Protein in your urine does not automatically mean your kidneys are failing. It means the kidney filters are becoming leaky. This is often an early sign of diabetic kidney involvement.

The good news is that when we detect this early, we can slow or even stabilize the process with proper blood pressure control, blood sugar management, and specific medications like ACE inhibitors.

Our goal is to prevent progression. Many patients live for decades with stable kidney function when treated appropriately."

CASE-10

1) Diagnosis & Differential Diagnosis

Most Likely Diagnosis:
Fibromuscular Dysplasia (FMD) involving renal and carotid arteries.

Pathophysiological justification:

- Young woman (classic demographic)
- Severe hypertension (165/105 mmHg)
- Carotid bruit + epigastric (renal) bruit
- Pulsatile tinnitus ("whooshing" sound)
- Normal renal function

FMD is a non-atherosclerotic, non-inflammatory arteriopathy causing arterial stenosis, aneurysm, or dissection—commonly affecting renal and extracranial carotid arteries.

Differential Diagnoses:

- Atherosclerotic renal artery stenosis (unlikely at 28, no risk factors)
- Takayasu arteritis (would have systemic inflammatory features)
- Carotid dissection (acute focal neurologic signs typically present)
- Primary hypertension (would not explain bruits and tinnitus)

---

2) Diagnostic Workup

Why Renal Artery Duplex Ultrasound is Sometimes a "Trap":

Duplex ultrasound may miss FMD because:

- FMD typically affects the **mid-to-distal renal artery**, not the proximal segment
- Ultrasound visualization is limited distally
- Operator-dependent sensitivity

This can lead to **false reassurance** and delayed diagnosis.

Preferred Diagnostic Imaging:

- **CT Angiography (CTA)** or
- **MR Angiography (MRA)**

Classic finding: “String-of-beads” appearance.

If intervention planned → catheter-based angiography.

---

3) Management Plan

Preferred Treatment:
Percutaneous transluminal renal angioplasty (PTRA) without stenting.

Rationale:

- FMD responds well to balloon angioplasty
- Stenting usually not required
- High rate of BP improvement or cure in young patients

Medical therapy (if mild disease or residual HTN):

- ACE inhibitor or ARB
- Antiplatelet therapy (aspirin) recommended in cerebrovascular FMD

---

4) Long-Term Follow-up

FMD requires lifelong surveillance.

Post-angioplasty:

- BP check within 1–2 weeks
- Serum creatinine
- Duplex ultrasound at 6 months
- Then annually

Full vascular screening recommended:

- One-time head-to-pelvis imaging (CTA/MRA) to screen for aneurysms or dissections

Ongoing:

- Annual BP monitoring
- Periodic vascular imaging every 1–2 years depending on findings

Monitor for:

- Recurrence
- Aneurysm formation
- Arterial dissection

---

5) Patient Education

“You’re right to ask. In fibromuscular dysplasia, the arteries can be more fragile. High-impact neck trauma or forceful cervical manipulations can increase the risk of carotid artery dissection.

That does not mean you cannot exercise. Aerobic endurance sports like running are generally safe if your blood pressure is controlled. However, I would advise avoiding high-velocity neck manipulation (such as aggressive chiropractic adjustments).

We will tailor activity recommendations based on your imaging results, but in general, staying active is beneficial — we just avoid activities that risk sudden neck strain.”
