# Supplementary material for: Diagnostic Performance and Workup Efficiency of Large Language Models in Secondary Hypertension: A Blinded Comparative Study
Source: Diagnostics (Basel). 2026 Jul 10;16(14):2165. doi: 10.3390/diagnostics16142165 (PMC13409298; doi:10.3390/diagnostics16142165)
Supplement: Supplementary file 1 [file diagnostics-16-02165-s001.zip › Supplementary file S2/7. Model Access and Reproducibility Details.pdf]

Supplementary Appendix S1. Model Access and Reproducibility Details

All model queries were performed on **17 February 2026** within a predefined query window between **[18:30] and [22:30] [Türkiye local time, UTC+3]**. Each clinical vignette was submitted once to each model using a new independent session. No iterative prompting, response regeneration, clarification prompting, external document retrieval, internet browsing, or tool augmentation was permitted.

The following model access conditions were used:

Model	Provider	Access route	Model/version label recorded at access	Query date	Query time window	Temperature / inference setting
GPT-5.2	OpenAI	[Public Web Interface]	[GPT-5.2 as displayed in the public interface; exact backend version not disclosed]	17 February 2026	[18:30-22:30]	Default setting; no manual temperature adjustment
Claude Sonnet 4.6	Anthropic	[Public Web Interface]	[Claude Sonnet 4.6 as displayed in the public interface; exact backend version not disclosed]	17 February 2026	[18:30-22:30]	Default setting; no manual temperature adjustment
Gemini 3 Pro	Google	[Public Web Interface]	[Gemini 3 Pro as displayed in the public interface; exact backend version not disclosed”]	17 February 2026	[18:30-22:30]	Default setting; no manual temperature adjustment
