# Supplementary material for: Diagnostic Performance and Workup Efficiency of Large Language Models in Secondary Hypertension: A Blinded Comparative Study
Source: Diagnostics (Basel). 2026 Jul 10;16(14):2165. doi: 10.3390/diagnostics16142165 (PMC13409298; doi:10.3390/diagnostics16142165)
Supplement: Supplementary file 1 [file diagnostics-16-02165-s001.zip › Supplementary file S2/8. Supplementary Checklist Analysis items .pdf]

Supplementary Appendix 1

i- Supplementary Checklist Analysis items

Objective item	Scoring
Correct leading diagnosis	Absent/ Adequate
Important differential diagnoses included	Absent/ Adequate
Key screening test included	Absent/ Adequate
Confirmatory test included when needed	Absent/ Adequate
Imaging/subtyping strategy appropriate	Absent/ Adequate
Medication interference considered	Absent/ Adequate
Management plan guideline-concordant	Absent/ Adequate
Follow-up/monitoring appropriate	Absent/ Adequate
Unsafe or unsupported recommendation	Absent/ Adequate
