# Supplementary material for: Diagnostic Performance and Workup Efficiency of Large Language Models in Secondary Hypertension: A Blinded Comparative Study
Source: Diagnostics (Basel). 2026 Jul 10;16(14):2165. doi: 10.3390/diagnostics16142165 (PMC13409298; doi:10.3390/diagnostics16142165)
Supplement: Supplementary file 1 [file diagnostics-16-02165-s001.zip › Supplementary Table S1.pdf]

Supplementary Table S1. LLM code names and model correspondence used in the blinded evaluation.

Code Name	LLM Identity	Developer	Version
Earth	Claude Sonnet	Anthropic	4.6
Water	GPT	OpenAI	5.2
Air	Gemini Pro	Google	3

LLM: Large Language Model
